# Multimorbidity is associated with myocardial DNA damage, nucleolar stress, dysregulated energy metabolism, and senescence in cardiovascular disease

**DOI:** 10.1038/s41514-024-00183-z

**Published:** 2024-11-27

**Authors:** Kristina Tomkova, Marius Roman, Adewale S. Adebayo, Sophia Sheikh, Syabira Yusoff, Melanie Gulston, Lathishia Joel-David, Florence Y. Lai, Antonio Murgia, Bryony Eagle-Hemming, Hardeep Aujla, Tom Chad, Gavin D. Richardson, Julian L. Griffin, Gavin J. Murphy, Marcin J. Woźniak

**Affiliations:** 1 Department of Cardiovascular Sciences and NIHR Cardiovascular Biomedical Research Unit, University of Leicester, Glenfield Hospital, Leicester, UK; 2 Department of Biochemistry and Cambridge Systems Biology Centre, The Sanger Building, 80 Tennis Court Road, Cambridge, UK; 3 Biosciences Institute, Vascular Biology and Medicine Theme, Faculty of Medical Sciences, Newcastle University, Newcastle Upon Tyne, UK; 4 Present Address: Cardiovascular Sciences, King’s College London, London, UK; 5 Present Address: University of Aberdeen, King’s College, Aberdeen, UK

**Keywords:** Cardiovascular diseases, Senescence, Mitochondria

## Abstract

This study investigates why individuals with multimorbidity—two or more chronic conditions—are more prone to adverse outcomes after surgery. In our cohort, ninety-eight of 144 participants had multimorbidity. The myocardial transcriptome and metabolites involved in energy production were measured in 53 and 57 sequential participants, respectively. Untargeted analysis of the metabolome in blood and myocardium was performed in 30 sequential participants. Mitochondrial respiration in circulating mononuclear cells was measured in 70 participants. Results highlighted four main biological processes associated with multimorbidity: DNA damage with epigenetic changes, mitochondrial energy disruption, cellular aging (senescence) and innate immune response. Histone 2B, its ubiquitination enzymes and AKT3 were upregulated in the multimorbid group. Plasma senescence-associated proteins (IL-1β, GM-CSF) increased with more comorbidities. DNA damage and nucleolar instability were specifically apparent in multimorbid myocardium. We conclude that multimorbidity in cardiovascular patients accelerates biological aging, making them more vulnerable to metabolic stress.

## Introduction

Multimorbidity, defined as the presence of two or more comorbid conditions, affects over 50 million people in the European Union^1^. Multimorbidity is associated with frailty, increased susceptibility to stressors, functional decline after acute illness, and increased use of healthcare resources^2^. In the UK, multimorbidity is projected to affect two-thirds of adults aged over 65 years by 2035^3^. As research traditionally focuses on diseases in isolation, the mechanisms underlying multimorbidity are poorly understood^2^. Moreover, people with multimorbidity are often excluded from clinical trials of interventions and are therefore managed in the absence of high-quality evidence. Improving the lives of all people with multimorbidity is considered a national and global health research priority^4^.

Two-thirds of people with cardiovascular disease have multimorbidity. They suffer from larger infarct sizes after acute coronary syndrome (ACS), worse prognosis in heart failure, and attenuated effectiveness of organ protection strategies^5,6^. In cardiac surgery, multimorbidity affects 86% of people >65 years^7^, and is associated with a threefold increase in mortality^8^. As the population ages, the health burden attributable to cardiovascular disease in the setting of multimorbidity will increase. Recent reviews suggest chronic inflammation as the major factor driving the increased susceptibility to metabolic stress in multimorbidity^9^. It is likely responsible for epigenetic changes, the disruption of energy production, oxidative stress and senescence. The research into each of these processes usually involves isolated cells and animal models. However, no cell-based or animal model can replicate multifactorial multimorbidity in humans. This study fills this gap. Using untargeted transcriptomics and metabolomics in blood and myocardial samples from people with cardiovascular disease we identified specific processes associated with multimorbidity. The identified processes were further verified in targeted assays including direct measurement of mitochondrial respiration in circulating mononuclear cells.

## Results

### Study cohort

**Fig. 1:**
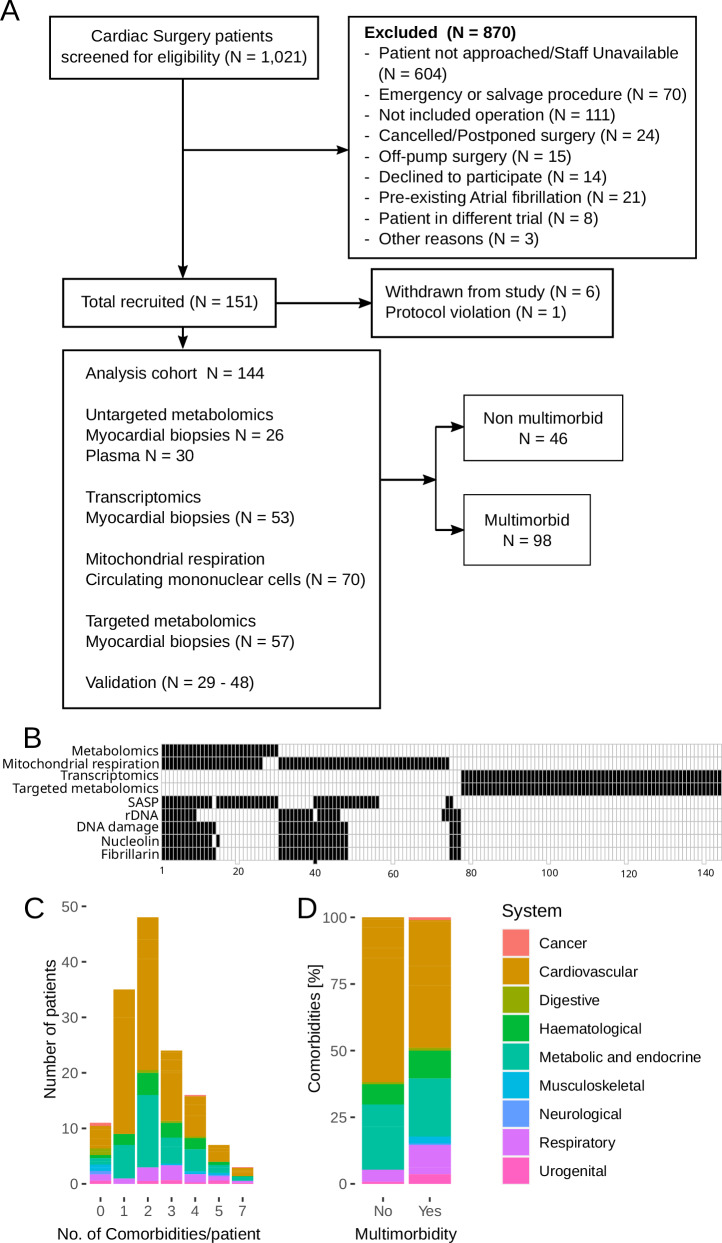
Cohort characteristics. **A** CONSORT diagram; **B** Breakdown of samples between the analyses. Black rectangles indicate that the analysis was performed. Tissue sources for the analyses were as follows: Metabolomics – blood plasma and myocardial biopsies, Mitochondrial respiration – blood mononuclear cells, Transcriptomics, targeted metabolomics, rDNA, DNA damage, Nucleolin and Fibrillarin – myocardial biopsies, SASP – blood plasma. **C** Comorbidities distribution in participants without or with a specific number of comorbidities; **D** Comorbidities distribution in participants without and with multimorbidity. Colors in C and D indicate the system affected by a comorbidity.

Out of 1021 people screened, 151 were recruited for this study. The reasons for exclusion are detailed in Fig. 1A. Six participants withdrew after the consent, and one was excluded for protocol deviation. Samples from 144 participants were analyzed. Analysis of the metabolome was performed in samples from 30 sequential participants. Measurements of transcriptome and metabolites involved in energy production were performed in 53 and 57 sequential participants, respectively, as previously described^10^. Mitochondrial function in circulating mononuclear cells was performed in 70 participants. Targeted validation was performed in samples from 29–48 participants and included analysis of senescence-associated secretory phenotype proteins in plasma, as well as estimations of ribosomal DNA copy numbers, DNA damage and nucleolar structure in the myocardium (Fig. 1A, B).

Multimorbidity was defined as the presence of two or more of nineteen chronic conditions (Table 1). Ninety-eight participants (68%) suffered from at least two comorbidities (Fig. 1A). The number of comorbidities per patient ranged between none (11 participants) and seven (three participants) and peaked at two comorbidities per patient (N = 48, Fig. 1C, D). Hypertension (12% vs 0%), angina or previous MI (49% vs 9%), heart failure (12% vs 1%), extracardiac arteriopathy (8% vs 0%), diabetes (30% vs 3%), obesity (24% vs 3%), chronic obstructive pulmonary disease (COPD; 15% vs 1%) and anemia (19% vs 1%) were enriched in the multimorbid group (Table 1). Post-surgery, the multimorbid group experienced higher lactate levels, lower mean PiO2/FiO2 ratio indicative of lung injury **(**Fig. S1**)**, and 11 out of 12 cases of Acute Kidney Injury (Table S1).

**Table 1:** Distribution of comorbidities between the groups

Multimorbidity					
Comorbidity	No (n = 46)	Yes (n = 98)	p-value	Missing data	System
Cancer	0 (0%)	2 (1.39%)	1	0	Cancer
Stroke (CVA/TIA)	2 (1.39%)	10 (6.94%)	0.339	0	Cardiovascular
Hypertension	0 (0%)	17 (11.81%)	**0.001**	0	Cardiovascular
Angina/MI	13 (9.03%)	71 (49.31%)	**<0.001**	0	Cardiovascular
Hyperlipidemia	2 (1.39%)	6 (4.17%)	1	0	Cardiovascular
Heart failure	1 (0.69%)	17 (11.81%)	**0.013**	0	Cardiovascular
Extracardiac arteriopathy	0 (0%)	11 (7.64%)	**0.017**	0	Cardiovascular
Pulmonary hypertension	0 (0%)	1 (0.69%)	1	0	Cardiovascular
Ulcerative colitis	0 (0%)	1 (0.69%)	1	0	Digestive
Liver disease	0 (0%)	2 (1.39%)	1	0	Digestive
Diabetes	4 (2.78%)	43 (29.86%)	**<0.001**	0	Metabolic and endocrine
Obesity (BMI > 32)	4 (2.78%)	34 (23.61%)	**0.001**	0	Metabolic and endocrine
Arthritis	0 (0%)	4 (2.78%)	0.306	0	Musculoskeletal
Osteoporosis	0 (0%)	1 (0.69%)	1	0	Musculoskeletal
Neurological disease	0 (0%)	1 (0.69%)	1	0	Neurological
Chronic obstructive pulmonary disease	1 (0.69%)	21 (14.58%)	**0.002**	0	Respiratory
Asthma	0 (0%)	5 (3.47%)	0.177	0	Respiratory
Renal disease	0 (0%)	8 (5.56%)	0.055	0	Urogenital
Anaemia	2 (1.39%)	28 (19.44%)	**0.001**	0	Hematological

### Metabolite analyses

Untargeted metabolomics was performed in myocardial biopsies and pre-surgery plasma samples. Myocardial biopsies from four participants were of insufficient quantity (<30 mg) and were not included in the analysis.

The myocardial biopsy dataset comprises 919 compounds, 820 of known identity and 99 of unknown structural identity. The plasma dataset comprises 1316 compounds, 1046 of known identity and 270 of unknown structural identity (Fig. S2A, B). All samples were comparable in numbers of known, unknown and undetected metabolites (Fig. S2A). The most numerous metabolites were lipids (376 in biopsies and 483 in plasma), followed by amino acids (164 in biopsies and 216 in plasma, Fig. S2B). There was no difference in the number of detected myocardial metabolites and their average peak area between participants with multimorbidity and without. The number of metabolites detected in plasma was significantly higher in participants with multimorbidity, although their average peak area was not different (Fig. S2C).

**Fig. 2:**
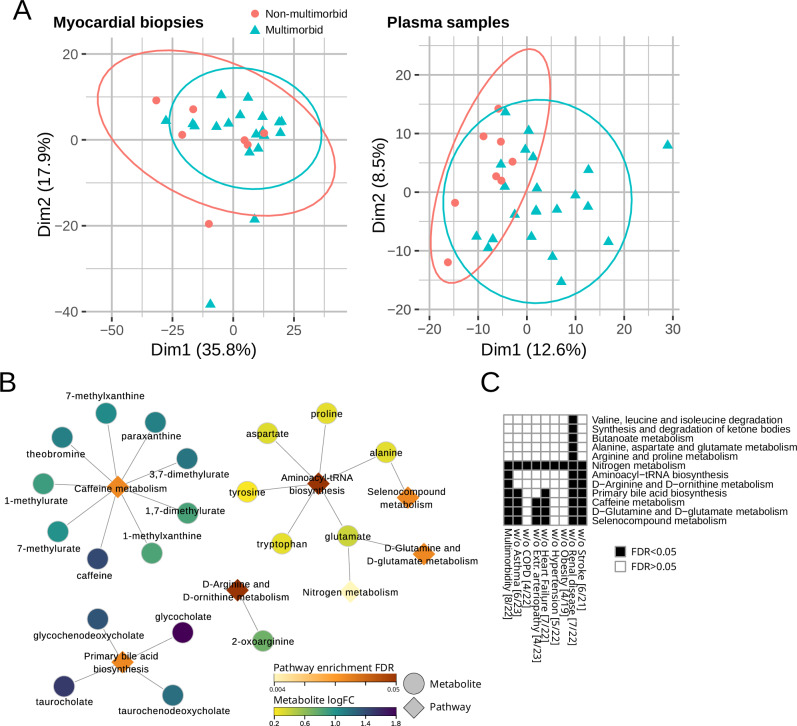
Metabolite analysis. **A** Principal component (1 and 2) plots for the detected metabolites in the myocardial biopsies and plasma. **B** KEGG metabolite set enrichment analysis in plasma samples with enriched metabolites. The color scales indicate metabolite log fold change (with positive number indicating upregulation in multimorbidity) or pathway enrichment p-value. **C** KEGG metabolite set enrichment analysis in plasma in complex multimorbidity and in reduced datasets to test the influence of each comorbidity and anti-diabetic medications. Black squares indicate significantly enriched pathways for specific dataset. Numbers in square brackets indicate the number of non-multimorbid/multimorbid patients.

There was a clear separation between multimorbid and non-multimorbid plasma samples in the principal component analysis. However, such distinction was not apparent for myocardial samples (Fig. 2A). Differential expression analysis identified 29 upregulated and eight downregulated metabolites in myocardial biopsies. One hundred and nine metabolites were upregulated and 15 downregulated in plasma (Fig. S2D). Since none of these metabolites passed the false discovery rate adjustment (Tables S2 and S3), we performed KEGG metabolite set enrichment analysis using the whole dataset. The analysis of myocardial biopsies did not return any significant pathways. Plasma metabolites significantly (FDR < 0.01) enriched Caffeine metabolism, Primary bile acid biosynthesis, Arginine and ornithine metabolism, Aminoacyl-tRNA biosynthesis, Glutamine and glutamate metabolism and Seleno compound metabolism (Fig. 2B). Caffeine metabolism, Aminoacyl-tRNA biosynthesis and Primary bile acid biosynthesis were enriched with 9, 6 and 4 metabolites, respectively, that differed (*p* < 0.05) between multimorbid and non-multimorbid groups. Levels of caffeine and its breakdown products, as well as bile acid intermediates, were higher in the multimorbid group, which indicates decreased expression or activity of cytochrome p450 enzymes, methylxanthine N1-demethylases, or choloylglycine hydrolase (Fig. S3A, B). Levels of amino acids that enriched the other pathways were lower in the multimorbid group (Fig. 2B and Table S3).

Sensitivity analyses to assess the influence of individual chronic conditions on our results (Fig. 2C**)** showed that Caffeine metabolism was affected by COPD, hypertension and Obesity; Primary bile acid biosynthesis was also affected by extracardiac arteriopathy; and Aminoacyl-tRNA biosynthesis was in addition affected by asthma. Due to the small sample size for metabolomics analyses we were not able to compare multimorbidity versus non-multimorbidity after excluding participants who had diabetes, anemia, or who were on diabetic medications or statins. Groups of patients with or without complex multimorbidity were identical to the primary multimorbidity classification.

### Transcripts analysis

**Fig. 3:**
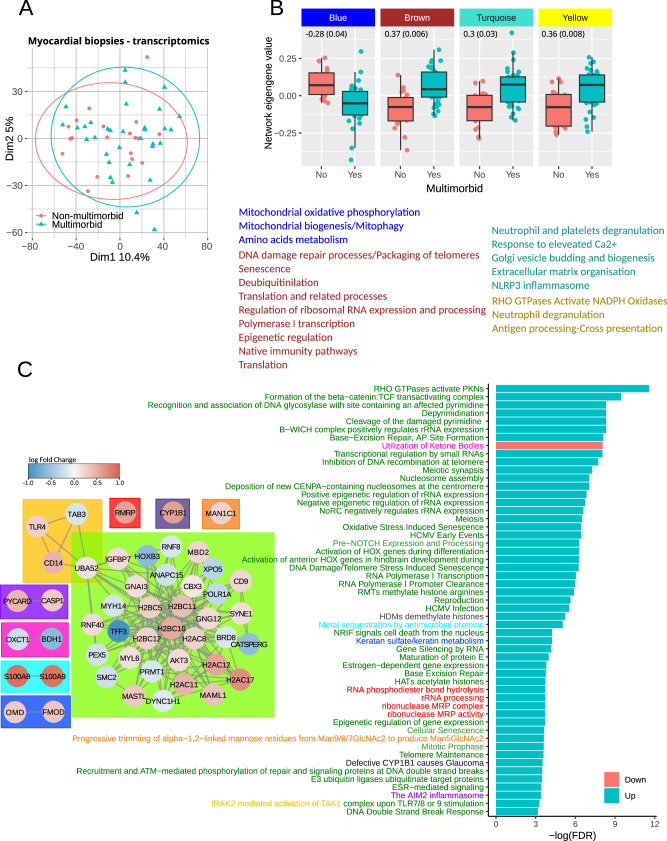
Myocardial transcripts analysis. **A** Principal component (1 and 2) plots for the myocardial transcriptomics profiles. **B** Network eigengene values distribution between the groups. Genes constituting each network were subjected to pathway enrichment analysis, and the summary is shown below the plots. Font colors indicate pathways enriched by genes in each of four networks. The full list of pathways is in Table S6. The numbers in each plot show a correlation estimate (p-value) between network eigengene values and the number of comorbidities. **C** Outcome of gene set enrichment analysis. The bar plot shows significantly regulated pathways (adjusted p-value < 0.05) specifically in multimorbidity but not the comorbidities The full list is in Table S7. The diagrams show transcripts sharing membership in at least one pathway. The node fill color shows transcripts’ log fold change with positive numbers indicating upregulation in multimorbidity. The colors in the node border indicate a number of pathways the transcript participates in. Box colors in the diagram and corresponding colors of pathway names indicate pathways sharing genes.

The transcriptomics analysis was performed on myocardial biopsies, and a summary of the dataset has been published previously^10^. Multimorbidity did not have a major effect on global gene expression patterns, as indicated by the principal component analysis (Fig. 3A). The differential expression analysis detected 854 transcripts (Table S4). Most upregulated transcripts encoded immunoglobulin chains.

None of the transcripts passed the multiple comparison adjustment, so we first performed a weighted gene correlation analysis to identify significantly changing groups of transcripts. The analysis identified one network of transcripts that was significantly downregulated (blue in Fig. 3B and Table S4) in the multimorbid myocardium and whose eigengene values significantly correlated with the number of comorbidities. Three networks were significantly upregulated and their eigengene values significantly correlated with the number of comorbidities (brown, turquoise and yellow in Fig. 3B and Table S4). The blue network included transcripts that enriched pathways involved in mitochondrial oxidative phosphorylation, mitochondrial biogenesis, mitophagy and amino acids metabolism. It included, among others, NUDS1, NUDV2, PDHB and OGHD. The brown network’s transcripts significantly enriched pathways involved in DNA damage processing and packaging, senescence, translation and regulation of ribosomal RNA expression. The transcripts included in this network were mainly ribosomal proteins, histone 2B and histone ubiquitin ligases. The turquoise and yellow networks included transcripts that significantly enriched innate immunity pathways. It included proteins required for neutrophil degranulation (LAMP1, PYCARD, FTL or RAB5C) but also proteins modulating inflammatory responses like serpins (A1, B1, E2, F1 and H1) and anti-apoptotic proteins like S100 (A4, A8, A9 and A11) or HMOX1. Transcript membership in each network is indicated in Table S4, and pathway enrichment results are in Table S5.

We also performed gene set enrichment analysis using the whole gene expression dataset (Table S6). The bar plot in Fig. 3C shows pathways specific to multimorbidity that passed false discovery rate adjustment. Diagrams in the left panel show transcripts annotated to and linked by joint pathway membership. The significant transcripts involved in the enriched pathways mainly encode proteins regulating chromatin condensation and DNA repair: Ubiquitin-60S ribosomal protein L40 (UBA52), E3 ubiquitin-protein ligases RNF40, RNF8 and ANAPC15, Protein arginine N-methyltransferase 1 (PRMT1), Bromodomain-containing protein 8 (BRD8) and Serine/threonine-protein kinase greatwall (MASTL), Repressor methyl-CpG-binding domain protein 2 (MBD2), Chromobox protein homolog 3 (CBX3), Structural maintenance of chromosomes protein 2 (SMC2), Mastermind-like protein 1 (MAML1); transcripts involved in gene transcription and polymerase I-interacting proteins: DNA-directed RNA polymerase I subunit RPA1 (POLR1A), Homeodomain-containing DNA binding protein 3 (HOXB3); and transcripts encoding nuclear export and cytoskeletal proteins: Exportin-5 (XPO5), Myosin-14 (MYH14), Myosin light polypeptide 6 (MYL6), Nesprin-1 (SYNE1). The only downregulated pathway was the utilization of ketone bodies with two significantly downregulated transcripts: Succinyl-CoA:3-ketoacid coenzyme A transferase 1 (OXCT1) and D-β-hydroxybutyrate dehydrogenase (BDH1).

Inflammation-related pathways were upregulated in multimorbidity and all comorbidities but stroke. Mitochondrial electron transport chain, translation, protein import and biogenesis were also non-specific to multimorbidity and were downregulated in all comorbidities (Table S6).

Sensitivity analyses to assess the influence of individual long-term conditions on our results showed that upregulated and downregulated pathways were similar across all comparisons of multimorbidity versus no multimorbidity after the exclusion of individual comorbidities. Removing patients with angina or previous MI had the strongest effect on the affected pathways (37% pathway overlap with the full dataset Fig. S4). However, it also considerably reduced the number of samples (13 multimorbid and 6 non-multimorbid). The results of the analysis without patients receiving anti-diabetic medications showed 91% overlap with the analysis of the full dataset. The analysis of data without patients receiving statins identified only one significantly enriched pathway. However, the reduced dataset included only four patients with multimorbidity and three without, making the analysis inconclusive. Using the complex multimorbidity definition affected DNA damage repair, nucleosome assembly, rRNA expression and translation (60% pathway overlap with the full dataset).

### Respiration in circulating mononuclear cells

To assess the influence of multimorbidity on mitochondrial function, we measured respiration in blood mononuclear cells, which are considered a good sensor for metabolic stressors^11^.

**Fig. 4:**
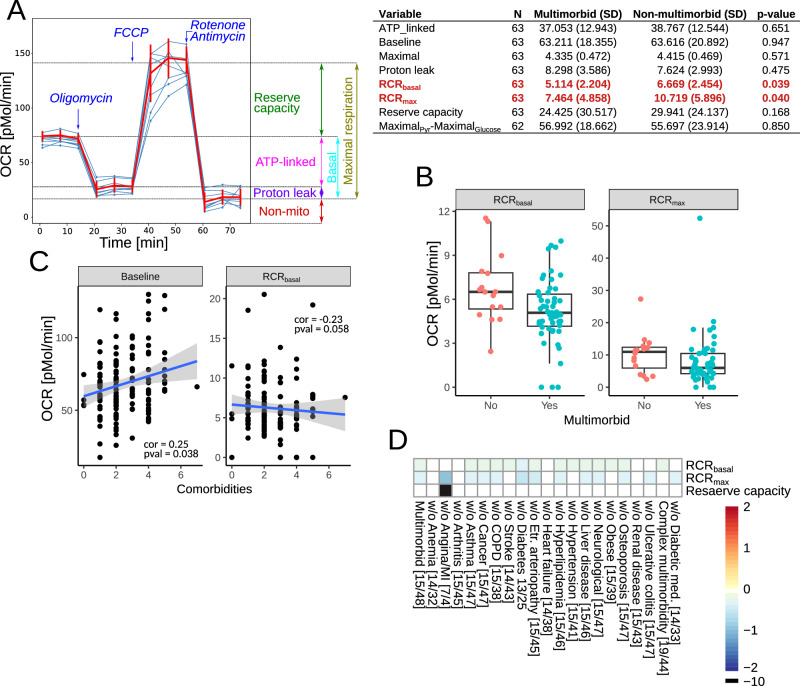
Mitochondrial respiration in circulating mononuclear cells. **A** The assays were performed in mononuclear cells isolated from blood samples without or with mitochondrial toxins: oligomycin (ATPase synthase inhibitor), FCCP (drug uncoupling ATP synthesis and electron transport), antimycin A (complex III inhibitor) and rotenone (complex I inhibitor). The summary of the measured OCR parameters is shown in the left panel. The right panel shows the oxygen consumption rate in mononuclear cells before and after surgery. Maximal_Pyr_-Maximal_Glucose_ is the difference between maximal respiration (in the presence of FCCP) in the presence of pyruvate or glucose. Significant parameters are in red. **B** Box plots of the significantly different mitochondrial parameters between multimorbid and non-multimorbid samples. **C** Plots of mitochondrial parameters significantly correlating with the number of comorbidities. **D** Analysis of OCR parameters in complex multimorbidity and in reduced datasets to test the influence of each comorbidity and anti-diabetic medications. Color scale shows log fold change, where positive numbers indicate higher levels in multimorbidity. Numbers in square brackets indicate the number of non-multimorbid/multimorbid patients.

The analysis of oxygen consumption rate (OCR) identified pre-operative basal (without any drugs) and maximal (in the presence of FCCP) respiratory control ratios (RCR) as significantly downregulated in the multimorbid samples (Fig. 4A, B). The RCR is a measure of mitochondrial coupling (see the Methods section for definitions), which links respiration to ATP synthesis. However, the RCR component measurements were not different between the groups. Therefore, we analyzed correlations of the number of comorbidities with OCR measurements. Significant correlations were found with baseline OCR and basal RCR (Fig. 4C).

The analysis of extracellular acidification rate (ECAR), which is a measure proportional to glycolysis, did not find any significant differences. To further test the role of glycolysis, we measured the difference in maximal OCR in the presence of glucose (glycolysis substrate) and pyruvate (glycolysis product). There was no difference in that measure, as well (Fig. 4A, Maximal_Pyr_-Maximal_Glucose_).

Sensitivity analyses to assess the influence of individual long-term conditions on our results demonstrated that anemia, arthritis, heart failure, and renal disease made the most significant contributions to reductions in Basal and Maximal RCR in multimorbidity versus no multimorbidity since removing these patients removed the significant difference we observed in the full dataset. Removing patients with angina from the dataset resulted in an increased difference in the maximal RCR between patients with multimorbidity and those without. In addition, the reserve mitochondrial capacity became significantly different between multimorbidity groups. However, it also considerably reduced the dataset (multimorbid n = 4, non-multimorbid n = 7). Participants not receiving anti-diabetic medications had lower maximal RCR. Analysis of the data without patients receiving statins was impossible due to the insufficient number of samples. Patients with complex multimorbidity versus no complex multimorbidity had lower basal RCR (Fig. 4D).

### Energy metabolism in multimorbidity

**Fig. 5:**
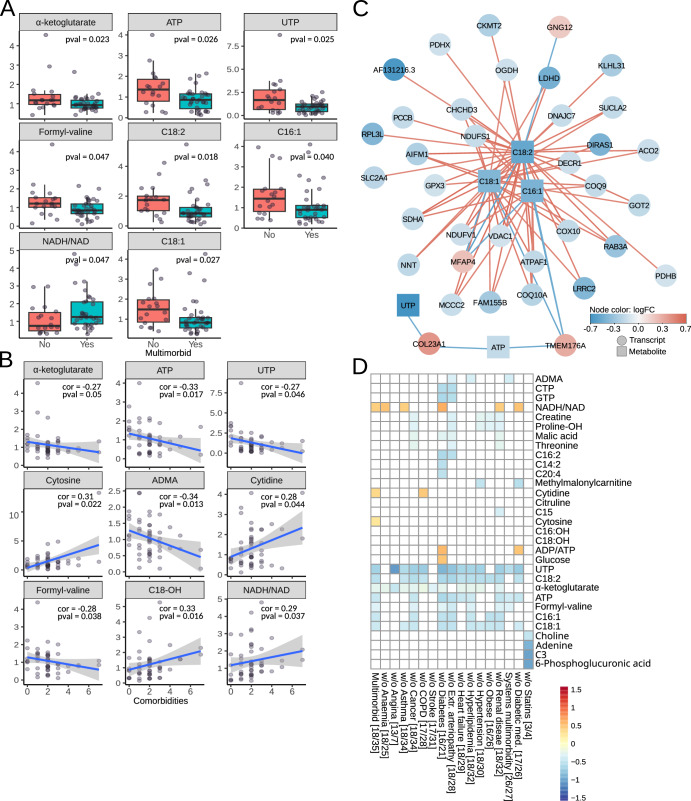
Myocardial metabolites involved in energy metabolism. **A** Targeted metabolites significantly different in multimorbid myocardium. **B** Targeted metabolites significantly correlating with the number of comorbidities. **C** Transcripts and targetted metabolites measured in paired samples were combined using sparse Partial Least Squares models with 0.6 cutoff. Node color indicates expression log fold change in the multimorbid group. Red edges show positive and blue edges show a negative correlation between nodes. **D** Analysis of targeted metabolites in complex multimorbidity and in reduced datasets to test the influence of each comorbidity and anti-diabetic medications. Color scale shows log fold change, where positive numbers indicate higher levels in multimorbidity. Numbers in square brackets indicate the number of non-multimorbid/multimorbid patients.

Since we could not directly measure respiration in myocardial biopsies, we analyzed a set of metabolites involved in energy metabolism using targeted metabolomics. Out of 144 measured metabolites, α-ketoglutarate, ATP, UTP, long-chain acyl-carnitines and formyl-valine significantly increased in multimorbidity, and NADH/NAD^+^ ratio decreased (Fig. 5A). Further correlation analysis confirmed the role of α-ketoglutarate, ATP, UTP and formyl-valine, whose levels decreased with the number of comorbidities. Conversely, the NADH/NAD+ ratio increased with the number of comorbidities. In addition, the analysis identified cytosine, cytidine, and hydroxylated stearoylcarnitine (C18-OH) as positively correlated and asymmetric dimethylarginine (ADMA) as negatively correlated with the number of comorbidities (Fig. 5B).

The metabolites, which were significant in group-wise comparisons and correlation analysis, were combined with paired transcriptomics in weighted gene correlation analysis using correlation cutoffs above and below 0.6 and -0.6, respectively. The three long-chain acyl-carnitines (C18:1, C18:2 and C16:1) positively correlated mainly with transcripts involved in the citric acid cycle and respiratory electron transport chain: NUDS1 NUDV2, PDHB, OGHD, COQ10A, ACO2, SUCLA2, NNT, PDHX, SDHA, VDAC1 but also with solute carrier SLC2A4 and heart-specific ribosomal protein RPL3L. A negative correlation was found between Guanine nucleotide-binding protein subunit gamma-12 (GNG12), Microfibril-associated glycoprotein 4 (MFAP4) and Transmembrane protein 176 A (TMEM176A). ATP and UTP correlated negatively with TMEM176A and Collagen α-1 (XXIII) chain (Fig. 5C).

Sensitivity analyses to assess the influence of individual long-term conditions on our results (Fig. 5D) showed that reductions in ATP, UTP, α-ketoglutarate, and long-chain acyl-carnitines were consistently observed across most of the analyses. That was also true for the cohort without patients on anti-diabetic medications. The analysis of the dataset without patients receiving statins identified choline, adenine, C3-carnitine and phosphoglucuronic acid. As before, removing patients receiving statins significantly reduced the dataset (4 multimorbid and 3 non-multimorbid). Patients with complex multimorbidity had decreased levels of ATP, UTP, long-chain acyl-carnitines and ADMA versus those without complex multimorbidity.

### Senescence-associated secretory phenotype

Decreased levels of energy substrates and nucleoside triphosphates can indicate higher levels of senescence. This is supported by changes in senescence-associated processes and pathways, including downregulation of mitochondrial oxidative respiration and mitochondrial biogenesis and upregulation of inflammation or DNA damage response. Transcripts like IGFBP7 (Fig. 3D), TIMP1, collagen, laminin, CCL13 and C-C/C-X-C chemokines receptors (Table S4), showing differential regulation in the multimorbid myocardial samples, encode proteins whose expression changes in senescence and are part of the senescence-associated secretory phenotype (SASP)^12,13^. Therefore, we tested whether cytokines previously described as SASP proteins were upregulated in the pre-operative plasma samples. To do this, we analyzed a panel of 71 cytokines and chemokines in samples from 48 participants. The panel includes cytokines that are regulated by NF-κB or IL-1/NLRP3, which are major modulators and initiators of SASP expression^14,15^.

**Fig. 6:**
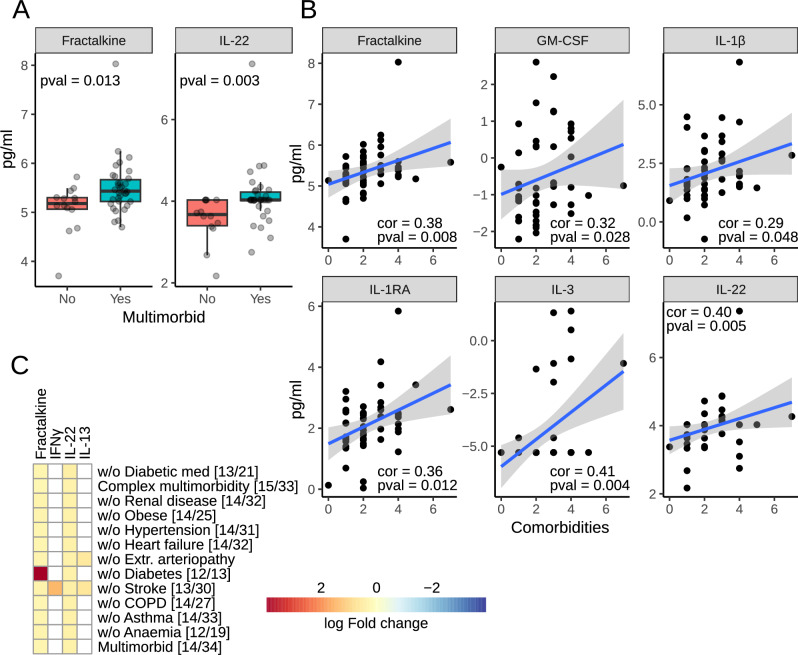
Senescence-associated secretory phenotype. **A** Box plots of circulating analytes significantly different in multimorbidity. **B** Plots of analytes correlating significantly with the number of comorbidities. **C** Analysis of the analyte panel in complex multimorbidity and in reduced datasets to test the influence of each comorbidity and anti-diabetic medications. The color indicates log fold change in multimorbidity. Numbers in square brackets indicate the number of non-multimorbid/multimorbid patients.

Groupwise comparison indicated that fractalkine and IL-22 were significantly upregulated in the multimorbid samples (Fig. 6A). Further correlation analysis with the number of comorbidities added GM-CSF, IL-1β, IL-1RA and IL-3 to the list (Fig. 6B). Downregulation of both fractalkine and IL-22 was not affected by any comorbidity or anti-diabetic medications, as indicated by the analysis in reduced datasets. Analysis of patients without statins was impossible due to the insufficient number of samples. Both proteins were also downregulated in complex multimorbidity (Fig. 6C).

### DNA damage and nucleolar assembly

Transcriptomics data suggested that the primary driver of senescence, DNA damage, is upregulated in multimorbidity. In addition, several transcripts encoding ribosomal proteins and others involved in polymerase I transcription and ribosomal RNA processing were affected (Fig. 3C, D). This can indicate changes in the expression of ribosomal RNA and dysregulation of nucleolar assembly, which can result in free ribosomal proteins, increased senescence levels and, consequently, changes in mitochondrial respiration^16^.

**Fig. 7:**
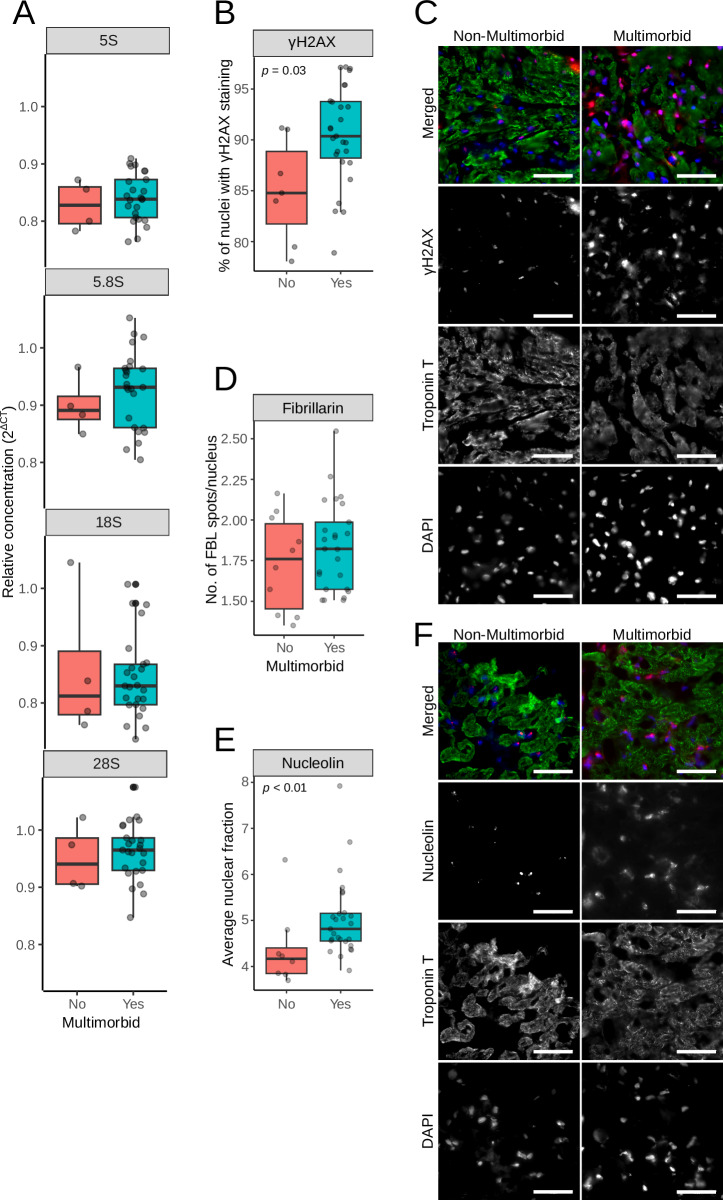
DNA damage and nucleolar stress in myocardium. **A** Ribosomal DNA copy numbers were analyzed by qRT-PCR with specific primers to genes encoding ribosomal DNA. The CT values were normalized against β-2-Microglobulin. **B** γH2AX was detected in cryosections of myocardial biopsies, and the numbers indicating the percentage of γH2AX-positive cells were plotted. **C** Representative images of cryosections labeled with γH2AX. **D** Nucleolin was detected in cryosection, and the plots show the percentage of nuclear area occupied by the nucleolin staining. **E** Representative images after the nucleolin labeling. Scale bars in C and E are 50 µm. **F** Fibrillarin was detected in cryosection, and plots show the number of nuclear fibrillarin spots. All assays were performed in mycardial samples.

We first tested ribosomal DNA (rDNA) copy numbers, as it was previously linked with mitochondrial abundance^17^. We analyzed rDNA in myocardial biopsies with qRT-PCR and specific primers, and as shown in Fig. 7A, we did not detect any difference in rDNA copy numbers between the groups. DNA damage was detected with antibodies against the phosphorylated form of histone 2AX (γH2AX) in myocardial cryo-slices. Multimorbid samples had significantly more nuclei positive for γH2AX (Fig. 7B, C). Next, nucleolar stress was assessed with antibodies against nucleolin and fibrillarin. Although fibrillarin staining patterns were not different in multimorbid samples (Fig. 7D), nucleolin labeling suggested higher levels of nucleolar stress as indicated by a larger fraction of nuclear area positive for nucleolin (Fig. 7E, F).

## Discussion

Using untargeted approaches, we identified processes specific to multimorbidity in people with cardiovascular disease that are characteristic of biological ageing. In the myocardium, these include changes at the transcriptomics level in chromatin organization pathways, DNA damage repair, nucleolar function and ketone bodies’ utilization in mitochondria. At the metabolite level, we observed decreased levels of products and substrates of mitochondrial oxidative phosphorylation. Mitochondrial function was also affected at the systemic level, as indicated by basal respiration that increased with the number of comorbidities. Patients with multimorbidity also had increased levels of caffeine, its breakdown products and intermediates of bile acid biosynthesis. Further validation experiments confirmed that senescence is associated with multimorbidity, as levels of several proteins of senescence-associated secretory phenotype increased with the number of comorbidities. We also confirmed that increased DNA damage and nucleolar instability play a role in multimorbidity.

The specifically enriched epigenetic pathways include histones and enzymes mediating post-translational histone modifications. The expression of histone 2B (H2B) and its E3 ubiquitin ligase (RNF40) and several other ubiquitin ligases were increased in the multimorbid group. H2B ubiquitination on K120 stimulates rapid changes in chromatin remodeling and transcriptional activity mediated by p53, a transcription factor involved in senescence^18^. Although we did not observe upregulation of CDKN1A (human p21 orthologue), several genes regulated by TP53 (human p53 orthologue) were affected. These include IGFBP7, TAB3 (TGF-β-activated kinase 1), AKT3 (RAC-gamma serine/threonine-protein kinase), ANAPC15, GNG12 (G protein subunit gamma 12)^19^, all of which are implicated in senescence. Dysregulation of the epigenetic regulation is also known to destabilize nucleolar function and ribosomal assembly, resulting in free ribosomal proteins that trigger senescence and alter DNA repair^16^. For example, free cytoplasmic ribosomal proteins L23, L29, S3 and S15 (upregulated in our dataset) bind MDM2, a TP53 ubiquitin ligase, enabling senescence-specific gene expression^16^. Free nuclear RPS3 binds to oxidative lesions in DNA and inhibits DNA repair^20^, and RPL3 upregulates CDKN1A inducing mitochondrial-driven apoptosis^21^.

Transcriptional changes demonstrated enrichment of senescence-linked pathways, which was confirmed by analysis of cytokines and chemokines in plasma. Several members of the senescence-associated secretory phenotype (IL-1β, IL-1RA and GM-CSF^12^) correlated positively with the number of comorbidities. Fractalkine, a chemokine whose levels increased in the multimorbid group and correlated with the number of comorbidities, was previously reported to decrease in a mouse model after senolytic treatments^13^. Levels of IL-22 showed a similar pattern to fractalkine. IL-22 is released by T cells as part of the tissue repair response^22^, and its overexpression in hepatic cells induced senescence in a p53 and p21-dependent manner^23^.

Caffeine and its breakdown products accumulated in the heart tissue and plasma before and after surgery. It is normally broken down in the liver’s endoplasmic reticulum by cytochrome p450 enzymes, followed by further processing in lysosomes before secretion in urine. The effect could be a consequence of a changed NADH/NAD+ ratio, which influences the activity of cytochrome p450 enzymes and, consequently, oxidation of fatty acids and steroids, whose expression changes were also observed in plasma. Cytochrome p450 enzymes are often affected in polypharmacy and are closely associated with aging^24^. Several of them showed decreased expression in the myocardium in the multimorbid group.

Biological ageing, a cluster of cellular changes implicated in the pathogenesis of age-related diseases^25^, is associated with increased susceptibility to metabolic stress in experimental models. In cardiac surgery, people with multimorbidity demonstrate increased susceptibility to the metabolic stress of surgery. Our observations lead us to hypothesize that biological ageing in human myocardium at baseline is a critical determinant of post-surgery susceptibility to organ dysfunction and morbidity. It follows that therapeutic interventions that target cellular ageing, or that prevent or reverse DNA damage, may have organ protection effects in surgery. This is entirely novel and has translational relevance given that existing organ protection strategies targeting hematological activation, the systemic inflammatory response to surgery, or tissue hypoxia, which have shown consistently negative clinical results in trials^26–28^.

The analysis has several strengths. First, the hypothesis was tested a priori using unbiased techniques. Second, the results of the untargeted analyses were consistent between omics platforms and were reproduced across different targeted assays. Our findings also agree with contemporary reviews of the pathogenesis multimorbidity. Third, to our knowledge, this is the first evaluation of mechanisms underlying multimorbidity in human myocardium. Almost all of the existing evidence is derived from animal studies or analyses of human blood.

Important limitations are: First, the small sample size and the breadth of the data increase the likelihood of statistical error in our analyses. In addition, myocardial biopsies may not have been uniform in cell content and included fragments of blood vessels or fat tissue, which further adds to the heterogeneity between samples. Due to the small sizes of the biopsies, it was impossible to estimate the variation in the cell composition. In mitigation, fluorescent image analysis indicates that most cells were cardiomyocytes. However, we cannot exclude the possibility that cells other than cardiomyocytes reduced differences in the multimorbid group, as different cell types differ in gene expression in response to multimorbidity^29^. Further studies, taking advantage of single-cell technologies, which can separate cells in silico, will help better define differences in the multimorbid group depending on the cell type. Second, we used a widely used consensus definition of multimorbidity^30,31^. This definition is limited because it is likely that clusters of chronic conditions not reflected in this definition lead to specific multimorbidity phenotypes. However, comparison to the complex multimorbidity, where two or more physiological systems are affected, generally supported our initial findings. We also addressed the potential confounding effects of drugs known to affect inflammation and biogenesis in sensitivity analyses where participants receiving these drugs were excluded and the analyses repeated. This did not change the results in any of our analyses; transcriptomic, targeted metabolomic, or mitochondrial function. This leads us to conclude, on the basis of the available evidence, that anti-diabetic drugs did not confound our analyses. The sensitivity analyses without statins were mainly inconclusive because the majority of patients in our cohort received these drugs. Statins are commonly prescribed to cardiac surgery patients^32^, therefore our analysis reflect changes caused by multimorbidity in this particular population.

Our findings are limited to multimorbid patients with cardiovascular disease and may not apply to a wider population of people with multimorbidity. Moreover, the recruited cohort was at low risk even for cardiac surgery; only 8% of participants developed acute kidney injury, whereas >25% is commonly observed in unselected cardiac surgery populations^33^. This resulted in only small differences in adverse outcomes post-surgery between the multimorbid and non-multimorbid groups and was likely a factor that prevented us from detecting significant changes at transcript and metabolite levels in the untargeted analyses. A high-risk surgery cohort, or multimorbidity in older, sicker patients presenting with ACS, may have resulted in a stronger effect size. The fact that almost all changes observed in the multimorbid group were positively associated with the number of comorbid conditions supports this conjecture. Furthermore, the cardiac surgery population is heterogeneous and larger epidemiological studies that cluster patients into specific phenotypes will better characterize mechanisms of multimorbidity. Third, we excluded patients with pre-existing paroxysmal, persistent or chronic atrial fibrillation or pre-existing inflammatory states. These are well-defined conditions with characteristic phenotypes and a strong effect size that could introduce important heterogeneity in a small sample. In mitigation, these comorbidities are extremely rare, and the recruited cohort is typical of the normal adult cardiac surgery population. Fourth, we were not able to perform all analyses on the same samples, which could potentially influence the conclusions we drew from different experiments. That is due to small biopsy sizes and their availability. However, the outcomes were consistent across different experiments, pointing towards increased DNA damage and affected mitochondrial nucleolar and mitochondrial function. Our paired transcriptomics and targeted metabolomics analysis indicated that long-chain acyl-carnitines correlate with several mitochondrial genes, which was further supported by decreased respiratory control ratios in unpaired analysis. However, the results of the correlation analyses need to be taken with caution since none of the transcripts passed false discovery rates. The transcriptomics identified sets of genes and pathways that indicate senescence-like changes in gene expression. That was confirmed by increased DNA damage and potential dysregulation of the nucleolar function in myocardial biopsies. The possible role of senescence in multimorbidity at the systemic level was also indicated by changes in circulating levels of several SASP members, although their expression was not affected in the heart tissue.

A prospective multi-omics analysis of human blood and myocardium obtained from a cohort undergoing cardiac surgery has identified multiple hallmarks of biological aging associated with multimorbidity, with epigenetic changes and cellular senescence being specific. Many of these processes, including mitochondrial dysfunction, chronic inflammation, and cell senescence, are modifiable by existing treatments.

## Methods

### Study design

A prospective observational case-control study to identify the role of epigenetic regulation of genes responsible for energy metabolism and mitochondrial function in the obesity paradox in cardiac surgery (ObCARD) was approved by The East Midlands – Nottingham 1 Research Ethics Committee. All participants provided written informed consent. The study protocol was registered at clinicaltrials.gov; NCT02908009. A primary analysis of this data has been reported previously^10^. This report is a secondary analysis, pre-specified in the study protocol. The study is reported as per the STrengthening the Reporting of Observational studies in Epidemiology (STROBE) statement. The STROBE checklist is included in the Supplementary Material (Table S7). The study adhered to the principles outlined in the Declaration of Helsinki.

### Study cohort

Adults (>16 years) undergoing coronary artery bypass grafting with or without valve surgery. Exclusions included pre-existing paroxysmal, persistent or chronic atrial fibrillation, pre-existing states likely to have hyperinflammatory phenotypes (sepsis undergoing treatment, acute kidney injury within five days, autoimmune diseases, chronic infection, congestive heart failure), ejection fraction <30%, pregnancy and in a critical pre-operative state (stage 3 AKI^34^ or requiring inotropes, ventilation or an intra-aortic balloon pump). Emergency or salvage procedures were also excluded. The study was designed to be hypothesis-generating, and no sample size was specified.

Multimorbidity was defined as per the Academy of Medical Sciences definition of two or more long-term (>1 year duration) conditions^30^. Obesity was defined as BMI > 32, based on our previous analysis^10^. Angina/MI was defined as Canadian Cardiovascular Society (CCS) angina grade II or higher or previous myocardial infarction. Recruitment was determined by the simultaneous availability of a consented patient undergoing surgery, clinical research staff, and laboratory research staff available to undertake analyses of fresh tissue and cells. Patients were recruited consecutively based on this availability.

### Sampling

Right atrial biopsies were collected in a standardized manner from the right auriculum before cannulation for cardiopulmonary bypass and were immediately snap-frozen in liquid nitrogen. Blood samples were processed within two hours of collection for the respiration analysis. The remaining plasma samples were frozen and stored at−80C.

### Measures taken to reduce bias

Selection bias was mitigated by the recruitment of sequential patients. Detection bias was mitigated by blinding laboratory staff who analyzed atrial biopsies and blood samples.

### RNA isolation and sequencing

RNA was isolated from 20 mg of tissue using Isolate II RNA mini kit (Bioline, London, UK). Sample quality was assessed using the RNA Screentape assay on the Agilent TapeStation 4200. Only samples with RNA integrity numbers equal to or greater than eight were sequenced.

Library preparation and sequencing were carried out in two batches by Source BioScience (Nottingham, UK). The stranded total RNA libraries were prepared in accordance with the Illumina TruSeq stranded total RNA sample preparation guide with Ribo-Zero human/mouse/rat for Illumina paired-end multiplexed sequencing. The libraries were validated on the Agilent Bioanalyzer 2100 to check the size distribution of the libraries and on the Qubit high sensitivity to check the concentration of the libraries. Sequencing was performed using 75 bp paired-end chemistry on HiSeq 4000 with the TruSeq stranded total RNA human kit.

#### Metabolomics

Untargeted metabolomics was performed by Metabolon, Inc. (USA). All samples passed appropriate quality controls.

For targeted metabolomics, a panel of 144 cellular metabolites involved in mitochondrial function and energy metabolism were analyzed on a Thermo Qunativa interfaced with a Vanquish LC as previously described^35^. In brief, tissue was extracted using a modified Folch extraction into chloroform/methanol (2:1 600 ul per 50 mg of tissue, followed by 200 ul of water, 200 ul of chloroform, repeated once). For nucleotides and acyl-CoA derivatives, one-half of the aqueous extract was dissolved in 150 µl of 70:30 acetonitrile:water containing 20 µM deoxy-glucose 6 phosphate and 20 µM [U–13 C, 15 N] glutamate. The resulting solution was vortexed, sonicated and centrifuged. Chromatography consisted of a strong mobile phase (A) was 100 mM ammonium acetate, and the weak mobile phase was acetonitrile (B) and the LC column used was the ZIC-HILIC column from Sequant (100 mm × 2.1 mm, 5 µm).

For amino acids and TCA cycle intermediates, aqueous extracts were reconstituted in 50 μl of 10 mmol/l ammonium acetate in water before TCA cycle intermediates were separated using reversed-phase liquid chromatography on a C18-PFP column (150 mm × 2.1 mm, 2.0 μm; ACE). For chromatography on the UHPLC system, mobile phase A was 0.1% formic acid in water, and mobile phase B was 0.1% formic acid in acetonitrile. Mass transitions of each species were as follows (precursor > product): D5-L-proline 121.2 > 74.2; D8-L-valine 126.1 > 80.2; D10-L-leucine 142.0 > 96.2; L-glutamate [M] 148.0 > 84.2; L-glutamate [M + 1] 149.0 > 85.2; L-glutamate [M + 6] 154.1 > 89.1; citrate 191.0 > 111.0; citrate [M + 1] 192.0 > 112.0; citrate [M + 2] 193.0 > 113.0; citrate [M + 3] 194.0 > 114.0; citrate [M + 4] 195.0 > 114.0; citrate [M + 5] 196.0 > 115.0; citrate [M + 6] 197.0 > 116.0. Collision energies and radio frequency (RF) lens voltages were generated for each species using the TSQ Quantiva optimization function.

**Mitochondrial respiration measurements** were performed in mononuclear cells isolated from pre-operative blood samples using the Histopaque method. Whole blood (5 ml) was spun throuth 5 ml Histopaque 1077 for 20 min at 1,600 rpm in an analytical centriguge. The cells were collected from the interface between plasma and Histopaqie 1077 and washed with PBS. Oxygen consumption (OCR) and extracellular acidification rates (ECAR) were measured in the absence or presence of 4 µM oligomycin, 2 µM FCCP or 2 µM rotenone and 2 µM antimycin A (Merck, UK) with Seahorse XFe24 analyzer (Agilent Technologies, USA) in 200,000 cells/well. To assess the effect of multimorbidity on glycolysis, OCR was measured in the presence of glucose or pyruvate as substrates. The respiratory control ratio was calculated as described in Hill et al. : RCR_max_ = (FCCP-Antimycin)/(Oligomycin-Antimycin); RCR_basal_ = (Basal-Antimycin)/(Basal-Antimycin)^36^ For each sample at least three parallel measurements were performed. Samples with poor background respiration or insufficient measurements were removed from the analysis. Samples were analysed on the day of collection and normalized against negative controls (no cells).

**Cytokine and chemokines** were measured in plasma samples collected before and 24 h after surgery using a panel of 71 cytokines/chemokines (Eve Technologies, Canada). All included samples passed quality control.

**Immunohistochemistry** was performed on cardiac biopsies. Tissue samples were frozen in OCT (Cell Path, UK) and sliced using Leica CM1520 cryostat (Leica Microsystems, UK) at 10-micron thickness. The slices were fixed in 10% neutral buffered formalin (Merck, UK) and permeabilized in ethanol (50%, 70%, and 100% EtOH). Unspecific binding was blocked with 1% BSA (Merck, UK). Samples were labeled with primary antibodies against nucleolin (rabbit polyclonal, abcam, UK), fibrillarin (rabbit polyclonal, abcam, UK), γH2AX (rabbit polyclonal, abcam, UK) or cardiac troponin T (mouse clone 1C11, abcam, UK). All primary antibodies were used at 1:300 dilution in Co-Detection Antibody Diluent (Advanced Cell Diagnostics, USA). The primary antibodies were detected with secondary Alexa Fluor 568 goat-anti-rabbit (Invitrogen, UK) and Alexa Fluor 488 goat-anti-mouse (Invitrogen, UK) antibodies at 1:200 dilution. Nuclei were labeled with DAPI (Thermo Fisher Scientific, UK). The sections were treated with Prolong Gold Anti-fade media (Invitrogen, UK), and visualized using an inverted Zeiss Axio Observer Z1 microscope equipped with Colibri 2 LED illumination, Plan-Apochromat 63x/1.40 oil objective and ORCA-Flash4.0 CMOS camera (Hamamatsu Photonics, Japan). For each patient’s sample, three slices were prepared, and approximately 20 images were collected per slice (400 – 500 cells). Images were analyzed using FIJI ImageJ distribution. Only the nuclear fraction of nucleolin staining and nuclear fibrillarin-positive particles were considered in the analysis.

**Quantitative real-time PCR** was used to estimate ribosomal DNA (rDNA) copy numbers. The genomic DNA was extracted from cardiac biopsies using a commercial kit following the manufacturer’s instructions (Genomic-tip 20/G and Genomic DNA Buffer Set, QIAGEN, UK). Specific primers for amplification of 5S, 5.8S, 18S, and 28S rDNAs were designed using NIH Primer-BLAST tool^37^ and synthesized by Merck (UK). As an endogenous PCR control, primers for the β-2-Microglobulin (B2M) gene were used. Primer sequences: rDNA 5S forward TCGTCTGATCTCGGAAGCTAA, reverse AAGCCTACAGCACCCGGTAT; rDNA 5.8S forward GAGGCAACCCCCTCTCCTCTT, reverse: GAGCCGAGTGATCCACCGCTA; rDNA 18S forward: AGCCTGAGAAACGGCTACCA, reverse: GGTCGGGAGTGGGTAATTTGC; rDNA 28S forward: CTCCGAGACGCGACCTCAGAT, reverse: CGGGTCTTCCGTACGCCACAT;

B2M forward primer TGCTGTCTCCATGTTTGATGTATCT, reverse primer TCTCTGCTCCCCACCTCTAAGT. For each 20 μl PCR reaction, 6 ng of genomic DNA was combined with 10 μl of PowerUp SYBR Green Master Mix (Thermo Fisher Scientific, UK), 1 μl of specific primers at 10 μM, and nuclease-free water. All reactions were set up in triplicates and measured using a Rotor-Gene Q qPCR machine (Qiagen, UK). Melt curves were produced for all measurements to confirm the presence of a single PCR product. The data were analyzed using the Rotor-Gene Q 2.1.0.9 software (Qiagen, UK).

#### Data processing and statistical analysis

Unless indicated otherwise, data analysis was performed with R Statistical Computing software version 4.2.2, and plots were prepared with the ggplot2 R package^38,39^.

*Transcriptomics* Sequencing data were quality-checked with FastQC v0.11.5, quantified with Salmon v1.21^40^ after indexing with a decoy and annotated with Ensembl v100. Gene quantities were normalized to length-scaled transcripts per million and filtered for low quantities before downstream analysis using the limma-voom model^41^ with empirical Bayes moderation. Sample groups were analyzed with the sequencing batch added to the model as a variable. The false discovery rate was set at 5%. Interactions within networks were visualized with Cytoscape^42^.

Weighted gene correlation network analysis^43^ was carried out to cluster differential genes into smaller modules with the soft r-squared set at >0.8 and gray modules excluded. Each module formed was subsetted from the adjacency matrix and exported as edge files for visualization in Cytoscape.

To directly analyze the well-known signaling pathways in the data, a gene-set analysis was carried out on the filtered transcriptome data using camera^44^ with Reactome and Gene Ontology annotations for transcripts and non-coding RNA, respectively, with false discovery rate set at 5%.

### Metabolomics

Untargeted metabolomic profiles were processed by Metabolome Inc. For targeted metabolite analysis, the peak area ratio of metabolites was obtained by integration within vendor software (Xcalibur QuanBrowser, Thermo Scientific, Hemel Hempstead, UK) and compared with isotopically labeled standards for quantification. Data were pre-processed by removing constant-value features, replacing zeros and missing values with half the smallest value in the entire dataset and removing extremely low relative standard deviation using Metaboanalyst v5.0^45^. The processed data was log-transformed and mean-normalized. Pairwise comparisons of sample groups were carried out using a t-test. Metabolite set enrichment analysis was performed with Metaboanalyst v5.0.

***Multiomics analyses*** of RNA and metabolite were combined using sparse Partial Least Squares (sPLS) models using mixOmics version 6.22.0^46^. Canonical correlation patterns and association networks derived from the components were then used to infer the relationship between genes and metabolites. The network analysis was visualized with Cytoscape.

**Sensitivity analyses** considered the effects of individual chronic conditions, effects of medications known to affect inflammation and biogenesis (statins or diabetes drugs including metformin) on the results by repeating each analysis after exclusion of single conditions or participants receiving these drugs. In addition, primary analyses were repeated using a more restrictive *complex multimorbidity* definition where two or more *physiological systems* are affected^47^.

## Supplementary information

Supplemental figures and tables

## Data Availability

Sequencing data are available via NCBI Gene Expression Omnibus (GSE159612). Metabolomics data are available through EMBL-EBI (MTBLS7259).

## References

[1] Navickas, R., Petric, V.-K., Feigl, A. B. & Seychell, M. Multimorbidity: what do we know? What should we do? *J. Comorb.* **6**, 4–11 (2016). 29090166 10.15256/joc.2016.6.72 PMC5556462

[2] Fortin, M., Soubhi, H., Hudon, C., Bayliss, E. A. & van den Akker, M. Multimorbidity’s many challenges. *BMJ* **334**, 1016–1017 (2007). 17510108 10.1136/bmj.39201.463819.2C PMC1871747

[3] Kingston, A. et al. Projections of multi-morbidity in the older population in England to 2035: estimates from the Population Ageing and Care Simulation (PACSim) model. *Age Ageing* **47**, 374–380 (2018). 29370339 10.1093/ageing/afx201 PMC5920286

[4] The Academy of Medical Sciences. Multimorbidity: a priority for global health research. (2018).

[5] Rucker, D. & Joseph, J. Defining the phenotypes for heart failure with preserved ejection fraction. *Curr. Heart Fail Rep.* **19**, 445–457 (2022). 36178663 10.1007/s11897-022-00582-x

[6] Ruiz-Meana, M. et al. Cardiomyocyte ageing and cardioprotection: consensus document from the ESC working groups cell biology of the heart and myocardial function. *Cardiovasc. Res.* **116**, 1835–1849 (2020). 32384145 10.1093/cvr/cvaa132

[7] Forman, D. E. et al. Multimorbidity in older adults with cardiovascular disease. *J. Am. Coll. Cardiol.* **71**, 2149–2161 (2018). 29747836 10.1016/j.jacc.2018.03.022 PMC6028235

[8] Stirland, L. E. et al. Measuring multimorbidity beyond counting diseases: systematic review of community and population studies and guide to index choice. *BMJ* **368**, m160 (2020). 32071114 10.1136/bmj.m160 PMC7190061

[9] Skou, S. T. et al. Multimorbidity. *Nat. Rev. Dis. Prim.* **8**, 48 (2022). 35835758 10.1038/s41572-022-00376-4 PMC7613517

[10] Adebayo, A. S. et al. Gene and metabolite expression dependence on body mass index in human myocardium. *Sci. Rep.* **12**, 1425 (2022). 35082386 10.1038/s41598-022-05562-8 PMC8791972

[11] Kramer, P. A., Ravi, S., Chacko, B., Johnson, M. S. & Darley-Usmar, V. M. A review of the mitochondrial and glycolytic metabolism in human platelets and leukocytes: Implications for their use as bioenergetic biomarkers. *Redox Biol.* **2**, 206–210 (2014). 24494194 10.1016/j.redox.2013.12.026 PMC3909784

[12] Coppé, J.-P., Desprez, P.-Y., Krtolica, A. & Campisi, J. The senescence-associated secretory phenotype: the dark side of tumor suppression. *Annu Rev. Pathol.* **5**, 99–118 (2010). 20078217 10.1146/annurev-pathol-121808-102144 PMC4166495

[13] Dookun, E. et al. Clearance of senescent cells during cardiac ischemia-reperfusion injury improves recovery. *Aging Cell* **19**, e13249 (2020). 32996233 10.1111/acel.13249 PMC7576252

[14] Acosta, J. C. et al. A complex secretory program orchestrated by the inflammasome controls paracrine senescence. *Nat. Cell Biol.* **15**, 978–990 (2013). 23770676 10.1038/ncb2784 PMC3732483

[15] Salminen, A., Kauppinen, A. & Kaarniranta, K. Emerging role of NF-κB signaling in the induction of senescence-associated secretory phenotype (SASP). *Cell Signal* **24**, 835–845 (2012). 22182507 10.1016/j.cellsig.2011.12.006

[16] Wang, W. et al. Ribosomal proteins and human diseases: pathogenesis, molecular mechanisms, and therapeutic implications. *Med. Res. Rev.* **35**, 225–285 (2015). 25164622 10.1002/med.21327 PMC4710177

[17] Gibbons, J. G., Branco, A. T., Yu, S. & Lemos, B. Ribosomal DNA copy number is coupled with gene expression variation and mitochondrial abundance in humans. *Nat. Commun.* **5**, 4850 (2014). 25209200 10.1038/ncomms5850

[18] Minsky, N. et al. Monoubiquitinated H2B is associated with the transcribed region of highly expressed genes in human cells. *Nat. Cell Biol.* **10**, 483–488 (2008). 18344985 10.1038/ncb1712

[19] Fischer, M. Census and evaluation of p53 target genes. *Oncogene* **36**, 3943–3956 (2017). 28288132 10.1038/onc.2016.502 PMC5511239

[20] Hegde, V., Yadavilli, S. & Deutsch, W. A. Knockdown of ribosomal protein S3 protects human cells from genotoxic stress. *DNA repair* **6**, 94–99 (2007). 17049931 10.1016/j.dnarep.2006.09.004

[21] Russo, A. et al. Human rpL3 induces G_1_/S arrest or apoptosis by modulating p21 (waf1/cip1) levels in a p53-independent manner. *Cell Cycle* **12**, 76–87 (2013). 23255119 10.4161/cc.22963 PMC3570520

[22] Dudakov, J. A., Hanash, A. M. & van den Brink, M. R. M. Interleukin-22: immunobiology and pathology. *Annu. Rev. Immunol.* **33**, 747–785 (2015). 25706098 10.1146/annurev-immunol-032414-112123 PMC4407497

[23] Kong, X. et al. Interleukin-22 induces hepatic stellate cell senescence and restricts liver fibrosis in mice. *Hepatology* **56**, 1150–1159 (2012). 22473749 10.1002/hep.25744 PMC3394879

[24] Doan, J., Zakrzewski-Jakubiak, H., Roy, J., Turgeon, J. & Tannenbaum, C. Prevalence and risk of potential cytochrome P450–mediated drug-drug interactions in older hospitalized patients with polypharmacy. *Ann. Pharmacother.* **47**, 324–332 (2013). 23482734 10.1345/aph.1R621

[25] López-Otín, C., Blasco, M. A., Partridge, L., Serrano, M. & Kroemer, G. Hallmarks of aging: an expanding universe. *Cell* **186**, 243–278 (2023). 36599349 10.1016/j.cell.2022.11.001

[26] Abbasciano, R. G. et al. Activation of the innate immune response and organ injury after cardiac surgery: a systematic review and meta-analysis of randomised trials and analysis of individual patient data from randomised and non-randomised studies. *Br. J. Anaesth.* **127**, 365–375 (2021). 34229833 10.1016/j.bja.2021.04.032

[27] Abbasciano, R. G. et al. Prophylactic corticosteroids for cardiopulmonary bypass in adult cardiac surgery. *Cochrane Database Syst. Rev.* **3**, CD005566 (2024). 38506343 10.1002/14651858.CD005566.pub4 PMC10952358

[28] Abbasciano, R. G. et al. Effects of interventions targeting the systemic inflammatory response to cardiac surgery on clinical outcomes in adults. *Cochrane Database Syst. Rev.* **10**, CD013584 (2023). 37873947 10.1002/14651858.CD013584.pub2 PMC10594589

[29] Plagg, B., Ehrlich, D., Kniewallner, K. M., Marksteiner, J. & Humpel, C. Increased acetylation of histone H4 at lysine 12 (H4K12) in monocytes of transgenic Alzheimer’s mice and in human patients. *Curr. Alzheimer Res.* **12**, 752–760 (2015). 26159193 10.2174/1567205012666150710114256 PMC4589156

[30] Quality standard [QS153]. Multimorbidity. https://www.nice.org.uk/guidance/qs153 (2017).

[31] Ho, I. S. S. et al. Measuring multimorbidity in research: Delphi consensus study. *BMJ Med.* **1**, e000247 (2022). 36936594 10.1136/bmjmed-2022-000247 PMC9978673

[32] Kulik, A. et al. Secondary prevention after coronary artery bypass graft surgery. *Circulation* **131**, 927–964 (2015). 25679302 10.1161/CIR.0000000000000182

[33] Karkouti, K. et al. Acute kidney injury after cardiac surgery: focus on modifiable risk factors. *Circulation* **119**, 495–502 (2009). 19153273 10.1161/CIRCULATIONAHA.108.786913

[34] Kidney Disease: Improving Global Outcomes (KDIGO) CKD Work Group. KDIGO 2012 clinical practice guideline for the evaluation and management of chronic kidney disease. *Kidney inter. Suppl*. **3**, 1–150 (2013).

[35] Charidemou, E. et al. A randomized 3-way crossover study indicates that high-protein feeding induces de novo lipogenesis in healthy humans. *JCI Insight* **4**, e124819 (2019). 31145699 10.1172/jci.insight.124819 PMC6629161

[36] Hill, B. G. et al. Integration of cellular bioenergetics with mitochondrial quality control and autophagy. *Biol. Chem.* **393**, 1485–1512 (2012). 23092819 10.1515/hsz-2012-0198 PMC3594552

[37] Ye, J. et al. Primer-BLAST: A tool to design target-specific primers for polymerase chain reaction. *BMC Bioinf.* **13**, 134 (2012). 10.1186/1471-2105-13-134 PMC3412702 22708584

[38] {R Core Team}. R: A Language and Environment for Statistical Computing. R Foundation for Statistical Computing (2018).

[39] Wickham, H. *Ggplot2: Elegant Graphics for Data Analysis*. (Springer, New York, NY, 2009). 10.1007/978-0-387-98141-3.

[40] Patro, R., Duggal, G., Love, M. I., Irizarry, R. A. & Kingsford, C. Salmon provides fast and bias-aware quantification of transcript expression. *Nat. Methods* **14**, 417–419 (2017). 28263959 10.1038/nmeth.4197 PMC5600148

[41] Ritchie, M. E. et al. limma powers differential expression analyses for RNA-sequencing and microarray studies. *Nucleic Acids Res.* **43**, e47 (2015). 25605792 10.1093/nar/gkv007 PMC4402510

[42] Shannon, P. et al. Cytoscape: a software environment for integrated models of biomolecular interaction networks. *Genome Res*. **13**, 2498–2504 (2003). 14597658 10.1101/gr.1239303 PMC403769

[43] Langfelder, P. & Horvath, S. Fast R functions for robust correlations and hierarchical clustering. *J. Stat. Softw.* **46**, i11 (2012). 23050260 PMC3465711

[44] Wu, D. & Smyth, G. K. Camera: a competitive gene set test accounting for inter-gene correlation. *Nucleic Acids Res.* **40**, e133 (2012). 22638577 10.1093/nar/gks461 PMC3458527

[45] Xia, J., Psychogios, N., Young, N. & Wishart, D. S. MetaboAnalyst: a web server for metabolomic data analysis and interpretation. *Nucleic Acids Res.* **37**, W652–W660 (2009). 19429898 10.1093/nar/gkp356 PMC2703878

[46] Lê Cao, K.-A., Martin, P. G., Robert-Granié, C. & Besse, P. Sparse canonical methods for biological data integration: application to a cross-platform study. *BMC Bioinforma.* **10**, 34 (2009). 10.1186/1471-2105-10-34 PMC2640358 19171069

[47] Ho, I. S.-S. et al. Examining variation in the measurement of multimorbidity in research: a systematic review of 566 studies. *Lancet Public Health* **6**, e587–e597 (2021). 34166630 10.1016/S2468-2667(21)00107-9

