# Supplementary material for: Multimorbidity is associated with myocardial DNA damage, nucleolar stress, dysregulated energy metabolism, and senescence in cardiovascular disease
Source: NPJ Aging. 2024 Nov 27;10(1):58. doi: 10.1038/s41514-024-00183-z (PMC11603063; doi:10.1038/s41514-024-00183-z)
Supplement: Supplementary file 1 — Supplemental figures and tables [file 41514_2024_183_MOESM1_ESM.pdf]

Multimorbidity is associated with myocardial DNA damage, nucleolar stress, dysregulated energy metabolism, and senescence in cardiovascular disease

Kristina Tomkova[1,6], Marius Roman[1,6], Adewale S Adebayo[1], Sophia Sheikh[1], Syabira Yusoff[1,4], Melanie Gulston[2], Lathishia Joel-David[1], Florence Y Lai[1], Antonio Murgia[2], Bryony Eagle-Hemming[1], Hardeep Aujla[1], Tom Chad[1], Gavin D Richardson[3], Julian L Griffin[2,5], Gavin J Murphy[1], Marcin J Woźniak[1]*

Supplementary material

Figure S1...2

Figure S2...3

Figure S3...4

Figure S4...5

Table S1 6

Table S2 7

Table S3 8

Table S4 9

Table S5 10

TableS6 11

TableS7 12

Figure S1

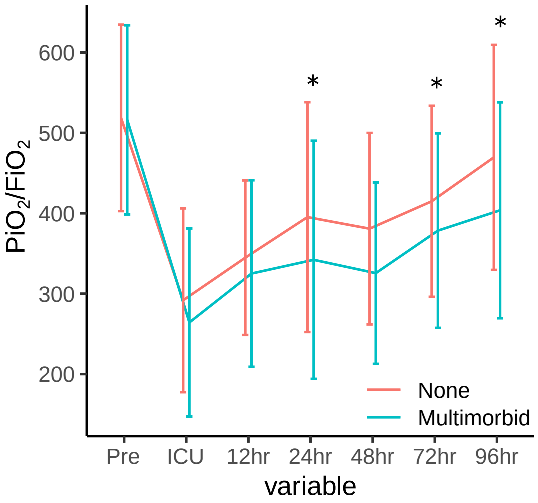

PiO2/FiO2 ratio before and after surgery. Asterisks indicate a significant difference (p-value < 0.05).

Figure S2

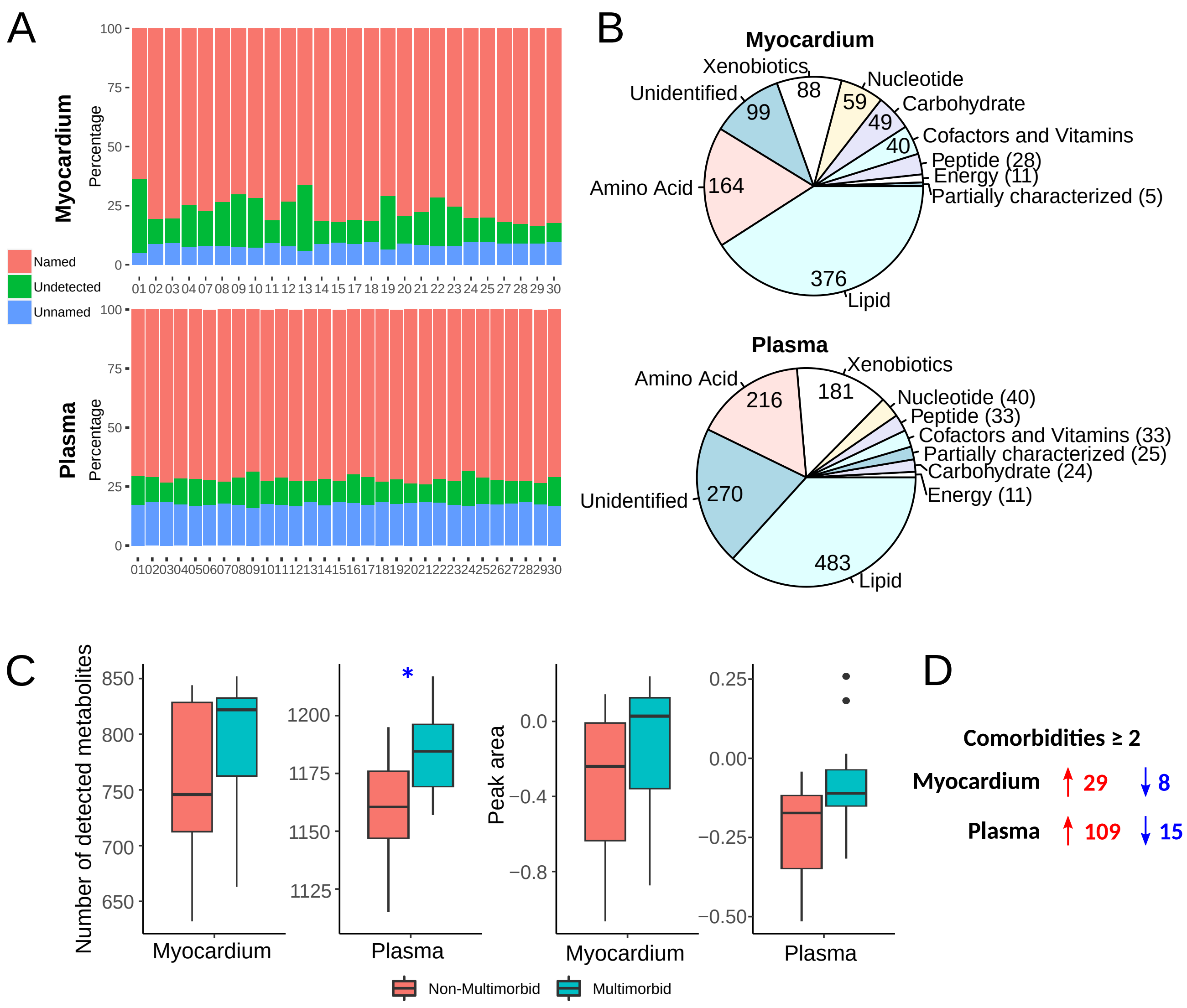

Figure S2 – Metabolomics summary A – Distribution of named, unknown and undetected metabolites in all sample types. **B** – Metabolites biotype distribution in all sample types. **C** – Comparison of detected metabolite numbers and average peak ratio in all sample types. **D** – summary of the differential expression analysis.

Figure S3

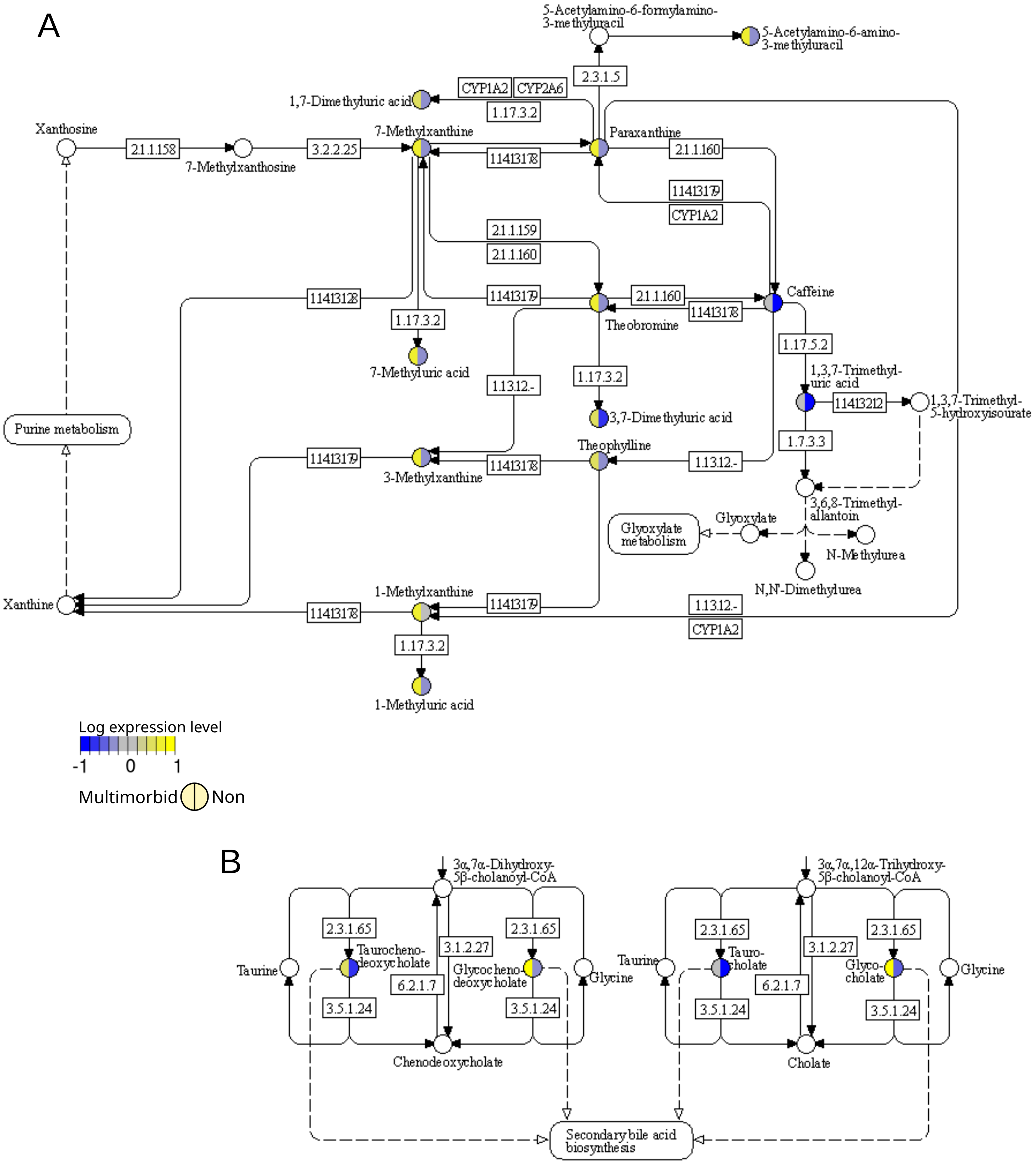

Figure S3 – Pathway overlay Expression of significantly different metabolites with membership in KEGG's Caffeine metabolism (**A**) and Primary bile acid biosynthesis (**B**) pathways.

Figure S4

A

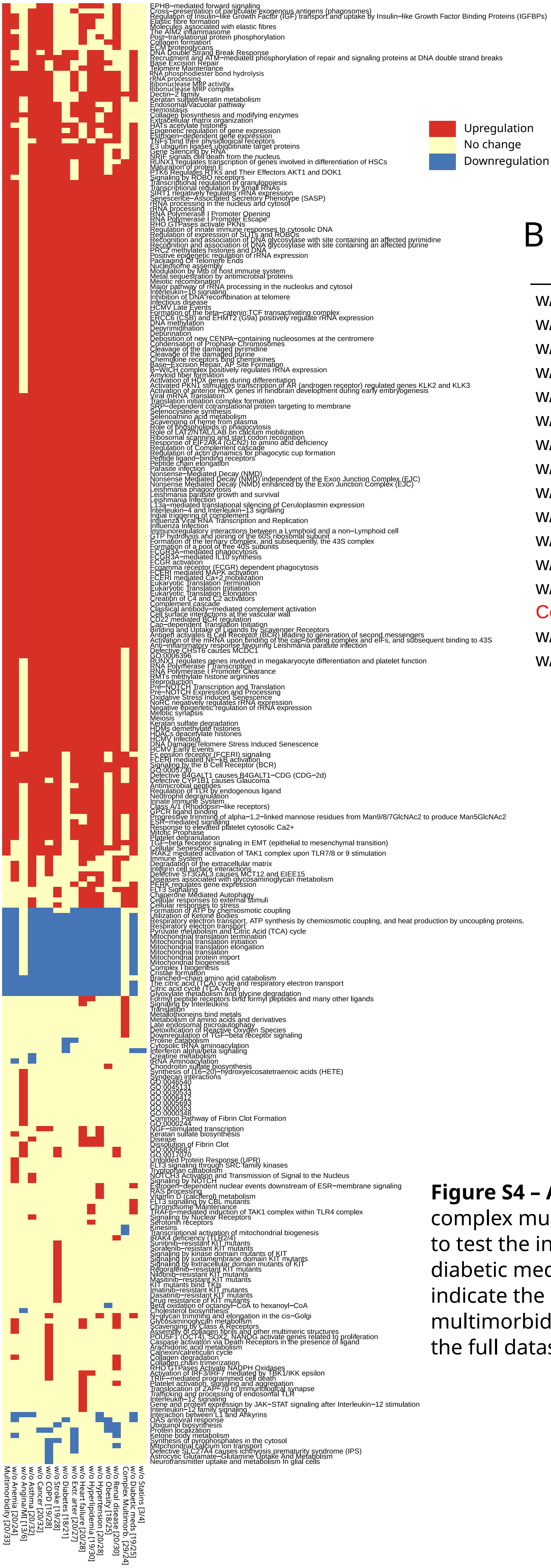

B

	Overlap with full dataset
w/o Anaemia	93.37%
w/o Angina	37.02%
w/o Asthma	98.90%
w/o Cancer	99.45%
w/o COPD	97.79%
w/o Stroke	90.61%
w/o Diabetes	76.80%
w/o Extr. Arter.	87.29%
w/o Heart failure	92.27%
w/o Hyperlipidemia	96.69%
w/o Hypertension	93.92%
w/o Obesity	79.56%
w/o Renal disease	96.13%
Complex multimorbidity	60.22%
w/o Diabetic meds	91.16%
w/o Statins	0.00%

Figure S4 – A - Gene set enrichment analysis in complex multimorbidity and in reduced datasets to test the influence of each comorbidity and anti-diabetic medications. Numbers in square brackets indicate the number of non-multimorbid/ multimorbid patients. **B** - Pathway overlap with the full dataset for each analysis.

Table S1 Patients' characteristics *Tests between the groups were conducted by exact test for categorical variables and t-test or non-parametric Wilcox test for continuous variables. Data is presented as n (%) for categorical variables and mean (standard deviation) or median (interquartile range) for continuous variables.

	All patients n=144	Multimorbidity (two or more comorbidities)			
		No (n=46)	Yes (n=98)	*p*-value	Missing data
Demographics	Age (years)	67.5 (61.25 - 75.5)	70 (63.25 - 73.75)	0.733	0
	Sex - male	40 (27.78%)	82 (56.94%)	0.804	0
	BMI	28 (25.25 - 29)	29 (25 - 32.75)	**0.042**	0
	Ethnic - White	39 (27.08%)	91 (63.19%)	0.254	0
	Smoking				
	never smoker	16 (11.11%)	36 (25%)		
	ex-smoker	25 (17.36%)	50 (34.72%)	0.967	0
	current smoker	5 (3.47%)	12 (8.33%)		
Medication	Statin	39 (27.08%)	83 (57.64%)	1	0
	Antidiabetic medication				
	Alogliptin	0 (0%)	1 (0.69%)		
	Gliclazide	0 (0%)	5 (3.47%)		
	Insulin	0 (0%)	6 (4.17%)	**0.011**	0
	Linagliptin	0 (0%)	1 (0.69%)		
	Metformin	2 (1.39%)	17 (11.81%)		
	Anti-platelet (5 days pre surgery)	34 (23.61%)	74 (51.39%)	0.839	0
	ACE inhibitor	16 (11.11%)	44 (30.56%)	0.281	0
Clinical characteristics	*Surgery type*				
	CABG	38 (26.39%)	86 (59.72%)	0.441	0
	CABG & Valve	7 (4.86%)	11 (7.64%)	0.441	0
	CABG & Valve & Other	0 (0%)	1 (0.69%)	0.441	0
	Valve & Other	1 (0.69%)	0 (0%)	0.441	0
	NYHA				
	class I	18 (12.5%)	21 (14.58%)		
	class II	27 (18.75%)	60 (41.67%)	**0.006**	0
	class III, IV	1 (0.69%)	17 (11.81%)		
	CCS				
	asymptomatic	10 (6.94%)	11 (7.64%)		
	class I	23 (15.97%)	16 (11.11%)		
	class II	11 (7.64%)	46 (31.94%)	**<0.001**	0
	class III	2 (1.39%)	14 (9.72%)		
	class IV	0 (0%)	11 (7.64%)		
	LVEF				
	Fair (30-49%)	5 (3.47%)	20 (13.89%)	0.237	0
	Good (>49%)	41 (28.47%)	78 (54.17%)		
	Left main stem diseased	10 (6.94%)	31 (21.53%)	0.242	0
	Extent of coronary disease				
	Normal	3 (2.08%)	3 (2.08%)		
	1VD	4 (2.78%)	2 (1.39%)	0.186	0
	2VD	11 (7.64%)	25 (17.36%)		
	3VD	28 (19.44%)	68 (47.22%)		
Preoperative characteristics	PaO2/FiO2 ratio baseline	495.26 (409.52 - 690.48)	457.18 (409.52 - 533.33)	0.572	0
	MABP baseline (mm Hg)	96.75 (87.25 - 104)	95.5 (88 - 102.75)	0.771	0
	Serum creatinine baseline (umol/L)	85 (76.25 - 95.75)	83.5 (75.25 - 94)	0.656	0
	Bilirubin baseline (umol/L)	10 (8 - 13)	10.5 (8 - 13)	0.889	3
	Platelets baseline (x109/L)	212.5 (171.25 - 236.5)	235 (186 - 269)	0.069	0
	Haemoglobin baseline (g/L)	142.5 (137.25 - 149)	140 (127 - 151)	0.107	0
	Haematocrit baseline (%)	0.42 (0.04)	0.41 (0.05)	0.169	0
	Red blood cells baseline	4.72 (4.43 - 5.01)	4.58 (4.23 - 4.91)	0.093	0
	White blood cells baseline	6.8 (5.8 - 8.68)	7.9 (6.73 - 9)	**0.021**	0
	Lymphocytes baseline	1.71 (1.45 - 2.26)	1.92 (1.47 - 2.36)	0.229	0
	Monocytes baseline	0.5 (0.38 - 0.6)	0.5 (0.4 - 0.61)	0.415	0
	Neutrophils baseline	4.26 (3.34 - 5.5)	5.01 (3.95 - 5.9)	**0.024**	0
	Eosinophils baseline	0.17 (0.11 - 0.29)	0.22 (0.13 - 0.31)	0.195	0
	Basophils baseline	0.04 (0.03 - 0.05)	0.04 (0.03 - 0.06)	0.089	0
Postoperative characteristics	PaO2/FiO2 ratio (CICU)	266.27 (221.74 - 317.97)	256.27 (193.7 - 351.78)	0.586	0
	MABP (CICU) (mm Hg)	75 (64.25 - 84.75)	72 (65 - 81)	0.581	0
	Serum creatinine (CICU) (umol/L)	78 (66.25 - 85)	78.5 (68 - 91.5)	0.433	0
	Bilirubin (CICU) (umol/L)	12 (9.25 - 15.75)	12 (10 - 16)	0.929	2
	Platelets (CICU) (x109/L)	162.5 (134 - 214)	179.5 (154 - 225.25)	0.079	2
	Haemoglobin (CICU) (g/L)	110.5 (105 - 122.75)	109 (101 - 120)	0.124	0
	Haematocrit (CICU) (%)	0.33 (0.04)	0.33 (0.05)	0.434	0
	Red blood cells (CICU)	3.72 (0.5)	3.62 (0.5)	0.255	0
	White blood cells (CICU)	13.35 (10.75 - 18.75)	14.85 (11.2 - 19.23)	0.442	0
	Lymphocytes (CICU)	1.21 (1.03 - 1.93)	1.43 (1.12 - 1.91)	0.336	0
	Monocytes (CICU)	0.6 (0.34 - 0.79)	0.66 (0.4 - 0.9)	0.182	0
	Neutrophils (CICU)	10.7 (8.17 - 14.41)	12.16 (8.82 - 15.81)	0.17	0
	Eosinophils (CICU)	0.15 (0.08 - 0.21)	0.12 (0.08 - 0.19)	0.481	1
	Basophils (CICU)	0.03 (0.02 - 0.04)	0.03 (0.02 - 0.05)	0.069	1
	Lactate (CICU) (mmol/L)	1.55 (1.1 - 2.1)	1.9 (1.4 - 2.5)	**0.013**	1
	MODS (CICU)	2 (1 - 3.5)	2 (1 - 3)	0.71	8
	Inotropic score 24h	0 (0 - 2)	0 (0 - 3)	0.418	8
	Vasoactive score 24h	7 (2.5 - 73.5)	7 (3 - 17)	0.841	8
	PaO2/FiO2 ratio 24hr	358.33 (300 - 409.52)	347.62 (263.07 - 456.93)	0.538	1
	MABP 24hr (mm Hg)	85.3 (80 - 95.5)	83 (75 - 95)	0.233	1
	PaO2/FiO2 ratio 48h	358.33 (301.79 - 409.52)	356.94 (273.31 - 410.85)	0.96	2
	serum creatinine 48h (umol/L)	77 (70 - 92.25)	74 (65 - 93)	0.439	3
	post-op RBC transfusion	14 (9.72%)	37 (25.69%)	0.457	0
	nonRBC transfusion >48h postop	8 (5.63%)	9 (6.34%)	0.162	2
	nonRBC transfusion <=48h	5 (3.68%)	12 (8.82%)	1	8
	PaO2/FiO2 ratio 48hr <=300	12 (8.33%)	30 (20.83%)	0.695	0
	AKI - kdigo criteria	1 (0.69%)	11 (7.64%)	0.104	0

Table S2 – Significantly regulated metabolites in myocardial biopsies. P-values for groupwise comparisons and correlations with the number of comorbidities are shown.

CHEMICAL_NAME	MM	SD.IQR_MM	nonMM	SD.IQR_nonMM	Fold_Change	pval	FDR	Correlation	Correlation pval	Type	HMDB
N-acetylaspartate (NAA)	0.054	0.833	-1.058	0.725	1.112	0.006	0.726	0.498	0.010	Amino Acid	HMDB0000812
5-acetylamino-6-amino-3-methyluracil	0.128	1.026	-1.004	0.502	1.131	0.009	0.726	0.655	0.000	Xenobiotics	HMDB0004400
gamma-glutamylalanine	0.148	0.700	-0.616	0.546	0.764	0.011	0.726	0.418	0.034	Peptide	HMDB0029142
3-hydroxydecanoylcarnitine	0.248	0.987	-0.656	0.617	0.904	0.013	0.726			Lipid	HMDB0061636
histidine betaine (hercynine)*	-0.591	1.169	0.484	1.197	-1.075	0.013	0.726	-0.403	0.041	Xenobiotics	HMDB0029422
N-acetyl-aspartyl-glutamate (NAAG)	0.160	0.644	-0.955	0.933	1.115	0.019	0.726	0.445	0.023	Amino Acid	HMDB0001067
fucose	-0.056	0.304	0.347	0.343	-0.403	0.021	0.726	-0.497	0.010	Carbohydrate	HMDB0000174,H[illegible]
pelargonate (9:0)	-0.100	0.288	0.437	0.465	-0.538	0.022	0.726	-0.499	0.009	Lipid	HMDB0000847
N1-methylinosine	-0.373	0.836	-1.201	0.421	0.828	0.022	0.726	0.606	0.001	Nucleotide	HMDB0002721
N-acetylglucosamine/N-acetylgalactosamine	-0.110	0.460	0.434	0.469	-0.544	0.024	0.726	-0.392	0.048	Carbohydrate	HMDB0000212,H[illegible]
glycerophosphorylcholine (GPC)	0.110	0.410	-0.179	0.195	0.289	0.028	0.726	0.392	0.048	Lipid	HMDB0000086
N6-succinyladenosine	-0.017	0.599	-0.504	0.285	0.488	0.029	0.726			Nucleotide	HMDB0000912
N6-carboxymethyllysine	0.034	0.627	-0.920	1.095	0.955	0.032	0.726	0.441	0.024	Carbohydrate	HMDB0240347
N,N,N-trimethyl-5-aminovalerate	-0.012	0.744	-1.049	1.098	1.037	0.032	0.726	0.482	0.013	Amino Acid	NA
malate	0.170	0.547	-0.509	0.637	0.679	0.033	0.726	0.467	0.016	Energy	HMDB0031518,H[illegible]
cystathionine	0.039	1.339	-1.241	1.157	1.280	0.033	0.726	0.430	0.028	Amino Acid	HMDB0000099
15-HETE	-0.241	1.338	0.809	0.880	-1.050	0.033	0.726			Lipid	HMDB0003876
threonylphenylalanine	-0.204	0.574	-0.565	0.000	0.360	0.035	0.726			Peptide	HMDB0029068
glycerophosphoethanolamine	0.053	0.638	-0.502	0.503	0.555	0.037	0.726			Lipid	HMDB0000114
hexanoylcarnitine (C6)	0.114	1.157	-0.866	0.883	0.980	0.037	0.726	0.416	0.035	Lipid	HMDB0000756
palmitoyl ethanolamide	0.007	0.388	0.249	0.259	-0.242	0.037	0.726			Lipid	HMDB0002100
guanidinoacetate	-0.118	0.637	-0.679	0.731	0.561	0.037	0.726	0.423	0.031	Amino Acid	HMDB0000128
methylmalonate (MMA)	0.314	0.618	-0.387	0.684	0.702	0.039	0.726			Lipid	HMDB0000202
ethylmalonate	0.131	0.819	-0.867	0.995	0.998	0.041	0.726	0.479	0.013	Amino Acid	HMDB0000622
valylglycine	-0.117	0.815	-0.800	0.579	0.683	0.046	0.726	0.466	0.017	Peptide	HMDB0029127
theobromine	-0.548	1.406	-1.782	1.174	1.234	0.046	0.726	0.492	0.011	Xenobiotics	HMDB0002825
sebacate (C10-DC)	-0.032	0.351	0.278	0.305	-0.310	0.047	0.726			Lipid	HMDB0000792
butyrylcarnitine (C4)	0.245	0.930	-0.791	1.070	1.036	0.048	0.726			Lipid	HMDB0002013
beta-hydroxyisovaleroylcarnitine	-0.095	0.762	-0.825	1.085	0.730	0.049	0.726	0.397	0.045	Amino Acid	NA
3-(4-hydroxyphenyl)lactate	-0.114	0.661	-0.743	0.855	0.629	0.049	0.726	0.442	0.024	Amino Acid	HMDB0000755
N-acetylmethionine sulfoxide	0.095	0.627	0.533	0.796	-0.439	0.049	0.726			Amino Acid	NA

Table S3 – Significantly regulated metabolites in pre-operative plasma. P-values for groupwise comparisons and correlations with the number of comorbidities are shown.

CHEMICAL_NAME	MM	SD.IQR_MM	nonMM	SD.IQR_nonMM	Fold_Change	pval	FDR	Correlation	Correlation pval	Type	HMDB	
1,3,7-trimethylurate	0.145	0.721	-0.979	0.489	1.124	0.000	0.119			Xenobiotics	HMDB0002123	
indolelactate	0.104	0.270	-0.320	0.224	0.424	0.001	0.152	0.412	0.024	Amino Acid	HMDB0000671	
pyrraline	0.411	0.531	-0.453	0.469	0.863	0.001	0.152			Xenobiotics	HMDB0033143	Yes
propionylcarnitine (C3)	0.156	0.262	-0.175	0.175	0.332	0.001	0.152	0.439	0.015	Lipid	HMDB0000824	
ferulic acid 4-sulfate	-0.656	1.111	-2.045	0.378	1.388	0.001	0.152	0.499	0.005	Xenobiotics	HMDB0029200	
glycocholate	1.338	1.164	-0.508	1.057	1.847	0.001	0.152	0.485	0.007	Lipid	HMDB0000138	
2-methoxyhydroquinone sulfate (1)	0.466	1.231	-0.942	0.749	1.408	0.001	0.152			Xenobiotics	NA	
N-acetyltyrosine	0.167	0.488	-0.282	0.200	0.449	0.001	0.152			Amino Acid	HMDB0000866	
2-aminoheptanoate	0.415	0.500	-0.081	0.264	0.496	0.002	0.152			Lipid	HMDB0094649	
N-acetylleucine	0.114	0.295	-0.176	0.155	0.290	0.002	0.152	0.497	0.005	Amino Acid	HMDB0011756	
proline	0.227	0.186	-0.021	0.153	0.248	0.002	0.152			Amino Acid	HMDB0000162,HM	Yes
2-oxoarginine*	0.092	0.579	-0.587	0.408	0.679	0.002	0.152			Amino Acid	HMDB0004225	
glutamate	0.176	0.160	-0.168	0.220	0.344	0.002	0.152	0.495	0.005	Amino Acid	HMDB0000148	
indoleacetate	0.243	0.395	-0.334	0.368	0.577	0.002	0.152			Amino Acid	HMDB0000197	
17alpha-hydroxypregnanolone glucuronide	-0.429	0.775	-1.405	0.609	0.975	0.002	0.152			Lipid	NA	
tryptophan	0.161	0.232	-0.084	0.145	0.245	0.003	0.152			Amino Acid	HMDB0000929	Yes
N,N,N-trimethyl-5-aminovalerate	0.229	0.373	-0.215	0.277	0.444	0.003	0.152	0.428	0.018	Amino Acid	NA	
paraxanthine	0.698	0.891	-0.355	0.809	1.054	0.003	0.152	0.498	0.005	Xenobiotics	HMDB0001860	Yes
azelate (C9-DC)	-0.232	0.472	0.275	0.198	-0.508	0.003	0.152	-0.427	0.019	Lipid	HMDB0000784	
3-methylglutaconate	0.222	0.595	-0.380	0.361	0.602	0.003	0.152			Amino Acid	HMDB0000522	
N-acetylphenylalanine	-0.117	0.386	-0.450	0.173	0.333	0.003	0.152			Amino Acid	HMDB0000512	Yes
N-acetylglutamine	0.086	0.413	-0.280	0.199	0.366	0.003	0.152			Amino Acid	HMDB0006029	
N-acetylhistidine	0.195	0.445	-0.246	0.190	0.441	0.003	0.152	0.561	0.001	Amino Acid	HMDB0032055	
glycochenodeoxycholate	0.992	0.874	-0.308	0.913	1.300	0.004	0.178			Lipid	HMDB0000637	
3,7-dimethylurate	0.433	0.600	-0.687	1.083	1.120	0.005	0.178	0.490	0.006	Xenobiotics	HMDB0001982	
indoleacetylglutamine	0.326	0.806	-0.388	0.428	0.714	0.005	0.178			Amino Acid	HMDB0013240	Yes
beta-hydroxyisovaleroylcarnitine	0.185	0.310	-0.143	0.221	0.329	0.005	0.178	0.524	0.003	Amino Acid	NA	
lyxonate	-0.026	0.214	-0.282	0.178	0.256	0.005	0.178	0.473	0.008	Carbohydrate	HMDB0060255	
1-methylurate	0.640	0.859	-0.214	0.559	0.854	0.005	0.178			Xenobiotics	HMDB0003099	
alanine	0.064	0.170	-0.196	0.192	0.260	0.006	0.197	0.446	0.014	Amino Acid	HMDB0000161	Yes
caffeine	0.031	1.198	-1.327	0.990	1.358	0.007	0.197			Xenobiotics	HMDB0001847	
1-(1-enyl-stearoyl)-2-oleoyl-GPE (P-18:0/18:1)	0.367	0.262	-0.024	0.297	0.391	0.007	0.197			Lipid	HMDB0011375	
N-acetyltryptophan	0.059	0.217	-0.393	0.455	0.452	0.007	0.197	0.396	0.030	Amino Acid	HMDB0013713	Yes
11beta-hydroxyandrosterone glucuronide	-0.028	0.742	-0.733	0.895	0.705	0.007	0.197			Lipid	HMDB0010351	
catechol sulfate	0.889	0.749	-0.025	0.680	0.915	0.007	0.197	0.396	0.030	Xenobiotics	HMDB0059724	Yes
1-(1-enyl-palmitoyl)-2-linoleoyl-GPE (P-16:0/18:2)*	0.799	0.412	0.314	0.359	0.485	0.007	0.197	0.472	0.008	Lipid	HMDB0011343	
3-formylindole	0.082	0.363	-0.280	0.256	0.362	0.007	0.197			Xenobiotics	HMDB29737	
S-carboxyethylcysteine	0.274	0.548	-0.364	0.473	0.639	0.007	0.197			Amino Acid	NA	Yes
2-naphthol sulfate	0.624	1.038	-0.399	0.735	1.023	0.008	0.205			Xenobiotics	NA	Yes
ribonate	0.013	0.341	-0.344	0.263	0.357	0.008	0.205	0.470	0.009	Carbohydrate	HMDB0000867	
N-acetyl-1-methylhistidine*	-0.310	0.806	-1.046	0.514	0.736	0.008	0.205			Amino Acid	HMDB0240340	
N-acetylglutamate	0.050	0.246	-0.311	0.283	0.361	0.008	0.207	0.366	0.046	Amino Acid	HMDB0001138	
7-methylxanthine	0.754	0.662	-0.242	0.791	0.996	0.009	0.217	0.514	0.004	Xenobiotics	HMDB0001991	
gluconate	-0.359	0.460	-0.677	0.165	0.318	0.010	0.224			Xenobiotics	HMDB0000625	
N-acetylproline	0.265	0.520	-0.201	0.337	0.466	0.010	0.224			Amino Acid	HMDB0094701	Yes
theobromine	0.717	0.832	-0.342	0.873	1.060	0.012	0.256	0.519	0.003	Xenobiotics	HMDB0002825	
kynurenine	0.103	0.379	-0.276	0.299	0.379	0.012	0.256			Amino Acid	HMDB0000684	
tetrahydrocortisol glucuronide	-0.411	0.475	-0.898	0.419	0.488	0.012	0.256	0.419	0.021	Lipid	NA	
argininate*	0.183	0.449	-0.342	0.416	0.525	0.012	0.256	0.372	0.043	Amino Acid	HMDB0003148	
4-hydroxy-2-oxoglutaric acid	-0.165	0.349	-0.454	0.213	0.289	0.013	0.263	0.466	0.009	Lipid	HMDB0002070	
3-sulfo-L-alanine	0.223	0.490	-0.299	0.600	0.522	0.013	0.263	0.440	0.015	Amino Acid	HMDB0002757	
1-(1-enyl-palmitoyl)-GPE (P-16:0)*	0.525	0.290	0.199	0.275	0.326	0.014	0.271			Lipid	HMDB0011152	
3-hydroxybutyrate (BHBA)	-0.233	0.703	0.740	0.843	-0.973	0.014	0.271	-0.382	0.037	Lipid	HMDB0000442,HMDB0000357,HMD	
1-(1-enyl-stearoyl)-GPE (P-18:0)*	0.619	0.273	0.279	0.294	0.341	0.015	0.271			Lipid	HMDB0240598	
lactate	-0.096	0.313	-0.397	0.247	0.301	0.015	0.271			Carbohydrate	HMDB0000190	
aspartate	0.155	0.217	-0.114	0.234	0.270	0.015	0.271	0.398	0.029	Amino Acid	HMDB0000191	
3-methoxycatechol sulfate (1)	1.104	1.858	-0.634	1.428	1.738	0.015	0.271			Xenobiotics	NA	
pyridoxate	0.244	0.355	-0.051	0.229	0.294	0.015	0.271	0.396	0.030	Cofactors and Vitamins	HMDB0000017	Yes
guaiacol sulfate	0.767	0.753	-0.069	0.720	0.836	0.016	0.271	0.450	0.012	Xenobiotics	HMDB0060013	
prolylglycine	0.110	0.569	-0.736	0.866	0.846	0.016	0.271	0.403	0.027	Peptide	HMDB0011178	Yes
N-acetylkynurenine (2)	0.419	0.656	-0.254	0.576	0.673	0.016	0.274			Amino Acid	HMDB0240342	Yes
1-(1-enyl-palmitoyl)-2-oleoyl-GPE (P-16:0/18:1)*	0.240	0.279	-0.034	0.233	0.274	0.017	0.274	0.430	0.018	Lipid	HMDB0011342	
glycodeoxycholate	1.180	0.962	-0.124	1.168	1.304	0.017	0.274			Lipid	HMDB00631	
3-indoleglyoxylic acid	0.293	0.417	-0.082	0.312	0.375	0.017	0.274			Xenobiotics	NA	
dihydrocaffeate sulfate (2)	0.491	1.145	-0.516	0.535	1.007	0.017	0.274	0.464	0.010	Xenobiotics	NA	
2-hydroxyglutarate	0.007	0.420	-0.361	0.371	0.369	0.018	0.274	0.362	0.049	Lipid	HMDB0059655	
1-stearoyl-2-oleoyl-GPS (18:0/18:1)	0.113	0.499	-0.380	0.455	0.493	0.018	0.274			Lipid	HMDB0010163	
citraconate/glutaconate	0.059	0.615	-0.516	0.491	0.575	0.018	0.274	0.470	0.009	Energy	HMDB0000634,HMDB0000620	
1,7-dimethylurate	0.483	0.678	-0.330	0.733	0.813	0.018	0.274			Xenobiotics	HMDB0011103	Yes
metabolonic lactone sulfate	0.301	0.693	-0.185	0.348	0.485	0.018	0.274			Partially Characterized Molecules	NA	Yes
theophylline	0.520	0.954	-0.333	1.110	0.853	0.019	0.278			Xenobiotics	HMDB0001889	Yes
sphingadienine	0.231	0.431	-0.158	0.335	0.390	0.019	0.278	0.366	0.047	Lipid	NA	
4-guanidinobutanoate	0.301	0.599	-0.176	0.341	0.477	0.020	0.279			Amino Acid	HMDB0003464	Yes
eugenol sulfate	0.068	1.551	-1.205	1.202	1.273	0.020	0.279			Xenobiotics	HMDB0135245	Yes
2,3-dihydroxyisovalerate	0.437	0.611	-0.107	0.472	0.545	0.020	0.279	0.459	0.011	Xenobiotics	HMDB0012141	
3-methylxanthine	0.673	0.684	-0.303	0.919	0.976	0.021	0.279	0.492	0.006	Xenobiotics	HMDB0001886	
1-ribosyl-imidazoleacetate*	0.247	0.421	-0.185	0.392	0.432	0.021	0.279			Amino Acid	HMDB0002331	
taurocholate	-0.141	1.435	-1.584	1.056	1.443	0.021	0.279	0.459	0.011	Lipid	HMDB0000036	
andro steroid monosulfate C19H28O6S (1)*	0.353	0.973	-0.540	0.793	0.893	0.021	0.279			Lipid	HMDB0002759	
gamma-CEHC glucuronide*	0.196	0.708	-0.408	0.523	0.604	0.022	0.279			Cofactors and Vitamins	NA	
N-acetylasparagine	0.186	0.382	-0.113	0.248	0.299	0.022	0.279			Amino Acid	HMDB0006028	
methyl indole-3-acetate	0.275	0.758	-0.416	0.618	0.692	0.022	0.281			Xenobiotics	HMDB0029738	
N-acetyl-2-aminooctanoate*	0.322	0.574	-0.052	0.268	0.374	0.023	0.283			Lipid	HMDB0059745	
gamma-glutamylalanine	-0.108	0.634	-0.866	0.727	0.758	0.023	0.283			Peptide	HMDB0029142	
1-(1-enyl-stearoyl)-2-linoleoyl-GPE (P-18:0/18:2)*	0.745	0.348	0.253	0.475	0.492	0.023	0.283	0.399	0.029	Lipid	HMDB0011376	
N-oleoyltaurine	-0.999	0.765	-0.116	1.075	-0.883	0.023	0.283			Lipid	NA	
taurochenodeoxycholate	0.503	1.110	-0.652	1.086	1.155	0.024	0.283	0.369	0.045	Lipid	HMDB0000951	
16a-hydroxy DHEA 3-sulfate	0.251	1.003	-0.736	0.918	0.986	0.024	0.283			Lipid	HMDB0062544	
7-methylurate	0.604	0.756	-0.345	0.917	0.949	0.024	0.283	0.364	0.048	Xenobiotics	HMDB0011107	
glucuronide of piperine metabolite C17H21NO3 (4)*	0.634	0.857	-0.440	1.048	1.074	0.025	0.292	0.389	0.034	Xenobiotics	NA	
tyrosine	0.077	0.274	-0.098	0.175	0.175	0.026	0.295			Amino Acid	HMDB0000158	Yes
3-acetylphenol sulfate	-0.179	0.823	-0.734	0.471	0.555	0.026	0.295	0.370	0.044	Xenobiotics	NA	
1-stearoyl-2-oleoyl-GPI (18:0/18:1)*	0.456	0.430	-0.057	0.503	0.513	0.026	0.295			Lipid	HMDB0240667	
lactose	-1.000	0.706	-1.530	0.000	0.530	0.027	0.297	0.551	0.002	Carbohydrate	HMDB0041627	
gamma-glutamylphenylalanine	0.015	0.251	-0.205	0.204	0.220	0.027	0.297			Peptide	HMDB0000594	Yes
1-palmitoyl-GPG (16:0)*	0.288	0.347	-0.027	0.382	0.315	0.027	0.297			Lipid	HMDB0240601	
histidine betaine (hercynine)*	0.164	0.969	0.807	0.511	-0.643	0.028	0.298			Xenobiotics	HMDB0029422	
4-acetamidobutanoate	-0.121	0.311	-0.436	0.310	0.316	0.029	0.306	0.380	0.038	Amino Acid	HMDB0003681	
maltose	-0.005	0.674	-0.561	0.388	0.556	0.029	0.306			Carbohydrate	HMDB0000163	Yes
delta-CEHC	-0.079	0.529	-0.575	0.472	0.496	0.032	0.327			Cofactors and Vitamins	NA	
arachidonate (20:4n6)	0.191	0.317	0.491	0.303	-0.300	0.034	0.346	-0.423	0.020	Lipid	HMDB0001043	
4-ethylphenylsulfate	0.680	0.845	-0.272	0.993	0.952	0.035	0.346	0.413	0.023	Xenobiotics	HMDB0062551	
glyco-beta-muricholate**	0.421	0.959	-0.571	1.025	0.992	0.035	0.346			Lipid	NA	
N-(2-furoyl)glycine	-1.377	0.709	-1.950	0.419	0.572	0.035	0.346	0.401	0.028	Xenobiotics	HMDB0000439	
retinol (Vitamin A)	0.279	0.262	-0.025	0.321	0.305	0.035	0.346	0.374	0.042	Cofactors and Vitamins	HMDB0000305	
3-(3-hydroxyphenyl)propionate	0.302	1.122	-0.683	0.988	0.985	0.035	0.346			Xenobiotics	HMDB0000375	
2-methoxyresorcinol sulfate	-0.411	2.014	-2.091	0.934	1.680	0.036	0.346			Xenobiotics	NA	
7-methylguanine	-0.027	0.213	-0.264	0.251	0.237	0.037	0.353	0.375	0.041	Nucleotide	HMDB0000897	
taurodeoxycholate	0.986	1.154	-0.211	1.270	1.197	0.038	0.362			Lipid	HMDB0000896	
N-acetylisoleucine	0.096	0.239	-0.128	0.235	0.224	0.039	0.362	0.512	0.004	Amino Acid	HMDB0061684	
sphingosine	0.128	0.417	-0.420	0.599	0.548	0.039	0.362	0.374	0.042	Lipid	HMDB0000252	
erythronate*	0.030	0.311	-0.217	0.249	0.247	0.040	0.362	0.441	0.015	Carbohydrate	HMDB0000613	
hexadecasphingosine (d16:1)*	-0.084	0.445	-0.481	0.350	0.396	0.040	0.362			Lipid	NA	
mannitol/sorbitol	-2.955	1.207	-3.710	0.873	0.755	0.041	0.362	0.422	0.020	Carbohydrate	HMDB0000247,HM	Yes
N6-methyllysine	0.058	0.706	0.739	0.691	-0.681	0.041	0.362			Amino Acid	HMDB0002038	Yes
10-heptadecenoate (17:1n7)	0.241	0.537	0.762	0.670	-0.522	0.041	0.362	-0.502	0.005	Lipid	HMDB0060038	
cystathionine	-0.150	0.713	-0.714	0.538	0.564	0.041	0.362			Amino Acid	HMDB0000099	
gamma-glutamylglutamate	0.094	0.335	-0.173	0.272	0.267	0.042	0.362	0.375	0.041	Peptide	HMDB0011737	Yes
palmitoleate (16:1n7)	0.278	0.709	0.846	0.581	-0.568	0.042	0.362	-0.415	0.023	Lipid	HMDB0003229	
acetoacetate	-0.742	0.699	0.083	1.194	-0.824	0.042	0.363			Lipid	HMDB0000060	
androstenediol (3beta,17beta) monosulfate (2)	0.346	0.655	-0.055	0.353	0.401	0.043	0.363			Lipid	HMDB0240429,HMDB0186954	
sucrose	0.164	0.890	-0.481	0.638	0.644	0.043	0.363			Carbohydrate	HMDB0000258	
1-methylxanthine	0.704	0.930	-0.091	0.846	0.795	0.044	0.374			Xenobiotics	HMDB0010738	Yes
biliverdin	-0.460	0.583	-0.097	0.331	-0.362	0.045	0.378			Cofactors and Vitamins	HMDB0001008	Yes
myristate (14:0)	0.271	0.475	0.749	0.626	-0.478	0.046	0.379	-0.572	0.001	Lipid	HMDB0000806	
imidazole propionate	-0.083	0.585	-0.549	0.436	0.467	0.046	0.379			Amino Acid	HMDB0002271	
2-ketocaprylate	0.321	0.495	-0.105	0.462	0.426	0.047	0.379			Amino Acid	HMDB13211	Yes
pantoate	-0.104	0.470	-0.484	0.407	0.381	0.047	0.379	0.372	0.043	Cofactors and Vitamins	HMDB0240389	
N-acetyltaurine	-0.113	0.460	-0.622	0.579	0.509	0.048	0.379			Amino Acid	HMDB0240253	Yes
5-acetylamino-6-amino-3-methyluracil	0.611	0.597	-0.231	0.976	0.843	0.048	0.379			Xenobiotics	HMDB0004400	
5-methylthioadenosine (MTA)	-0.096	0.377	-0.366	0.277	0.270	0.048	0.379	0.508	0.004	Amino Acid	HMDB0001173	
hippurate	0.536	0.733	0.010	0.546	0.526	0.049	0.388			Xenobiotics	HMDB0000714	Yes

Table S4 – Significantly regulated transcripts in myocardial biopsies. Column 'Network' indicates weighted gene correlation networks membership and column 'Pathway' includes information about significant pathways the transcripts participate in. Network column annotates genes to each of four networks in Figure 3C, D. The grey network annotation includes transcripts without network membership.

Ensembl ID	Symbol	Biotype	Description	logFC	Average Expression	t	P.Value	adj.P.Val	B	Network	Pathways
ENSG00000064687	ABCA7	protein_coding	ATP binding cassette subfamily A member 7 [Source:HGNC Symbol;Acc:HGNC:37]	-0.501062162590073	0.712671072117905	-2.02538865988759	0.0477171484579252	0.970999941189693	-4.2540092845644	blue	NA
ENSG00000073734	ABCB11	protein_coding	ATP binding cassette subfamily B member 11 [Source:HGNC Symbol;Acc:HGNC:42]	-0.888164748222521	-2.10364144429803	-2.04371912993182	0.0458080486419073	0.970999941189693	-4.47041025343218	grey	NA
ENSG00000261459	AC002310.4	protein_coding	novel transcript	-1.6428733889649	-3.1426722250153	-2.37720671641696	0.0209686611228955	0.970999941189693	-4.46152525357606	blue	NA
ENSG00000267653	AC002546.1	lncRNA	novel transcript	-0.779520043930601	-1.58924526124415	-2.28576971565297	0.0261655668444128	0.970999941189693	-4.36506363411377	grey	NA
ENSG00000272918	AC005070.3	lncRNA	novel transcript, antisense to MLL5	-0.513174482547089	-1.93996605037947	-2.13449447627635	0.0372984496930559	0.970999941189693	-4.44746678462189	grey	NA
ENSG00000244480	AC005154.2	transcribed_processed_pseudogene	pseudogene similar golgi autoantigen, golgin subfamily a, 8A GOLGA8A	-0.696054689591286	-2.01933965105071	-2.21131847008392	0.0312112169066253	0.970999941189693	-4.428699474654	grey	NA
ENSG00000280571	AC006059.2	protein_coding	novel protein	-1.02364705208425	0.0957773989414769	-2.44326326663815	0.0178106877749377	0.970999941189693	-3.40861244176762	blue	NA
ENSG00000285238	AC006064.6	protein_coding	novel transcript	2.24752604739859	-2.39472838760052	3.67240480865518	0.00054718409793795	0.970999941189693	-3.96709707673582	yellow	NA
ENSG00000213443	AC007068.1	processed_pseudogene	nucleophosmin (nucleolar phosphoprotein B23, numatrin) (NPM1) pseudogene	-1.00250919718183	-1.59321610279301	-2.19846343102171	0.0321644085950627	0.970999941189693	-4.43013044549141	yellow	NA
ENSG00000204929	AC007389.1	lncRNA	novel transcript	-0.798313396555402	-1.9232824065187	-2.72191041216211	0.00868854968219961	0.970999941189693	-4.34764701953154	grey	NA
ENSG00000272054	AC007390.1	lncRNA	novel transcript	-0.473142881650326	-1.27774264558856	-2.44231788986401	0.0178526856584246	0.970999941189693	-4.36741911027322	turquoise	NA
ENSG00000268861	AC008878.3	protein_coding	Rho/Rac guanine nucleotide exchange factor 18 [Source:NCBI gene;Acc:23370]	2.29479321350928	-2.61896901281488	4.37226917028177	5.551558368576E-05	0.948372716103916	-3.94979931475085	turquoise	NA
ENSG00000259972	AC009120.2	lncRNA	novel transcript, antisense to PSMD7	-0.524685193491303	0.334926736417766	-2.18907445770307	0.0328767017586741	0.970999941189693	-4.25876289445511	turquoise	NA
ENSG00000260729	AC009690.1	protein_coding	novel protein	-1.02376837292934	-2.33546164912748	-2.02043681381184	0.0482445230763246	0.970999941189693	-4.49604286820641	turquoise	NA
ENSG00000258311	AC009779.4	protein_coding	novel protein	-0.466214887093621	1.17169228638343	-2.05044550904031	0.0451242724797453	0.970999941189693	-4.2057580317053	turquoise	NA
ENSG00000273184	AC010655.4	lncRNA	novel transcript, readthrough between HILPDA-METTL2B	-1.2285811441343	-3.21867773607656	-2.23977500399757	0.0291893121888738	0.970999941189693	-4.47606930474714	blue	NA
ENSG00000268049	AC012313.2	lncRNA	novel transcript, antisense to ZNF497	0.55583042374953	-1.7027110106484	2.19964323336537	0.0320758710223766	0.970999941189693	-4.42373383127973	turquoise	NA
ENSG00000286449	AC016590.4	lncRNA	novel transcript	-0.52094242076858	2.32062454173124	-2.9560911261932	0.00459026016745041	0.970999941189693	-3.22930267338334	yellow	NA
ENSG00000267169	AC022098.1	lncRNA	novel transcript, antisense to LPHN1	0.797723230146083	-0.807624388129677	2.20021856739573	0.0320327733215837	0.970999941189693	-4.38333314236071	turquoise	NA
ENSG00000245904	AC025164.2	lncRNA	novel transcript, antisense to BTG1	-0.715775640348036	-0.919711289780532	-2.1861628850674	0.0331003889920656	0.970999941189693	-4.38374189038743	grey	NA
ENSG00000280381	AC026362.2	TEC	novel transcript	-0.659998032349578	-1.66565022523126	-2.24727710887372	0.0286759396452864	0.970999941189693	-4.39961046421018	grey	NA
ENSG00000260816	AC027279.1	lncRNA	novel transcript, sense intronic to WWOX	-0.623894118594697	-1.96249846189924	-2.23229752366373	0.0297090691234321	0.970999941189693	-4.44213299833752	grey	NA
ENSG00000267002	AC060780.1	lncRNA	novel transcript	-0.303905676009661	2.00248058123737	-2.43098082339398	0.0183633185725464	0.970999941189693	-3.71913520151446	blue	NA
ENSG00000279912	AC068448.2	TEC	TEC	-0.80250073772027	-0.179099409337156	-2.21567592615814	0.0308938137698597	0.970999941189693	-4.19992325364069	yellow	NA
ENSG00000257337	AC068888.1	lncRNA	novel transcript, antisense to TENC1 & EIF4B	-0.304068928495567	1.26641992517393	-2.26871557822819	0.0272526690262801	0.970999941189693	-4.03226849895005	yellow	NA
ENSG00000287893	AC083864.5	lncRNA	novel transcript	0.663356074457217	-1.10731426698571	2.35835937157975	0.0219573328917075	0.970999941189693	-4.33173106924593	grey	NA
ENSG00000259380	AC087473.1	lncRNA	novel transcript	-0.531528576515084	-0.829817099112433	-2.05374313563619	0.0447922913824195	0.970999941189693	-4.33670095225742	turquoise	NA
ENSG00000262580	AC087741.1	lncRNA	novel transcript, antisense to CARD14	0.981139161541938	-1.86694454240075	2.24122795205414	0.0290892563200439	0.970999941189693	-4.44131241889511	brown	NA
ENSG00000272335	AC093297.2	lncRNA	novel transcript	-0.253162393775262	1.49919045247786	-2.47350132196771	0.0165134418938376	0.970999941189693	-3.94842603616352	brown	NA
ENSG00000261613	AC093525.6	lncRNA	novel transcript, antisense to PDPK1	-0.420051204068691	-0.637981741385683	-2.09414608960834	0.0408936083572825	0.970999941189693	-4.35617474454956	blue	NA
ENSG00000269937	AC093525.7	lncRNA	novel transcript, antisense to PDPK1	-0.476246968792152	0.0402026629977961	-2.3906459055882	0.0202882249632207	0.970999941189693	-4.18948021185262	blue	NA
ENSG00000273756	AC100756.3	transcribed_unprocessed_pseudogene	golgin subfamily A member 6-like protein 6 [Source:NCBI gene;Acc:102723623]	-1.63028700325773	-3.24629090311842	-3.51230034010929	0.00089888637330953	0.970999941189693	-4.18272687582578	grey	NA
ENSG00000279168	AC105052.4	lncRNA	novel transcript, sense overlapping POLR2J2, UPK3BL and RASA4	0.709600620201174	-1.7050435917614	2.49921169519987	0.0154781899860919	0.970999941189693	-4.34326290457164	yellow	NA
ENSG00000252690	AC105339.2	lncRNA	TEC	-0.589311599700408	-1.87565624351677	-2.08094098067212	0.0421340357928812	0.970999941189693	-4.46800538896285	yellow	NA
ENSG00000251216	AC106895.2	lncRNA	novel transcript	0.797151278089895	-1.01819417794103	2.01456025871355	0.048876900963574	0.970999941189693	-4.42870537850677	brown	NA
ENSG00000251023	AC114980.1	lncRNA	novel transcript	-0.523917548658632	-1.73148320011436	-2.00977130913298	0.0493975162512895	0.970999941189693	-4.45577887365444	blue	NA
ENSG00000266340	AC138207.7	lncRNA	novel transcript, antisense RNF135	-0.892269959007379	-0.539232189904374	-3.75443689310653	0.00042251309794626	0.970999941189693	-3.48743656255383	blue	NA
ENSG00000287222	AC234917.3	lncRNA	novel transcript, antisense to PUF60	-0.549002733571293	-1.72454824261405	-2.00570125553377	0.0498437299956012	0.970999941189693	-4.45270306150645	brown	NA
ENSG00000274422	AC245060.5	lncRNA	novel transcript	-0.781017506124042	-1.92150047011889	-2.44909049787836	0.0175537741945535	0.970999941189693	-4.3288306190316	brown	NA
ENSG00000060971	ACAA1	protein_coding	acetyl-CoA acyltransferase 1 [Source:HGNC Symbol;Acc:HGNC:82]	-0.223117628689693	2.80649159305555	-2.29921001163489	0.025336140466474	0.970999941189693	-3.75113211209927	brown	Innate Immune System;Innate Immune System;Neu
ENSG00000196177	ACADSB	protein_coding	acyl-CoA dehydrogenase short/branched chain [Source:HGNC Symbol;Acc:HGNC:91]	-0.185422440221239	5.24460155321112	-2.01607667851827	0.0487130378555062	0.970999941189693	-3.83155222153274	grey	Branched-chain amino acid catabolism
ENSG00000129048	ACKR4	protein_coding	atypical chemokine receptor 4 [Source:HGNC Symbol;Acc:HGNC:1611]	0.474817017957402	1.90105381687657	2.75310565819506	0.00799482615433173	0.970999941189693	-3.67165486309293	grey	Chemokine receptors bind chemokines;Class A/1 (R
ENSG00000100412	ACO2	protein_coding	aconitase 2 [Source:HGNC Symbol;Acc:HGNC:118]	-0.170019917356596	7.02909613955444	-2.76590028233707	0.00772532730418166	0.970999941189693	-2.63351480866909	blue	Citric acid cycle (TCA cycle);Mitochondrial protein in
ENSG00000162390	ACOT11	protein_coding	acyl-CoA thioesterase 11 [Source:HGNC Symbol;Acc:HGNC:18156]	-0.240489485146584	3.08382228716553	-2.11456706354228	0.0390379667114372	0.970999941189693	-3.89071807355052	turquoise	NA
ENSG00000131069	ACSS2	protein_coding	acyl-CoA synthetase short chain family member 2 [Source:HGNC Symbol;Acc:HGNC:15814]	-0.179347178708741	4.6683040933621	-2.24375916905487	0.0289156707776069	0.970999941189693	-3.56194104543816	turquoise	Mitochondrial biogenesis;Mitochondrial biogenesis
ENSG00000184009	ACTG1	protein_coding	actin gamma 1 [Source:HGNC Symbol;Acc:HGNC:144]	0.274176280321343	7.01037134548943	2.37771453572263	0.0209425828758495	0.970999941189693	-3.27074223408224	grey	EPHB-mediated forward signaling;Fcgamma recept
ENSG00000259158	ADAM20P1	transcribed_unprocessed_pseudogene	ADAM metallopeptidase domain 20 pseudogene 1 [Source:HGNC Symbol;Acc:HGNC:20102]	-0.901838128457124	-1.57485147515663	-3.07738761955874	0.00325974984179975	0.970999941189693	-4.20794904126624	brown	NA
ENSG00000078295	ADCY2	protein_coding	adenylate cyclase 2 [Source:HGNC Symbol;Acc:HGNC:233]	1.03809885749026	-0.869535229327538	2.48080497325958	0.0162131749493651	0.970999941189693	-4.28628116501713	turquoise	Anti-inflammatory response favouring Leishmania p
ENSG00000173175	ADCY5	protein_coding	adenylate cyclase 5 [Source:HGNC Symbol;Acc:HGNC:236]	-0.233793182705213	6.05173002640505	-2.89571250348917	0.00542691039748132	0.970999941189693	-2.44562467851284	blue	Anti-inflammatory response favouring Leishmania p
ENSG00000130706	ADRM1	protein_coding	adhesion regulating molecule 1 [Source:HGNC Symbol;Acc:HGNC:15759]	-0.115283980265917	3.83097916389631	-2.05391021501759	0.0447755275283014	0.970999941189693	-3.84602523329285	turquoise	NA
ENSG00000255020	AF131216.3	lncRNA	novel transcript, antisense to TDH	-0.629649568088898	-1.43674677640699	-2.36956259226286	0.021364740863773	0.970999941189693	-4.3691607420813	blue	NA
ENSG00000160216	AGPAT3	protein_coding	1-acylglycerol-3-phosphate O-acyltransferase 3 [Source:HGNC Symbol;Acc:HGNC:326]	-0.179684636305224	4.46696813508472	-2.37786419201378	0.0209349030589823	0.970999941189693	-3.43692467186389	blue	NA
ENSG00000135744	AGT	protein_coding	angiotensinogen [Source:HGNC Symbol;Acc:HGNC:333]	0.192162543913756	5.84842605971976	2.10243651670088	0.0401311955255805	0.970999941189693	-3.70597529661643	blue	Class A/1 (Rhodopsin-like receptors);GPCR ligand b
ENSG00000286169	AHRR	protein_coding	aryl-hydrocarbon receptor repressor [Source:HGNC Symbol;Acc:HGNC:346]	-1.13687496923265	-2.76378430492184	-2.10487331433038	0.0399094689013004	0.970999941189693	-4.47984749646082	turquoise	NA
ENSG00000156709	AIFM1	protein_coding	apoptosis inducing factor mitochondria associated 1 [Source:HGNC Symbol;Acc:HGNC:8768]	-0.265209017404325	4.03863612991815	-2.6042434074758	0.011831629115997	0.970999941189693	-3.11622849201572	grey	NA
ENSG00000117020	AKT3	protein_coding	AKT serine/threonine kinase 3 [Source:HGNC Symbol;Acc:HGNC:393]	0.187610406986739	5.00915255900381	2.65644592450362	0.0103273409905278	0.970999941189693	-2.92093921988424	turquoise	ESR-mediated signaling
ENSG00000284602	AL031432.4	lncRNA	novel transcript	-0.594935781871477	-1.44840442312316	-2.53341788695257	0.01419187451477	0.970999941189693	-4.314422616953	blue	NA
ENSG00000280434	AL031595.3	lncRNA	novel transcript, sense overlapping PARVB	-0.440136810318286	-0.346739949090601	-2.20624893855628	0.0315841040265066	0.970999941189693	-4.28699894858667	turquoise	NA
ENSG00000272356	AL080317.2	lncRNA	novel transcript, antisense to REV3L	-0.460973171375482	-1.14369668058607	-2.20038498570139	0.0320203166045739	0.970999941189693	-4.40937198099448	blue	NA
ENSG00000286787	AL110114.1	lncRNA	novel transcript, antisense to PSMF1	-1.03078785909902	-1.48692511507707	-2.56160346468225	0.0132056536607851	0.970999941189693	-4.23176043575241	turquoise	NA
ENSG00000259132	AL132780.3	protein_coding	novel protein	0.251970162025663	-2.11349278425045	2.04229770808146	0.0459536870884359	0.970999941189693	-4.24314923896917	blue	NA
ENSG00000151303	AL136982.1	lncRNA	novel transcript	-0.982931959470188	-1.99725818260059	-2.49236062769719	0.0157481607544508	0.970999941189693	-4.40131163180337	blue	NA
ENSG00000277687	AL139407.1	lncRNA	novel transcript, sense intronic to CACUL1	-0.656109610196413	-1.85737262846026	-2.64300262030067	0.0106970403636222	0.970999941189693	-4.37826115027074	blue	NA
ENSG00000260917	AL158212.3	lncRNA	novel transcript, overlapping to VTI1A	-0.198034866872959	1.78229663198338	-2.12196926337724	0.0383837110755647	0.970999941189693	-4.07699802181563	blue	NA
ENSG00000229019	AL161457.1	lncRNA	novel transcript	-0.636262925974188	-1.21504464597166	-2.28685302007462	0.0260978314291486	0.970999941189693	-4.40075437251887	blue	NA
ENSG00000259994	AL353796.1	lncRNA	novel transcript, overlapping to MTPAP	-0.404866143555556	-0.866374546230017	-2.04760000382525	0.0454124484016076	0.970999941189693	-4.39141175934567	blue	NA
ENSG00000205740	AL359878.1	lncRNA	novel transcript, antisense to GTPBP4	-0.475382957461746	-1.45818959179314	-2.37568530172169	0.0210469650685321	0.970999941189693	-4.37744467910059	turquoise	NA
ENSG00000260948	AL390195.3	lncRNA	novel transcript, sense overlapping WDR77 & OVGP1	-0.540534343890324	-1.95552766530483	-2.26832996307324	0.0272777061056887	0.970999941189693	-4.43165274672903	turquoise	NA
ENSG00000287277	AL392086.3	lncRNA	novel transcript	-0.540012455855846	0.627104363988664	-2.03013449087104	0.0472163947104403	0.970999941189693	-4.22975027175767	turquoise	NA
ENSG00000286112	AL441992.3	protein_coding	kynurenine aminotransferase 1 [Source:NCBI gene;Acc:883]	-1.26604492570256	-2.38706651569463	-2.23585205929573	0.0294609834876741	0.970999941189693	-4.43338024559564	yellow	NA
ENSG00000256966	AL513165.2	protein_coding	novel transcript	1.54137058608986	-3.00444761508554	2.79616873192028	0.00712073705937852	0.970999941189693	-4.37448152983528	grey	NA
ENSG00000256029	AL590560.2	protein_coding	novel protein	-0.74476299945747	0.277950763049638	-2.48239794649046	0.0161483432826773	0.970999941189693	-4.19368962057711	blue	NA
ENSG00000276128	AL591441.1	transcribed_processed_pseudogene	glucoside xylosyltransferase 1 pseudogene 5	-0.863231379650557	-1.05898849514087	-2.63915319360388	0.0108051070302807	0.970999941189693	-4.26121689931008	blue	NA
ENSG00000261534	AL596244.1	lncRNA	novel transcript, overlapping TTLL11	-0.443115290072536	-0.413308330895273	-2.78071005413322	0.00742383932536337	0.970999941189693	-4.10288448409252	yellow	NA
ENSG00000203739	AL645568.1	lncRNA	novel transcript	-0.418884787384068	-0.397513292002537	-2.17274265260119	0.0341488137376645	0.970999941189693	-4.34509951564784	turquoise	NA
ENSG00000288853	AL645933.5	lncRNA	novel transcript	1.20863711818847	-2.91724219161989	2.28797636477688	0.0260277569522953	0.970999941189693	-4.47539207592802	blue	NA
ENSG00000263020	AL662899.4	protein_coding	novel protein	0.930265949671727	-2.87850180455205	2.45090364301402	0.0174745190457667	0.970999941189693	-4.37319489599811	grey	NA
ENSG00000274628	AL669942.1	transcribed_unprocessed_pseudogene	novel cytochrome P450 family pseudogene	-1.15717979436716	-1.96484727846057	-2.33349857468401	0.0233251052169096	0.970999941189693	-4.36976209975229	brown	NA
ENSG00000112294	ALDH5A1	protein_coding	aldehyde dehydrogenase 5 family member A1 [Source:HGNC Symbol;Acc:HGNC:408]	-0.247217000462391	3.33047137979568	-2.25577387911025	0.0281041640275798	0.970999941189693	-3.68465665789829	turquoise	NA
ENSG00000119711	ALDH6A1	protein_coding	aldehyde dehydrogenase 6 family member A1 [Source:HGNC Symbol;Acc:HGNC:7179]	-0.248213271377941	4.897655692892	-2.1389917596362	0.0369153772154642	0.970999941189693	-3.69309715350714	brown	Branched-chain amino acid catabolism
ENSG00000136383	ALPK3	protein_coding	alpha kinase 3 [Source:HGNC Symbol;Acc:HGNC:17574]	-0.17465641183152	6.03278960162971	-2.0060526357946	0.0498050704880723	0.970999941189693	-3.82741875179732	yellow	NA
ENSG00000139211	AMIGO2	protein_coding	adhesion molecule with Ig like domain 2 [Source:HGNC Symbol;Acc:HGNC:24073]	0.318692786395617	2.24064113871282	2.22736636638955	0.0300562907108201	0.970999941189693	-3.97013598459094	grey	NA
ENSG00000110200	ANAPC15	protein_coding	anaphase promoting complex subunit 15 [Source:HGNC Symbol;Acc:HGNC:24531]	-0.22980762164982	2.45331487230513	-2.31328925194924	0.0244924349584682	0.970999941189693	-3.77143148697516	brown	Cellular Senescence;Senescence-Associated Secre
ENSG00000187151	ANGPTL5	protein_coding	angiopoietin like 5 [Source:HGNC Symbol;Acc:HGNC:19705]	0.967609942102195	-0.10161881191035	3.26117482323265	0.00191267526685133	0.970999941189693	-4.02733142202567	grey	NA
ENSG00000029534	ANK1	protein_coding	ankyrin 1 [Source:HGNC Symbol;Acc:HGNC:492]	-0.508040081304151	3.90959576024488	-2.74354901559487	0.00820173594553003	0.970999941189693	-2.98480887723768	blue	NA
ENSG00000145362	ANK2	protein_coding	ankyrin 2 [Source:HGNC Symbol;Acc:HGNC:493]	-0.147647760060143	7.55328266492325	-2.43237481191227	0.018299831359337	0.970999941189693	-3.17714952879354	grey	NA
ENSG00000151150	ANK3	protein_coding	ankyrin 3 [Source:HGNC Symbol;Acc:HGNC:494]	-0.142830986266538	6.39279697227563	-2.14017493208151	0.0368151699680861	0.970999941189693	-3.63915736217895	grey	NA
ENSG00000154122	ANKH	protein_coding	ANKH inorganic pyrophosphate transport regulator [Source:HGNC Symbol;Acc:HGNC:15492]	-0.143705335514449	5.14053596057045	-2.01805824848989	0.0484996254594118	0.970999941189693	-3.82696147143066	turquoise	NA
ENSG00000001629	ANKIB1	protein_coding	ankyrin repeat and IBR domain containing 1 [Source:HGNC Symbol;Acc:HGNC:22215]	0.176276462367787	4.51313238758741	2.36013722143126	0.0218623237033261	0.970999941189693	-3.43828937495747	brown	NA
ENSG00000101745	ANKRD12	protein_coding	ankyrin repeat domain 12 [Source:HGNC Symbol;Acc:HGNC:29135]	0.305006234706171	5.39495168456618	3.73961438505687	0.00044281292580908	0.970999941189693	-0.9505446423549	blue	NA
ENSG00000163126	ANKRD23	protein_coding	ankyrin repeat domain 23 [Source:HGNC Symbol;Acc:HGNC:24470]	-0.688020925355345	2.97127924896251	-3.30644988350877	0.00167293357946155	0.970999941189693	-2.71165672858621	grey	NA
ENSG00000074855	ANO8	protein_coding	anoctamin 8 [Source:HGNC Symbol;Acc:HGNC:29329]	-0.249046464013839	1.18111719166232	-2.06135738146214	0.044033820938448	0.970999941189693	-4.18834086634727	blue	Post-translational protein phosphorylation;Post-tran
ENSG00000163297	ANTXR2	protein_coding	ANTXR cell adhesion molecule 2 [Source:HGNC Symbol;Acc:HGNC:21732]	0.134798414314099	4.5570368299336	2.0454032494472	0.0456360115413256	0.970999941189693	-3.82916887385746	blue	Infectious disease;Infectious disease;Infectious dise
ENSG00000135046	ANXA1	protein_coding	annexin A1 [Source:HGNC Symbol;Acc:HGNC:533]	0.319890312471411	5.02289216206261	2.36993277530147	0.021345406674652	0.970999941189693	-3.39689984615014	blue	Class A/1 (Rhodopsin-like receptors);GPCR ligand b
ENSG00000164111	ANXA5	protein_coding	annexin A5 [Source:HGNC Symbol;Acc:HGNC:543]	0.155280357070008	6.14298745353481	2.24151753407258	0.0290693508199913	0.970999941189693	-3.49813964407153	turquoise	Hemostasis;Hemostasis;Platelet degranulation ;Pla
ENSG00000283633	AP000547.3	lncRNA	novel transcript	0.853013142813325	-1.15378590607261	3.31747757090372	0.00161899885662039	0.970999941189693	-3.97321666521068	grey	NA
ENSG00000173727	AP000769.1	transcribed_unprocessed_pseudogene	Finkel-Biskis-Reilly murine sarcoma virus (FBR-MuSV) ubiquitously expressed (FAU) pseudogene	-0.543230690634801	-1.03706330348668	-2.34104947681015	0.022901848849859	0.970999941189693	-4.35141275981204	turquoise	NA
ENSG00000267199	AP001029.2	lncRNA	novel transcript, antisense SPIRE1	-1.20501022698011	-2.31001635080226	-2.32235913274319	0.0239622539436722	0.970999941189693	-4.44829049948119	turquoise	NA
ENSG00000255455	AP003486.1	lncRNA	novel transcript	-0.225047373293252	1.58972835566509	-2.11247929780624	0.039224248180132	0.970999941189693	-4.10871340885943	turquoise	NA
ENSG00000286293	AP005901.5	lncRNA	novel transcript	-0.477523394277007	-0.280115789145561	-2.20110537987837	0.0319664429652258	0.970999941189693	-4.26407102073319	blue	NA
ENSG00000126500	AP006333.1	protein_coding	fibronectin leucine rich transmembrane protein 1 [Source:NCBI gene;Acc:23769]	-0.595918531647853	-0.411894959958774	-2.61631534010541	0.0114670063056276	0.970999941189693	-4.1934578094994	turquoise	NA
ENSG00000006125	AP2B1	protein_coding	adaptor related protein complex 2 subunit beta 1 [Source:HGNC Symbol;Acc:HGNC:563]	-0.129392980226609	5.34273393205864	-2.23200196991748	0.0297297739144118	0.970999941189693	-3.54797406908256	blue	Infectious disease
ENSG00000149089	APIP	protein_coding	APAF1 interacting protein [Source:HGNC Symbol;Acc:HGNC:17581]	-0.182469196154631	2.37460483459193	-2.39976900146123	0.0198376627594404	0.970999941189693	-3.82088909742425	turquoise	NA
ENSG00000137074	APTX	protein_coding	aprataxin [Source:HGNC Symbol;Acc:HGNC:15984]	0.241103041839253	2.26901886896931	2.12568890383028	0.038058574753408	0.970999941189693	-3.9942651502417	brown	NA
ENSG00000165272	AQP3	protein_coding	aquaporin 3 (Gill blood group) [Source:HGNC Symbol;Acc:HGNC:636]	0.483506223199473	0.97948846793001	2.5186311692572	0.0147355470295364	0.970999941189693	-3.940221279755579	grey	NA
ENSG00000089820	ARHGAP4	protein_coding	Rho GTPase activating protein 4 [Source:HGNC Symbol;Acc:HGNC:674]	0.421587734404608	1.80519800152669	2.29135409629388	0.0258180591213375	0.970999941189693	-3.90432327616714	yellow	NA
[illegible]	[illegible]	[illegible]	[illegible]	[illegible]	[illegible]	[illegible]	[illegible]	[illegible]	[illegible]	[illegible]	[illegible]

Table S5 – Pathway enrichment analysis. Column 'Network' indicates pathways which were significantly enriched with genes belonging to one of the four networks in Figure 3C. GeneID lists genes significantly different in the analysis that enriched the pathway. GeneRatio – Ratio of genes in the network belonging to the pathway and all recognised genes in the network. BgRatio - Ratio of genes in the pathway and all genes in the reference dataset.

Network	ID	Description	GeneRatio	BgRatio	pvalue	p.adjust	qvalue	geneID	Count
turquoise	R-HSA-6798695	Neutrophil degranulation	23/133	482/10899	1.640124028506E-08	9.16829331934574E-06	8.28694245981726E-06	SERPINB1/CYBA/FTL/DSP/CTSZ/PYCARD/MAN2B1/RAB5C/CD93/GMFG/CAP1/PRCP/GLIPR1/CREG1/GSN/SURF4/S100A11/RAB31/CD14/CLEC12A/LAMP1/HLA-C/TXNDC5	23
turquoise	R-HSA-114608	Platelet degranulation	10/133	129/10899	3.74514156189556E-06	0.000982810958790097	0.000888333038733163	CD9/IGF1/HGF/TIMP1/CAP1/ECM1/LHFPL2/TAGLN2/ANXA5/TMSB4X	10
turquoise	R-HSA-76005	Response to elevated platelet cytosolic Ca^{2+}	10/133	134/10899	5.27447741747816E-06	0.000982810958790097	0.000888333038733163	CD9/IGF1/HGF/TIMP1/CAP1/ECM1/LHFPL2/TAGLN2/ANXA5/TMSB4X	10
turquoise	R-HSA-9658195	Leishmania infection	10/133	164/10899	3.11335976167251E-05	0.00435092026693734	0.00393266496211265	FYN/CYBA/GNAI3/HMOX1/HCK/PYCARD/CASP1/ENTPD1/ADCY5/ACTG1	10
turquoise	R-HSA-76002	Platelet activation, signaling and aggregation	12/133	263/10899	8.7205519226666E-05	0.00974957704954126	0.00881234720606309	CD9/FYN/IGF1/HGF/GNAI3/TIMP1/CAP1/ECM1/LHFPL2/TAGLN2/ANXA5/TMSB4X	12
turquoise	R-HSA-199992	trans-Golgi Network Vesicle Budding	6/133	72/10899	0.000238201443371138	0.0179753490831042	0.0162473732414838	FTL/CTSZ/RAB5C/SORT1/SNX2/TXNDC5	6
turquoise	R-HSA-9660826	Purinergic signaling in leishmaniasis infection	4/133	26/10899	0.000257250076323494	0.0179753490831042	0.0162473732414838	HMOX1/PYCARD/CASP1/ENTPD1	4
turquoise	R-HSA-9664424	Cell recruitment (pro-inflammatory response)	4/133	26/10899	0.000257250076323494	0.0179753490831042	0.0162473732414838	HMOX1/PYCARD/CASP1/ENTPD1	4
turquoise	R-HSA-264870	Caspase-mediated cleavage of cytoskeletal proteins	3/133	12/10899	0.000360596317025821	0.0223970379130482	0.0202440037628531	VIM/GSN/MAPT	3
turquoise	R-HSA-432722	Golgi Associated Vesicle Biogenesis	5/133	56/10899	0.000582074107049791	0.0325379425840833	0.0294100601456736	FTL/RAB5C/SORT1/SNX2/TXNDC5	5
turquoise	R-HSA-844456	The NLRP3 inflammasome	3/133	16/10899	0.000885646032239437	0.045006921092895	0.0406803919114766	HMOX1/PYCARD/CASP1	3
turquoise	R-HSA-1474244	Extracellular matrix organization	11/133	300/10899	0.00109596477458658	0.0487738093413698	0.0440851680328735	DCN/LAMB1/TIMP1/PCOLCE/ASPN/FMOD/P4HA1/SERPINH1/PCOLCE2/BGN/COL14A1	11
turquoise	R-HSA-111465	Apoptotic cleavage of cellular proteins	4/133	38/10899	0.00113427463584581	0.0487738093413698	0.0440851680328735	VIM/DSP/GSN/MAPT	4
blue	R-HSA-1428517	The citric acid (TCA) cycle and respiratory electron transport	15/131	178/10899	3.18924055384E-09	1.50213230085727E-06	1.43012260624695E-06	NDUFS1/SDHA/ATP5F1D/ACO2/OGDH/PDHX/NNT/COQ10A/SUCLA2/NDUFS6/PDP1/NDUFV1/PDHB/ND1/VDAC1	15
blue	R-HSA-71406	Pyruvate metabolism and Citric Acid (TCA) cycle	9/131	55/10899	1.582638604104E-08	3.72711391266482E-06	3.54844234393834E-06	SDHA/ACO2/OGDH/PDHX/NNT/SUCLA2/PDP1/PDHB/VDAC1	9
blue	R-HSA-1268020	Mitochondrial protein import	8/131	64/10899	8.8413333061536E-07	0.000138808932906611	0.000132154666260401	ACO2/CHCHD3/TOMM40/TIMM17A/PMPCA/LDHD/VDAC1/CHCHD10	8
blue	R-HSA-71403	Citric acid cycle (TCA cycle)	5/131	22/10899	5.19292047048868E-06	0.000611466385400042	0.000582153715902152	SDHA/ACO2/OGDH/NNT/SUCLA2	5
blue	R-HSA-70895	Branched-chain amino acid catabolism	4/131	21/10899	0.000101806816036981	0.00879835936486352	0.00837658082340342	ALDH6A1/MCCC2/PPM1K/ACADSB	4
blue	R-HSA-5205685	PINK1-PRKN Mediated Mitophagy	4/131	22/10899	0.000123276570607312	0.00879835936486352	0.00837658082340342	MFN2/TOMM40/MFN1/VDAC1	4
blue	R-HSA-1592230	Mitochondrial biogenesis	7/131	95/10899	0.00014172801227679	0.00879835936486352	0.00837658082340342	ATP5F1D/CHCHD3/PRKAG2/PPARGC1A/ACSS2/PPARGC1B/TMEM11	7
blue	R-HSA-9609507	Protein localization	9/131	163/10899	0.00014944134802316	0.00879835936486352	0.00837658082340342	ACO2/SGTA/CHCHD3/TOMM40/TIMM17A/PMPCA/LDHD/VDAC1/CHCHD10	9
blue	R-HSA-5205647	Mitophagy	4/131	29/10899	0.000375025605223444	0.0196263400066936	0.0186854862953435	MFN2/TOMM40/MFN1/VDAC1	4
blue	R-HSA-389661	Glyoxylate metabolism and glycine degradation	4/131	31/10899	0.000487682422507311	0.0208816746364494	0.0198806422955134	OGDH/PDHX/GOT2/PDHB	4
blue	R-HSA-70268	Pyruvate metabolism	4/131	31/10899	0.000487682422507311	0.0208816746364494	0.0198806422955134	PDHX/PDP1/PDHB/VDAC1	4
blue	R-HSA-71291	Metabolism of amino acids and derivatives	13/131	374/10899	0.000553582472630349	0.0217281120507412	0.0206865029246078	OGDH/PDHX/ALDH6A1/GOT2/CKMT2/MCCC2/RPL3L/APIP/PPM1K/GPT/PDHB/ACADSB/KYAT1	13
blue	R-HSA-163200	Respiratory electron transport, ATP synthesis by chemiosmotic coupling, and heat production by uncoupling proteins.	7/131	127/10899	0.00083461029747128	0.0285127658147383	0.0271459118048464	NDUFS1/SDHA/ATP5F1D/COQ10A/NDUFS6/NDUFV1/ND1	7
blue	R-HSA-204174	Regulation of pyruvate dehydrogenase (PDH) complex	3/131	16/10899	0.00084751320893065	0.0285127658147383	0.0271459118048464	PDHX/PDP1/PDHB	3
blue	R-HSA-611105	Respiratory electron transport	6/131	103/10899	0.00148320021907797	0.0465724868790484	0.0443398802334889	NDUFS1/SDHA/COQ10A/NDUFS6/NDUFV1/ND1	6
yellow	R-HSA-5668599	RHO GTPases Activate NADPH Oxidases	4/39	24/10899	1.41276548131037E-06	0.00026842544144897	0.00020819701829837	NCF4/NCF2/S100A8/S100A9	4
yellow	R-HSA-6798695	Neutrophil degranulation	9/39	482/10899	3.88238190446536E-05	0.0036882628092421	0.00286070245592185	TNFRSF1B/ACAA1/FCN1/LYZ/S100A8/FCGR3B/S100A9/SELL/SERPINA1	9
yellow	R-HSA-1236975	Antigen processing-Cross presentation	4/39	105/10899	0.000516191817265061	0.0326921484267872	0.0253567910235469	NCF4/NCF2/S100A8/S100A9	4
brown	R-HSA-156842	Eukaryotic Translation Elongation	16/69	93/10899	2.72678183762977E-19	1.55426564744897E-16	1.23135727194018E-16	RPLP0/RPL3/EEF1D/RPS19/RPL18A/EEF1B2/RPS15/RPL23/RPS8/RPL7/RPL7A/RPS3/RPL29/RPL13/RPS9/UBA52	16
brown	R-HSA-192823	Viral mRNA Translation	15/69	89/10899	5.55665175779607E-18	1.18582508639971E-15	9.39462533823594E-16	RPLP0/RPL3/DNAJC3/RPS19/RPL18A/RPS15/RPL23/RPS8/RPL7/RPL7A/RPS3/RPL29/RPL13/RPS9/UBA52	15
brown	R-HSA-1799339	SRP-dependent cotranslational protein targeting to membrane	16/69	112/10899	6.24118466526164E-18	1.18582508639971E-15	9.39462533823594E-16	RPLP0/RPL3/RPS19/RPL18A/RPS15/RPL23/RPS8/RPL7/RPL7A/RPS3/RPL29/SSR2/RPL13/RPS9/UBA52/DDOST	16
brown	R-HSA-156902	Peptide chain elongation	14/69	89/10899	2.17857056643002E-16	3.104463E-14	2.459492E-14	RPLP0/RPL3/RPS19/RPL18A/RPS15/RPL23/RPS8/RPL7/RPL7A/RPS3/RPL29/RPL13/RPS9/UBA52	14
brown	R-HSA-2408557	Selenocysteine synthesis	14/69	93/10899	4.15382027847047E-16	3.946129E-14	3.126296E-14	RPLP0/RPL3/RPS19/RPL18A/RPS15/RPL23/RPS8/RPL7/RPL7A/RPS3/RPL29/RPL13/RPS9/UBA52	14
brown	R-HSA-72764	Eukaryotic Translation Termination	14/69	93/10899	4.15382027847047E-16	3.946129E-14	3.126296E-14	RPLP0/RPL3/RPS19/RPL18A/RPS15/RPL23/RPS8/RPL7/RPL7A/RPS3/RPL29/RPL13/RPS9/UBA52	14
brown	R-HSA-975956	Nonsense Mediated Decay (NMD) independent of the Exon Junction Complex (EJC)	14/69	95/10899	5.67018282360618E-16	4.617149E-14	3.657907E-14	RPLP0/RPL3/RPS19/RPL18A/RPS15/RPL23/RPS8/RPL7/RPL7A/RPS3/RPL29/RPL13/RPS9/UBA52	14
brown	R-HSA-72689	Formation of a pool of free 40S subunits	14/69	101/10899	1.3830260311851E-15	8.759165E-14	6.939394E-14	RPLP0/RPL3/RPS19/RPL18A/RPS15/RPL23/RPS8/RPL7/RPL7A/RPS3/RPL29/RPL13/RPS9/UBA52	14
brown	R-HSA-9633012	Response of EIF2AK4 (GCN2) to amino acid deficiency	14/69	101/10899	1.3830260311851E-15	8.759165E-14	6.939394E-14	RPLP0/RPL3/RPS19/RPL18A/RPS15/RPL23/RPS8/RPL7/RPL7A/RPS3/RPL29/RPL13/RPS9/UBA52	14
brown	R-HSA-168273	Influenza Viral RNA Transcription and Replication	15/69	135/10899	3.60872686045589E-15	2.0569743E-13	1.6296251E-13	RPLP0/RPL3/DNAJC3/RPS19/RPL18A/RPS15/RPL23/RPS8/RPL7/RPL7A/RPS3/RPL29/RPL13/RPS9/UBA52	15
brown	R-HSA-156827	L13a-mediated translational silencing of Ceruloplasmin expression	14/69	111/10899	5.40471519003131E-15	2.8006251E-13	2.2187778E-13	RPLP0/RPL3/RPS19/RPL18A/RPS15/RPL23/RPS8/RPL7/RPL7A/RPS3/RPL29/RPL13/RPS9/UBA52	14
brown	R-HSA-72706	GTP hydrolysis and joining of the 60S ribosomal subunit	14/69	112/10899	6.14750509204176E-15	2.9200649E-13	2.3134032E-13	RPLP0/RPL3/RPS19/RPL18A/RPS15/RPL23/RPS8/RPL7/RPL7A/RPS3/RPL29/RPL13/RPS9/UBA52	14
brown	R-HSA-927802	Nonsense-Mediated Decay (NMD)	14/69	115/10899	8.97894026023928E-15	3.6557114E-13	2.8962146E-13	RPLP0/RPL3/RPS19/RPL18A/RPS15/RPL23/RPS8/RPL7/RPL7A/RPS3/RPL29/RPL13/RPS9/UBA52	14
brown	R-HSA-975957	Nonsense Mediated Decay (NMD) enhanced by the Exon Junction Complex (EJC)	14/69	115/10899	8.97894026023928E-15	3.6557114E-13	2.8962146E-13	RPLP0/RPL3/RPS19/RPL18A/RPS15/RPL23/RPS8/RPL7/RPL7A/RPS3/RPL29/RPL13/RPS9/UBA52	14
brown	R-HSA-2408522	Selenoamino acid metabolism	14/69	118/10899	1.29742E-14	4.9067897E-13	3.8873735E-13	RPLP0/RPL3/RPS19/RPL18A/RPS15/RPL23/RPS8/RPL7/RPL7A/RPS3/RPL29/RPL13/RPS9/UBA52	14
brown	R-HSA-72613	Eukaryotic Translation Initiation	14/69	119/10899	1.463428E-14	4.9067897E-13	3.8873735E-13	RPLP0/RPL3/RPS19/RPL18A/RPS15/RPL23/RPS8/RPL7/RPL7A/RPS3/RPL29/RPL13/RPS9/UBA52	14
brown	R-HSA-72737	Cap-dependent Translation Initiation	14/69	119/10899	1.463428E-14	4.9067897E-13	3.8873735E-13	RPLP0/RPL3/RPS19/RPL18A/RPS15/RPL23/RPS8/RPL7/RPL7A/RPS3/RPL29/RPL13/RPS9/UBA52	14
brown	R-HSA-168255	Influenza Infection	15/69	156/10899	3.199458E-14	1.01316182E-12	8.0267114E-13	RPLP0/RPL3/DNAJC3/RPS19/RPL18A/RPS15/RPL23/RPS8/RPL7/RPL7A/RPS3/RPL29/RPL13/RPS9/UBA52	15
brown	R-HSA-72766	Translation	18/69	291/10899	1.423735E-13	4.27120492E-12	3.38383548E-12	RPLP0/RPL3/EEF1D/RPS19/RPL18A/EEF1B2/RPS15/RPL23/RPS8/RPL7/RPL7A/RPS3/RPL29/SSR2/RPL13/RPS9/UBA52/DDOST	18
brown	R-HSA-6791226	Major pathway of rRNA processing in the nucleolus and cytosol	15/69	184/10899	3.7187034E-13	1.059830468E-11	8.39644083E-12	RPLP0/RPL3/RPS19/RPL18A/RPS15/RPL23/RPS8/RPL7/RPL7A/RPS3/RPL29/BMS1/RPL13/RPS9/UBA52	15
brown	R-HSA-9711097	Cellular response to starvation	14/69	156/10899	6.5969783E-13	1.790608397E-11	1.418598342E-11	RPLP0/RPL3/RPS19/RPL18A/RPS15/RPL23/RPS8/RPL7/RPL7A/RPS3/RPL29/RPL13/RPS9/UBA52	14
brown	R-HSA-8868773	rRNA processing in the nucleus and cytosol	15/69	194/10899	8.0934286E-13	2.096933785E-11	1.661282722E-11	RPLP0/RPL3/RPS19/RPL18A/RPS15/RPL23/RPS8/RPL7/RPL7A/RPS3/RPL29/BMS1/RPL13/RPS9/UBA52	15
brown	R-HSA-72312	rRNA processing	15/69	204/10899	1.68722903E-12	4.181393678E-11	3.31268308E-11	RPLP0/RPL3/RPS19/RPL18A/RPS15/RPL23/RPS8/RPL7/RPL7A/RPS3/RPL29/BMS1/RPL13/RPS9/UBA52	15
brown	R-HSA-9010553	Regulation of expression of SLITs and ROBOs	14/69	171/10899	2.34415032E-12	5.567357005E-11	4.410703888E-11	RPLP0/RPL3/RPS19/RPL18A/RPS15/RPL23/RPS8/RPL7/RPL7A/RPS3/RPL29/RPL13/RPS9/UBA52	14
brown	R-HSA-376176	Signaling by ROBO receptors	14/69	218/10899	6.325889296E-11	1.44230275942E-09	1.14265537173E-09	RPLP0/RPL3/RPS19/RPL18A/RPS15/RPL23/RPS8/RPL7/RPL7A/RPS3/RPL29/RPL13/RPS9/UBA52	14
brown	R-HSA-71291	Metabolism of amino acids and derivatives	14/69	374/10899	6.840610869407E-08	1.49967238290845E-06	1.18810609837068E-06	RPLP0/RPL3/RPS19/RPL18A/RPS15/RPL23/RPS8/RPL7/RPL7A/RPS3/RPL29/RPL13/RPS9/UBA52	14
brown	R-HSA-9692914	SARS-CoV-1-host interactions	7/69	95/10899	2.12364951735326E-06	4.48326009219021E-05	3.55183486528088E-05	RPS19/RPS15/RPS8/RPS3/PPIB/RPS9/UBA52	7
brown	R-HSA-9735869	SARS-CoV-1 modulates host translation machinery	5/69	36/10899	2.84235320381882E-06	5.78621902205975E-05	4.58409595653487E-05	RPS19/RPS15/RPS8/RPS3/RPS9	5
brown	R-HSA-9754678	SARS-CoV-2 modulates host translation machinery	5/69	50/10899	1.49163207429151E-05	0.000293182855981435	0.000232272290334322	RPS19/RPS15/RPS8/RPS3/RPS9	5
brown	R-HSA-72695	Formation of the ternary complex, and subsequently, the 43S complex	5/69	51/10899	1.64569378917175E-05	0.000312681819942633	0.000247720223001643	RPS19/RPS15/RPS8/RPS3/RPS9	5
brown	R-HSA-9678108	SARS-CoV-1 Infection	7/69	141/10899	2.88672016797829E-05	0.00053078403088633	0.000420510340258976	RPS19/RPS15/RPS8/RPS3/PPIB/RPS9/UBA52	7
brown	R-HSA-72649	Translation initiation complex formation	5/69	58/10899	3.1020589364159E-05	0.000535810179926384	0.000424492275509545	RPS19/RPS15/RPS8/RPS3/RPS9	5
brown	R-HSA-72702	Ribosomal scanning and start codon recognition	5/69	58/10899	3.1020589364159E-05	0.000535810179926384	0.000424492275509545	RPS19/RPS15/RPS8/RPS3/RPS9	5
brown	R-HSA-72662	Activation of the mRNA upon binding of the cap-binding complex and eIFs, and subsequent binding to 43S	5/69	59/10899	3.37275259348807E-05	0.000565432052437706	0.000447960019382781	RPS19/RPS15/RPS8/RPS3/RPS9	5
brown	R-HSA-9679506	SARS-CoV Infections	11/69	413/10899	5.08646753571691E-05	0.000828367570102469	0.000656269044457912	ANO8/RPS19/RPS15/RPS8/RPS3/TAB3/PPIB/RPS9/IMPDH2/UBA52/DDOST	11
brown	R-HSA-5689901	Metalloprotease DUBs	4/69	37/10899	8.30006293171111E-05	0.00131417663085426	0.00104114824494271	UBA52/H2AC12/H2AC8/H2AC17	4
brown	R-HSA-9694516	SARS-CoV-2 Infection	9/69	298/10899	0.00010158885944936	0.00156501756449014	0.00123987541120271	ANO8/RPS19/RPS15/RPS8/RPS3/TAB3/RPS9/UBA52/DDOST	9
brown	R-HSA-9609690	HCMV Early Events	6/69	135/10899	0.000204265883175031	0.00306398824762546	0.00242742559230161	H2BC11/DYNC1H1/H2AC12/H2AC8/H2BC10/H2AC17	6
brown	R-HSA-73772	RNA Polymerase I Promoter Escape	5/69	91/10899	0.000267963920238483	0.00391639575733167	0.00310274012907717	POLR1A/CBX3/H2BC11/H2AC8/H2BC10	5
brown	R-HSA-9705683	SARS-CoV-2-host interactions	7/69	202/10899	0.000276209242227164	0.00393598170173708	0.00311825697145929	RPS19/RPS15/RPS8/RPS3/TAB3/RPS9/UBA52	7
brown	R-HSA-3214815	HDACs deacetylate histones	5/69	94/10899	0.000311693680200621	0.00433330238327692	0.00343303180503379	H2BC11/H2AC12/H2AC8/H2BC10/H2AC17	5
brown	R-HSA-5689603	UCH proteinases	5/69	102/10899	0.000454871799126152	0.00617326013099778	0.00489072686278494	ASXL2/UBA52/H2AC12/H2AC8/H2AC17	5
brown	R-HSA-9609646	HCMV Infection	6/69	160/10899	0.000509262851054696	0.00675069360700411	0.00534819493518885	H2BC11/DYNC1H1/H2AC12/H2AC8/H2BC10/H2AC17	6
brown	R-HSA-5250913	Positive epigenetic regulation of rRNA expression	5/69	106/10899	0.000542758439823555	0.00703118887953242	0.00557041556661017	POLR1A/CBX3/H2BC11/H2AC8/H2BC10	5
brown	R-HSA-73854	RNA Polymerase I Promoter Clearance	5/69	110/10899	0.000642851322697063	0.00814278342082946	0.00645106941373193	POLR1A/CBX3/H2BC11/H2AC8/H2BC10	5
brown	R-HSA-73864	RNA Polymerase I Transcription	5/69	112/10899	0.000697801006605921	0.00864666464707337	0.00685026617468971	POLR1A/CBX3/H2BC11/H2AC8/H2BC10	5
brown	R-HSA-9610379	HCMV Late Events	5/69	116/10899	0.000818175191070432	0.00992255018957758	0.00786107854354346	H2BC11/H2AC12/H2AC8/H2BC10/H2AC17	5
brown	R-HSA-5357956	TNFR1-induced NFkappaB signaling pathway	3/69	30/10899	0.000872347783759872	0.0103591299321485	0.00820695612353037	TAB3/RACK1/UBA52	3
brown	R-HSA-68616	Assembly of the ORC complex at the origin of replication	4/69	69/10899	0.000933123319118197	0.0108546998346402	0.0085995682900474	ORC6/H2BC11/H2AC8/H2BC10	4
brown	R-HSA-427389	ERCC6 (CSB) and EHMT2 (G9a) positively regulate rRNA expression	4/69	76/10899	0.00133961160070754	0.0152715722480659	0.0120988079306007	CBX3/H2BC11/H2AC8/H2BC10	4
brown	R-HSA-937042	IRAK2 mediated activation of TAK1 complex	2/69	10/10899	0.00172023912964999	0.0192262020372646	0.0152318387331237	TAB3/UBA52	2
brown	R-HSA-3214847	HATs acetylate histones	5/69	142/10899	0.00201423107094336	0.021700699933754	0.0171922442688466	H2BC11/H2AC12/H2AC8/H2BC10/H2AC17	5
brown	R-HSA-68867	Assembly of the pre-replicative complex	5/69	143/10899	0.00207709742508439	0.021700699933754	0.0171922442688466	ORC6/H2BC11/UBA52/H2AC8/H2BC10	5
brown	R-HSA-9014325	TICAM1,TRAF6-dependent induction of TAK1 complex	2/69	11/10899	0.0020939271865903	0.021700699933754	0.0171922442688466	TAB3/UBA52	2
brown	R-HSA-9645460	Alpha-protein kinase 1 signaling pathway	2/69	11/10899	0.0020939271865903	0.021700699933754	0.0171922442688466	TAB3/UBA52	2
brown	R-HSA-212165	Epigenetic regulation of gene expression	5/69	149/10899	0.00248487467929481	0.0252924744142508	0.0200378052146142	POLR1A/CBX3/H2BC11/H2AC8/H2BC10	5
brown	R-HSA-5250924	B-WICH complex positively regulates rRNA expression	4/69	91/10899	0.00259938706170824	0.0258166930179588	0.0204531141361114	POLR1A/H2BC11/H2AC8/H2BC10	4
brown	R-HSA-5689880	Ub-specific processing proteases	6/69	220/10899	0.00262696174568704	0.0258166930179588	0.0204531141361114	H2BC11/UBA52/H2AC12/H2AC8/H2BC10/H2AC17	6
brown	R-HSA-5688426	Deubiquitination	7/69	298/10899	0.00269430953620622	0.0260297700955516	0.0206219231227916	H2BC11/ASXL2/UBA52/H2AC12/H2AC8/H2BC10/H2AC17	7
brown	R-HSA-5625740	RHO GTPases activate PKNs	4/69	95/10899	0.00303826416206642	0.028863509539631	0.0228669355355526	MYL6/H2BC11/H2AC8/H2BC10	4
brown	R-HSA-6798695	Neutrophil degranulation	9/69	482/10899	0.00320685661403236	0.0299657093442368	0.0237401464610851	DNAJC3/MANBA/NPC2/YPEL5/ARPC5/IMPDH2/DYNC1H1/MIF/DDOST	9
brown	R-HSA-69002	DNA Replication Pre-Initiation	5/69	160/10899	0.00337939689360509	0.0310686488605629	0.0246139434186177	ORC6/H2BC11/UBA52/H2AC8/H2BC10	5
brown	R-HSA-75893	TNF signaling	3/69	49/10899	0.00363327773031637	0.0328725127981005	0.0260430433802126	TAB3/RACK1/UBA52	3
brown	R-HSA-2032785	YAP1- and WWTR1 (TAZ)-stimulated gene expression	2/69	15/10899	0.0039326614197637	0.0346745146923698	0.0274706681496337	TEAD3/HIPK2	2
brown	R-HSA-8878171	Transcriptional regulation by RUNX1	6/69	239/10899	0.00395411132456849	0.0346745146923698	0.0274706681496337	HIPK2/CCND2/H2BC11/UBA52/H2AC8/H2BC10	6
brown	R-HSA-171306	Packaging Of Telomere Ends	3/69	52/10899	0.00429973516140868	0.0371340763939841	0.0294192405780594	H2BC11/H2AC8/H2BC10	3
brown	R-HSA-975163	IRAK2 mediated activation of TAK1 complex upon TLR7/8 or 9 stimulation	2/69	16/10899	0.00447615501548026	0.0376932102353777	0.0298622108789973	TAB3/UBA52	2
brown	R-HSA-195258	RHO GTPase Effectors	7/69	327/10899	0.00449673385264155	0.0376932102353777	0.0298622108789973	MYL6/H2BC11/CLIP1/ARPC5/DYNC1H1/H2AC8/H2BC10	7
brown	R-HSA-427413	NoRC negatively regulates rRNA expression	4/69	107/10899	0.00465384917082013	0.0384448409763402	0.0304576856488457	POLR1A/H2BC11/H2AC8/H2BC10	4
brown	R-HSA-937072	TRAF6-mediated induction of TAK1 complex within TLR4 complex	2/69	17/10899	0.00505231249473123	0.040279916237676	0.0319115125871893	TAB3/UBA52	2
brown	R-HSA-5250941	Negative epigenetic regulation of rRNA expression	4/69	110/10899	0.00513313906070875	0.040279916237676	0.0319115125871893	POLR1A/H2BC11/H2AC8/H2BC10	4
brown	R-HSA-110330	Recognition and association of DNA glycosylase with site containing an affected purine	3/69	56/10899	0.00529699181899378	0.040279916237676	0.0319115125871893	H2BC11/H2AC8/H2BC10	3
brown	R-HSA-110331	Cleavage of the damaged purine	3/69	56/10899	0.00529699181899378	0.040279916237676	0.0319115125871893	H2BC11/H2AC8/H2BC10	3
brown	R-HSA-73927	Depurination	3/69	56/10899	0.00529699181899378	0.040279916237676	0.0319115125871893	H2BC11/H2AC8/H2BC10	3
brown	R-HSA-977225	Amyloid fiber formation	4/69	111/10899	0.00529998897864157	0.040279916237676	0.0319115125871893	H2BC11/UBA52/H2AC8/H2BC10	4
brown	R-HSA-2559582	Senescence-Associated Secretory Phenotype (SASP)	4/69	112/10899	0.00547044038501326	0.0410283028875994	0.0325044172461314	H2BC11/UBA52/H2AC8/H2BC10	4
brown	R-HSA-8866654	E3 ubiquitin ligases ubiquitinate target proteins	3/69	59/10899	0.00612893365707418	0.0453700283705491	0.0359441221993824	H2BC11/UBA52/H2BC10	3
brown	R-HSA-69306	DNA Replication	5/69	188/10899	0.00667198915077733	0.0473208272902225	0.0374896304847746	ORC6/H2BC11/UBA52/H2AC8/H2BC10	5
brown	R-HSA-110328	Recognition and association of DNA glycosylase with site containing an affected pyrimidine	3/69	61/10899	0.00672453861492635	0.0473208272902225	0.0374896304847746	H2BC11/H2AC8/H2BC10	3
brown	R-HSA-110329	Cleavage of the damaged pyrimidine	3/69	61/10899	0.00672453861492635	0.0473208272902225	0.0374896304847746	H2BC11/H2AC8/H2BC10	3
brown	R-HSA-73928	Depyrimidination	3/69	61/10899	0.00672453861492635	0.0473208272902225	0.0374896304847746	H2BC11/H2AC8/H2BC10	3

Table S6 – Gene set enrichment analysis. Column 'Comorbidities' lists comorbidities where the pathways were also significant. Green indicates pathways specific for multimorbidity.

Pathway	NGenes	Direction	PValue	FDR	Comorbidities
Classical antibody-mediated complement activation	46	Up	1.201E-28	2.905E-25	Anaemia;Angina/MI;CPD;Diabetes;Extracard_arteriopathy;Heart_failure;Hyperlipidemia;Hypertension;Multimorbid;Obese;
Creation of C4 and C2 activators	51	Up	5.086E-28	6.149E-25	Anaemia;Angina/MI;CPD;Diabetes;Extracard_arteriopathy;Heart_failure;Hyperlipidemia;Hypertension;Multimorbid;Obese;
CD22 mediated BCR regulation	45	Up	2.783E-27	2.243E-24	Anaemia;Angina/MI;CPD;Diabetes;Extracard_arteriopathy;Heart_failure;Hyperlipidemia;Hypertension;Multimorbid;Obese;
FCGR activation	53	Up	2.750E-26	1.559E-23	Anaemia;Angina/MI;CPD;Diabetes;Extracard_arteriopathy;Heart_failure;Hyperlipidemia;Hypertension;Multimorbid;Obese;
Scavenging of heme from plasma	50	Up	3.225E-26	1.559E-23	Anaemia;Angina/MI;CPD;Diabetes;Extracard_arteriopathy;Heart_failure;Hyperlipidemia;Hypertension;Multimorbid;Obese;
Initial triggering of complement	58	Up	1.486E-25	5.987E-23	Anaemia;Angina/MI;CPD;Diabetes;Extracard_arteriopathy;Heart_failure;Hyperlipidemia;Hypertension;Multimorbid;Renal_Disease
Regulation of Complement cascade	71	Up	3.498E-22	1.208E-19	Anaemia;Angina/MI;CPD;Diabetes;Extracard_arteriopathy;Hyperlipidemia;Hypertension;Multimorbid;Renal_Disease
Complement cascade	77	Up	4.558E-22	1.378E-19	Anaemia;Angina/MI;CPD;Diabetes;Extracard_arteriopathy;Hyperlipidemia;Hypertension;Multimorbid;Renal_Disease
Role of LAT2/NTAL/LAB on calcium mobilization	54	Up	4.085E-20	1.098E-17	Anaemia;Angina/MI;CPD;Diabetes;Extracard_arteriopathy;Heart_failure;Hyperlipidemia;Hypertension;Multimorbid;Obese;
Binding and Uptake of Ligands by Scavenger Receptors	78	Up	4.920E-20	1.190E-17	Anaemia;Angina/MI;CPD;Diabetes;Extracard_arteriopathy;Hyperlipidemia;Hypertension;Multimorbid;Obese;Renal_Disease
Antigen activates B Cell Receptor (BCR) leading to generation of second messengers	68	Up	1.911E-18	4.201E-16	Anaemia;Angina/MI;CPD;Diabetes;Extracard_arteriopathy;Hyperlipidemia;Hypertension;Multimorbid;Obese;Renal_Disease
Role of phospholipids in phagocytosis	64	Up	2.907E-17	5.857E-15	Anaemia;Angina/MI;CPD;Diabetes;Extracard_arteriopathy;Heart_failure;Hyperlipidemia;Hypertension;Multimorbid;Obese;
Eukaryotic Translation Elongation	92	Up	2.543E-16	4.730E-14	AnaemiaDiabetes;Extracard_arteriopathy;Hyperlipidemia;Multimorbid;Renal_Disease
Peptide chain elongation	88	Up	3.271E-16	5.650E-14	AnaemiaDiabetes;Extracard_arteriopathy;Hyperlipidemia;Multimorbid;Renal_Disease
FCGR3A-mediated IL10 synthesis	77	Up	7.539E-16	1.139E-13	Anaemia;Angina/MI;CPD;Diabetes;Extracard_arteriopathy;Hyperlipidemia;Hypertension;Multimorbid;Obese;Renal_Disease
Viral mRNA Translation	88	Up	7.386E-16	1.139E-13	AnaemiaDiabetes;Extracard_arteriopathy;Hyperlipidemia;Multimorbid;Renal_Disease
Selenocysteine synthesis	92	Up	3.972E-15	5.650E-13	AnaemiaDiabetes;Extracard_arteriopathy;Hyperlipidemia;Multimorbid;Renal_Disease
FCERI mediated Ca+2 mobilization	69	Up	5.236E-15	7.034E-13	Anaemia;Angina/MI;CPD;Diabetes;Extracard_arteriopathy;Hyperlipidemia;Hypertension;Multimorbid;Obese;Renal_Disease
FCERI mediated MAPK activation	70	Up	6.142E-15	7.816E-13	Anaemia;CPD;Diabetes;Extracard_arteriopathy;Hyperlipidemia;Hypertension;Multimorbid;
Eukaryotic Translation Termination	92	Up	2.381E-14	2.827E-12	AnaemiaDiabetes;Extracard_arteriopathy;Hyperlipidemia;Multimorbid;Renal_Disease
Formation of a pool of free 40S subunits	100	Up	2.455E-14	2.827E-12	AnaemiaDiabetes;Extracard_arteriopathy;Hyperlipidemia;Multimorbid;Renal_Disease
SRP-dependent cotranslational protein targeting to membrane	111	Up	2.753E-14	3.026E-12	AnaemiaDiabetes;Extracard_arteriopathy;Hyperlipidemia;Multimorbid;Renal_Disease
Response of EIF2AK4 (GCN2) to amino acid deficiency	100	Up	4.309E-14	4.530E-12	AnaemiaDiabetes;Extracard_arteriopathy;Hyperlipidemia;Multimorbid;Renal_Disease
Immunoregulatory interactions between a Lymphoid and a non-Lymphoid cell	130	Up	7.895E-14	7.954E-12	Anaemia;Angina/MI;CPD;Diabetes;Extracard_arteriopathy;Hyperlipidemia;Hypertension;Multimorbid;Renal_Disease
L13a-mediated translational silencing of Ceruloplasmin expression	110	Up	8.491E-14	8.213E-12	AnaemiaDiabetes;Extracard_arteriopathy;Hyperlipidemia;Multimorbid;Renal_Disease
FCGR3A-mediated phagocytosis	99	Up	1.459E-13	1.260E-11	Anaemia;CPD;Diabetes;Extracard_arteriopathy;Hyperlipidemia;Hypertension;Multimorbid;
Leishmania phagocytosis	99	Up	1.459E-13	1.260E-11	Anaemia;CPD;Diabetes;Extracard_arteriopathy;Hyperlipidemia;Hypertension;Multimorbid;
Parasite infection	99	Up	1.459E-13	1.260E-11	Anaemia;CPD;Diabetes;Extracard_arteriopathy;Hyperlipidemia;Hypertension;Multimorbid;
GTP hydrolysis and joining of the 60S ribosomal subunit	111	Up	2.992E-13	2.495E-11	AnaemiaDiabetes;Extracard_arteriopathy;Hyperlipidemia;Multimorbid;Renal_Disease
Nonsense Mediated Decay (NMD) independent of the Exon Junction Complex (EJC)	94	Up	3.278E-13	2.642E-11	AnaemiaDiabetes;Extracard_arteriopathy;Hyperlipidemia;Multimorbid;Renal_Disease
Cap-dependent Translation Initiation	118	Up	3.092E-12	2.336E-10	AnaemiaDiabetes;Extracard_arteriopathy;Hyperlipidemia;Multimorbid;Renal_Disease
Eukaryotic Translation Initiation	118	Up	3.092E-12	2.336E-10	AnaemiaDiabetes;Extracard_arteriopathy;Hyperlipidemia;Multimorbid;Renal_Disease
Regulation of actin dynamics for phagocytic cup formation	101	Up	7.020E-12	5.143E-10	Anaemia;CPD;Diabetes;Extracard_arteriopathy;Hyperlipidemia;Hypertension;Multimorbid;
Anti-inflammatory response favouring Leishmania parasite infection	141	Up	3.175E-11	2.194E-09	Anaemia;CPD;Diabetes;Extracard_arteriopathyHypertension;Multimorbid;
Leishmania parasite growth and survival	141	Up	3.175E-11	2.194E-09	Anaemia;CPD;Diabetes;Extracard_arteriopathyHypertension;Multimorbid;
RNA Polymerase I Promoter Opening	52	Up	9.145E-11	6.143E-09	Angina/MIDiabetes;Extracard_arteriopathyHypertension;Multimorbid;
The citric acid (TCA) cycle and respiratory electron transport	170	Down	1.260E-10	8.236E-09	CPD;Stroke;DiabetesHyperlipidemia;Hypertension;Multimorbid;Renal_Disease
Amyloid fiber formation	86	Up	2.114E-10	1.345E-08	Angina/MIDiabetes;Extracard_arteriopathyHypertension;Multimorbid;
Selenoamino acid metabolism	114	Up	4.106E-10	2.546E-08	AnaemiaDiabetes;Extracard_arteriopathy;Hyperlipidemia;Multimorbid;Renal_Disease
DNA methylation	53	Up	6.689E-10	4.044E-08	Diabetes;Extracard_arteriopathyHypertension;Multimorbid;
Fcgamma receptor (FCGR) dependent phagocytosis	124	Up	8.039E-10	4.741E-08	Anaemia;CPD;Diabetes;Extracard_arteriopathy;Hyperlipidemia;Hypertension;Multimorbid;
Activated PKN1 stimulates transcription of AR (androgen receptor) regulated genes KLK2 and KLK3	54	Up	8.884E-10	5.114E-08	Diabetes;Extracard_arteriopathyHypertension;Multimorbid;
SIRT1 negatively regulates rRNA expression	57	Up	1.283E-09	7.212E-08	Diabetes;Extracard_arteriopathyMultimorbid;
Cell surface interactions at the vascular wall	144	Up	1.806E-09	9.569E-08	Anaemia;CPD;Diabetes;Extracard_arteriopathy;Hyperlipidemia;Hypertension;Multimorbid;
Nonsense Mediated Decay (NMD) enhanced by the Exon Junction Complex (EJC)	114	Up	1.820E-09	9.569E-08	AnaemiaDiabetes;Extracard_arteriopathy;Hyperlipidemia;Multimorbid;Renal_Disease
Nonsense-Mediated Decay (NMD)	114	Up	1.820E-09	9.569E-08	AnaemiaDiabetes;Extracard_arteriopathy;Hyperlipidemia;Multimorbid;Renal_Disease
Respiratory electron transport, ATP synthesis by chemiosmotic coupling, and heat production by uncoupling proteins.	124	Down	2.185E-09	1.124E-07	CPD;Stroke;DiabetesHyperlipidemia;Hypertension;Multimorbid;Renal_Disease
Citric acid cycle (TCA cycle)	22	Down	3.962E-09	1.996E-07	CPD;DiabetesHypertension;Multimorbid;Renal_Disease
Influenza Viral RNA Transcription and Replication	135	Up	5.162E-09	2.547E-07	AnaemiaDiabetes;Extracard_arteriopathy;Hyperlipidemia;Multimorbid;Renal_Disease
Respiratory electron transport	101	Down	1.407E-08	6.804E-07	CPD;Stroke;DiabetesHyperlipidemia;Hypertension;Multimorbid;Renal_Disease
PRC2 methylates histones and DNA	62	Up	1.762E-08	8.353E-07	Extracard_arteriopathyHypertension;Multimorbid;
Condensation of Prophase Chromosomes	62	Up	2.346E-08	1.091E-06	Extracard_arteriopathyMultimorbid;
ERCC6 (CSB) and EHMT2 (G9a) positively regulate rRNA expression	65	Up	2.567E-08	1.171E-06	Extracard_arteriopathyHypertension;Multimorbid;
Transcriptional regulation of granulopoiesis	77	Up	3.774E-08	1.690E-06	Diabetes;Extracard_arteriopathyHypertension;Multimorbid;
Leishmania infection	220	Up	4.708E-08	2.070E-06	Anaemia;CPD;Diabetes;Extracard_arteriopathy;Hyperlipidemia;Hypertension;Multimorbid;
Senescence-Associated Secretory Phenotype (SASP)	97	Up	7.568E-08	3.268E-06	Diabetes;Extracard_arteriopathyMultimorbid;
Interleukin-4 and Interleukin-13 signaling	85	Up	8.200E-08	3.479E-06	CPD;Stroke;DiabetesHyperlipidemia;Multimorbid;
Packaging Of Telomere Ends	43	Up	1.075E-07	4.481E-06	Extracard_arteriopathyMultimorbid;
Formation of the ternary complex, and subsequently, the 43S complex	51	Up	1.577E-07	6.463E-06	AnaemiaExtracard_arteriopathy;Hyperlipidemia;Multimorbid;Renal_Disease
Cleavage of the damaged purine	47	Up	1.995E-07	7.781E-06	Extracard_arteriopathyMultimorbid;
Depurination	47	Up	1.995E-07	7.781E-06	Extracard_arteriopathyMultimorbid;
Recognition and association of DNA glycosylase with site containing an affected purine	47	Up	1.995E-07	7.781E-06	Extracard_arteriopathyMultimorbid;
Activation of the mRNA upon binding of the cap-binding complex and eIFs, and subsequent binding to 43S	59	Up	2.199E-07	8.441E-06	AnaemiaExtracard_arteriopathy;Hyperlipidemia;Multimorbid;
RHO GTPases activate PKNs	79	Up	2.499E-07	9.443E-06	Multimorbid;
Translation initiation complex formation	58	Up	2.600E-07	9.670E-06	AnaemiaExtracard_arteriopathy;Hyperlipidemia;Multimorbid;
Peptide ligand-binding receptors	85	Up	3.173E-07	1.162E-05	Diabetes;Extracard_arteriopathy;Hyperlipidemia;Multimorbid;
Chemokine receptors bind chemokines	36	Up	4.384E-07	1.559E-05	AnaemiaDiabetes;Extracard_arteriopathy;Hyperlipidemia;Multimorbid;
Ribosomal scanning and start codon recognition	58	Up	4.344E-07	1.559E-05	AnaemiaExtracard_arteriopathy;Hyperlipidemia;Multimorbid;Renal_Disease
Pyruvate metabolism and Citric Acid (TCA) cycle	50	Down	7.485E-07	2.623E-05	CPD;DiabetesHypertension;Multimorbid;Renal_Disease
HDACs deacetylate histones	82	Up	9.991E-07	3.451E-05	Extracard_arteriopathyHypertension;Multimorbid;
Influenza Infection	154	Up	1.024E-06	3.486E-05	AnaemiaExtracard_arteriopathy;Hyperlipidemia;Multimorbid;Renal_Disease
Meiotic recombination	71	Up	1.270E-06	4.266E-05	Extracard_arteriopathyMultimorbid;
Mitochondrial translation termination	89	Down	1.293E-06	4.283E-05	CPD;DiabetesHypertension;Multimorbid;Renal_Disease
Complex I biogenesis	55	Down	1.325E-06	4.331E-05	CPD;Stroke;DiabetesHyperlipidemia;Multimorbid;Renal_Disease
Mitochondrial translation	95	Down	1.409E-06	4.544E-05	CPD;DiabetesHypertension;Multimorbid;Renal_Disease
HCMV Late Events	104	Up	1.482E-06	4.716E-05	Extracard_arteriopathyMultimorbid;
RNA processing (GO:0006396)	74	Up	1.512E-06	4.748E-05	AnaemiaDiabetesHypertension;Multimorbid;Obese;
Mitochondrial translation elongation	89	Down	1.563E-06	4.846E-05	CPD;DiabetesHypertension;Multimorbid;Renal_Disease
Mitochondrial translation initiation	89	Down	2.009E-06	6.148E-05	CPD;DiabetesHypertension;Multimorbid;Renal_Disease
RNA Polymerase I Promoter Escape	80	Up	2.568E-06	7.710E-05	Extracard_arteriopathyMultimorbid;
Formation of the beta-catenin:TCF transactivating complex	79	Up	2.583E-06	7.710E-05	Multimorbid;
Cristae formation	31	Down	2.961E-06	8.732E-05	CPD;Stroke;DiabetesHypertension;Multimorbid;Renal_Disease
Pre-NOTCH Transcription and Translation	81	Up	3.913E-06	1.140E-04	DiabetesMultimorbid;
Mitochondrial protein import	65	Down	5.589E-06	1.609E-04	CPD;DiabetesHypertension;Multimorbid;Renal_Disease
Signaling by the B Cell Receptor (BCR)	146	Up	5.972E-06	1.699E-04	AnaemiaDiabetes;Extracard_arteriopathy;Hyperlipidemia;Hypertension;Multimorbid;Obese;
Glyoxylate metabolism and glycine degradation	23	Down	6.320E-06	1.777E-04	CPD;DiabetesMultimorbid;Renal_Disease
RUNX1 regulates genes involved in megakaryocyte differentiation and platelet function	83	Up	6.709E-06	1.865E-04	DiabetesHypertension;Multimorbid;
GO:0005730	76	Up	8.848E-06	2.431E-04	AnaemiaDiabetesHypertension;Multimorbid;Obese;
B-WICH complex positively regulates rRNA expression	80	Up	9.314E-06	2.448E-04	Multimorbid;
Cleavage of the damaged pyrimidine	52	Up	9.267E-06	2.448E-04	Multimorbid;
Depyrimidination	52	Up	9.267E-06	2.448E-04	Multimorbid;
Recognition and association of DNA glycosylase with site containing an affected pyrimidine	52	Up	9.267E-06	2.448E-04	Multimorbid;
Base-Excision Repair, AP Site Formation	54	Up	1.163E-05	3.024E-04	Multimorbid;
Utilization of Ketone Bodies	4	Down	1.219E-05	3.136E-04	Multimorbid;
Transcriptional regulation by small RNAs	96	Up	1.262E-05	3.211E-04	Multimorbid;
Mitochondrial biogenesis	93	Down	1.713E-05	4.314E-04	DiabetesHypertension;Multimorbid;
Inhibition of DNA recombination at telomere	59	Up	1.765E-05	4.400E-04	Multimorbid;
Interleukin-10 signaling	35	Up	2.106E-05	5.195E-04	CPD;Stroke;Diabetes;Extracard_arteriopathy;Hyperlipidemia;Multimorbid;
Major pathway of rRNA processing in the nucleolus and cytosol	181	Up	2.561E-05	6.254E-04	AnaemiaExtracard_arteriopathy;Hyperlipidemia;Multimorbid;Renal_Disease
Meiotic synapsis	62	Up	2.910E-05	7.038E-04	Multimorbid;
Branched-chain amino acid catabolism	21	Down	3.198E-05	7.657E-04	DiabetesHypertension;Multimorbid;
FCERI mediated NF-kB activation	117	Up	3.269E-05	7.750E-04	Extracard_arteriopathy;Hyperlipidemia;Hypertension;Multimorbid;
rRNA processing in the nucleus and cytosol	191	Up	3.635E-05	8.535E-04	AnaemiaExtracard_arteriopathy;Hyperlipidemia;Multimorbid;Renal_Disease
Deposition of new CENPA-containing nucleosomes at the centromere	59	Up	3.856E-05	8.879E-04	Multimorbid;
Nucleosome assembly	59	Up	3.856E-05	8.879E-04	Multimorbid;
Positive epigenetic regulation of rRNA expression	95	Up	4.678E-05	1.067E-03	Multimorbid;
Negative epigenetic regulation of rRNA expression	98	Up	5.318E-05	1.202E-03	Multimorbid;
NoRC negatively regulates rRNA expression	95	Up	6.051E-05	1.355E-03	Multimorbid;
Meiosis	95	Up	6.713E-05	1.489E-03	Multimorbid;
Keratan sulfate degradation	12	Up	6.884E-05	1.513E-03	Diabetes;Extracard_arteriopathyMultimorbid;
Infectious disease	800	Up	7.086E-05	1.544E-03	Extracard_arteriopathy;Hyperlipidemia;Multimorbid;
Regulation of expression of SLITs and ROBOs	162	Up	7.512E-05	1.607E-03	AnaemiaExtracard_arteriopathy;Hyperlipidemia;Multimorbid;Renal_Disease
Oxidative Stress Induced Senescence	110	Up	7.460E-05	1.607E-03	Multimorbid;
HCMV Early Events	121	Up	7.608E-05	1.614E-03	Multimorbid;
Pre-NOTCH Expression and Processing	96	Up	8.849E-05	1.861E-03	Multimorbid;
Activation of anterior HOX genes in hindbrain development during early embryogenesis	101	Up	9.237E-05	1.909E-03	Multimorbid;
Activation of HOX genes during differentiation	101	Up	9.237E-05	1.909E-03	Multimorbid;
DNA Damage/Telomere Stress Induced Senescence	69	Up	9.486E-05	1.944E-03	Multimorbid;
RNA Polymerase I Transcription	100	Up	1.173E-04	2.384E-03	Multimorbid;
RNA Polymerase I Promoter Clearance	99	Up	1.261E-04	2.541E-03	Multimorbid;
RMTs methylate histone arginines	69	Up	1.383E-04	2.764E-03	Multimorbid;
rRNA processing	203	Up	1.431E-04	2.837E-03	AnaemiaExtracard_arteriopathy;Hyperlipidemia;Multimorbid;Renal_Disease
Class A/1 (Rhodopsin-like receptors)	153	Up	1.566E-04	3.079E-03	Diabetes;Extracard_arteriopathy;Hyperlipidemia;Multimorbid;
Reproduction	104	Up	1.848E-04	3.603E-03	Multimorbid;
Defective CHST6 causes MCDC1	7	Up	1.999E-04	3.866E-03	DiabetesMultimorbid;
HCMV Infection	145	Up	2.101E-04	4.032E-03	Multimorbid;
Neutrophil degranulation	418	Up	2.393E-04	4.557E-03	Diabetes;Extracard_arteriopathy;Hyperlipidemia;Multimorbid;
Collagen biosynthesis and modifying enzymes	58	Up	2.418E-04	4.569E-03	CPD;Stroke;DiabetesHypertension;Multimorbid;Renal_Disease
Extracellular matrix organization	244	Up	2.888E-04	5.414E-03	CPD;Stroke;Diabetes;Extracard_arteriopathyHypertension;Multimorbid;Renal_Disease
HDMs demethylate histones	46	Up	2.941E-04	5.470E-03	Multimorbid;
Metal sequestration by antimicrobial proteins	3	Up	3.497E-04	6.455E-03	Multimorbid;
Innate Immune System	890	Up	3.658E-04	6.700E-03	Diabetes;Extracard_arteriopathy;Hyperlipidemia;Multimorbid;
GPCR ligand binding	210	Up	3.741E-04	6.801E-03	Diabetes;Extracard_arteriopathyMultimorbid;
Modulation by Mtb of host immune system	7	Up	4.125E-04	7.443E-03	DiabetesHyperlipidemia;Multimorbid;
Collagen formation	76	Up	4.643E-04	8.316E-03	CPD;Stroke;DiabetesHypertension;Multimorbid;Renal_Disease
Regulation of innate immune responses to cytosolic DNA	14	Up	4.826E-04	8.581E-03	AnaemiaDiabetesMultimorbid;
Signaling by ROBO receptors	206	Up	5.008E-04	8.838E-03	AnaemiaExtracard_arteriopathy;Hyperlipidemia;Multimorbid;Renal_Disease
Fc epsilon receptor (FCERI) signaling	167	Up	5.391E-04	9.446E-03	DiabetesHyperlipidemia;Hypertension;Multimorbid;
RUNX1 regulates transcription of genes involved in differentiation of HSCs	113	Up	6.116E-04	1.064E-02	Extracard_arteriopathyMultimorbid;Renal_Disease
Formation of ATP by chemiosmotic coupling	18	Down	6.197E-04	1.070E-02	Stroke;Multimorbid;Renal_Disease
PTK6 Regulates RTKs and Their Effectors AKT1 and DOK1	8	Up	6.588E-04	1.130E-02	Extracard_arteriopathyMultimorbid;
Defective B4GALT1 causes B4GALT1-CDG (CDG-2d)	7	Up	7.794E-04	1.327E-02	DiabetesMultimorbid;
Antimicrobial peptides	19	Up	8.527E-04	1.442E-02	Diabetes;Extracard_arteriopathy;Hyperlipidemia;Multimorbid;
NRIF signals cell death from the nucleus	16	Up	8.659E-04	1.454E-02	Multimorbid;
Keratan sulfate/keratin metabolism	30	Up	8.754E-04	1.460E-02	Multimorbid;
Platelet degranulation	110	Up	8.973E-04	1.486E-02	DiabetesHyperlipidemia;Multimorbid;
Gene Silencing by RNA	121	Up	9.538E-04	1.569E-02	Multimorbid;
Maturation of protein E	4	Up	1.152E-03	1.882E-02	Multimorbid;
Dectin-2 family	13	Up	1.163E-03	1.887E-02	DiabetesMultimorbid;
Regulation of TLR by endogenous ligand	13	Up	1.193E-03	1.923E-02	DiabetesMultimorbid;
Endosomal/Vacuolar pathway	9	Up	1.317E-03	2.108E-02	Diabetes;Extracard_arteriopathyHypertension;Multimorbid;
Hemostasis	541	Up	1.329E-03	2.114E-02	CPD;DiabetesHyperlipidemia;Multimorbid;
Estrogen-dependent gene expression	135	Up	1.411E-03	2.230E-02	Multimorbid;
Base Excision Repair	82	Up	1.471E-03	2.309E-02	Multimorbid;
HATs acetylate histones	128	Up	1.540E-03	2.403E-02	Multimorbid;
Response to elevated platelet cytosolic Ca2+	114	Up	1.604E-03	2.438E-02	DiabetesHyperlipidemia;Multimorbid;
Epigenetic regulation of gene expression	136	Up	1.624E-03	2.438E-02	Multimorbid;
ribonuclease MRP activity GO:0000171	2	Up	1.616E-03	2.438E-02	Multimorbid;
ribonuclease MRP complex GO:0000172	2	Up	1.616E-03	2.438E-02	Multimorbid;
rRNA processing GO:0006364	2	Up	1.616E-03	2.438E-02	Multimorbid;
RNA phosphodiester bond hydrolysis GO:0090501	2	Up	1.616E-03	2.438E-02	Multimorbid;
Cellular Senescence	177	Up	1.773E-03	2.647E-02	Multimorbid;
Progressive trimming of alpha-1,2-linked mannose residues from Man9/8/7GlcNAc2 to produce Man5GlcNAc2	3	Up	1.808E-03	2.682E-02	Multimorbid;
Mitotic Prophase	130	Up	1.846E-03	2.722E-02	Multimorbid;
Telomere Maintenance	102	Up	1.888E-03	2.766E-02	Multimorbid;
Defective CYP1B1 causes Glaucoma	1	Up	2.024E-03	2.948E-02	Multimorbid;
Regulation of Insulin-like Growth Factor (IGF) transport and uptake by Insulin-like Growth Factor Binding Proteins (IGFBPs)	88	Up	2.047E-03	2.964E-02	Stroke;Diabetes;Extracard_arteriopathy;Hyperlipidemia;Multimorbid;Renal_Disease
Recruitment and ATM-mediated phosphorylation of repair and signaling proteins at DNA double strand breaks	70	Up	2.121E-03	3.052E-02	Multimorbid;
E3 ubiquitin ligases ubiquitinate target proteins	55	Up	2.158E-03	3.087E-02	Multimorbid;
Elastic fibre formation	43	Up	2.196E-03	3.123E-02	Stroke;Diabetes;Extracard_arteriopathyMultimorbid;
Molecules associated with elastic fibres	36	Up	2.245E-03	3.174E-02	Stroke;Diabetes;Extracard_arteriopathyMultimorbid;
Post-translational protein phosphorylation	79	Up	2.303E-03	3.238E-02	Stroke;Diabetes;Extracard_arteriopathy;Hyperlipidemia;Multimorbid;
ESR-mediated signaling	197	Up	2.379E-03	3.325E-02	Multimorbid;
The AIM2 inflammasome	2	Up	2.423E-03	3.368E-02	Multimorbid;
TGF-beta receptor signaling in EMT (epithelial to mesenchymal transition)	16	Up	2.444E-03	3.377E-02	Hyperlipidemia;Multimorbid;
IRAK2 mediated activation of TAK1 complex upon TLR7/8 or 9 stimulation	15	Up	2.751E-03	3.779E-02	Multimorbid;
ECM proteoglycans	66	Up	3.046E-03	4.161E-02	CPD;Stroke;DiabetesHypertension;Multimorbid;Renal_Disease
DNA Double Strand Break Response	71	Up	3.109E-03	4.223E-02	Multimorbid;
TNFs bind their physiological receptors	18	Up	3.194E-03	4.315E-02	Diabetes;Extracard_arteriopathyMultimorbid;
EPHB-mediated forward signaling	41	Up	3.233E-03	4.343E-02	DiabetesHyperlipidemia;Multimorbid;
Cross-presentation of particulate exogenous antigens (phagosomes)	8	Up	3.345E-03	4.469E-02	CPD;DiabetesMultimorbid;

Table S7 STROBE Statement—Checklist of items that should be included in reports of case-control studies

	Item No	Recommendation	Page No
**Title and abstract**	1	(*a*) Indicate the study's design with a commonly used term in the title or the abstract	1-2
		(*b*) Provide in the abstract an informative and balanced summary of what was done and what was found	1-2
**Introduction**			
Background/rationale	2	Explain the scientific background and rationale for the investigation being reported	3
Objectives	3	State specific objectives, including any prespecified hypotheses	3
**Methods**			
Study design	4	Present key elements of study design early in the paper	4
Setting	5	Describe the setting, locations, and relevant dates, including periods of recruitment, exposure, follow-up, and data collection	4
Participants	6	(*a*) Give the eligibility criteria, and the sources and methods of case ascertainment and control selection. Give the rationale for the choice of cases and controls	4
		(*b*) For matched studies, give matching criteria and the number of controls per case	N/A
Variables	7	Clearly define all outcomes, exposures, predictors, potential confounders, and effect modifiers. Give diagnostic criteria, if applicable	4
Data sources/ measureme	8*	For each variable of interest, give sources of data and details of methods of assessment (measurement). Describe comparability of assessment methods if there is more than one group	4-8
Bias	9	Describe any efforts to address potential sources of bias	4-8
Study size	10	Explain how the study size was arrived at	4-8
Quantitative variables	11	Explain how quantitative variables were handled in the analyses. If applicable, describe which groupings were chosen and why	4-8
Statistical methods	12	(*a*) Describe all statistical methods, including those used to control for confounding	8-9, Supplemental Appendix
		(*b*) Describe any methods used to examine subgroups and interactions	N/A
		(*c*) Explain how missing data were addressed	4-8
		(*d*) If applicable, explain how matching of cases and controls was addressed	N/A
		(*e*) Describe any sensitivity analyses	N/A
**Results**			
Participants	13*	(a) Report numbers of individuals at each stage of study—eg numbers potentially eligible, examined for eligibility, confirmed eligible, included in the study, completing follow-up, and analysed	9
		(b) Give reasons for non-participation at each stage	9
		(c) Consider use of a flow diagram	**Figure 1**
Descriptive data	14*	(a) Give characteristics of study participants (eg demographic, clinical, social) and information on exposures and potential confounders	**Table 1, Table S2**
		(b) Indicate number of participants with missing data for each variable of interest	**Table 1, Table S2**
Outcome data	15*	Report numbers in each exposure category, or summary measures of exposure	**Table 1, Table S2**
Main results	16	(*a*) Give unadjusted estimates and, if applicable, confounder-adjusted estimates and their precision (eg, 95% confidence interval). Make clear which confounders were adjusted for and why they were included	9-15
		(*b*) Report category boundaries when continuous variables were categorized	9-15
		(*c*) If relevant, consider translating estimates of relative risk into absolute risk for a meaningful time period	N/A
Other analyses	17	Report other analyses done—eg analyses of subgroups and interactions, and sensitivity analyses	N/A
**Discussion**			
Key results	18	Summarise key results with reference to study objectives	16-18
Limitations	19	Discuss limitations of the study, taking into account sources of potential bias or imprecision. Discuss both direction and magnitude of any potential bias	16-18
Interpretation	20	Give a cautious overall interpretation of results considering objectives, limitations, multiplicity of analyses, results from similar studies, and other relevant evidence	16-18
Generalisability	21	Discuss the generalisability (external validity) of the study results	18
**Other information**			
Funding	22	Give the source of funding and the role of the funders for the present study and, if applicable, for the original study on which the present article is based	19
